# Grape Leaf Black Rot Detection Based on Super-Resolution Image Enhancement and Deep Learning

**DOI:** 10.3389/fpls.2021.695749

**Published:** 2021-06-29

**Authors:** Jiajun Zhu, Man Cheng, Qifan Wang, Hongbo Yuan, Zhenjiang Cai

**Affiliations:** College of Mechanical and Electrical Engineering, Hebei Agricultural University, Baoding, China

**Keywords:** small targets, grape black rot, super-resolution, convolutional neural network, deep-learning

## Abstract

The disease spots on the grape leaves can be detected by using the image processing and deep learning methods. However, the accuracy and efficiency of the detection are still the challenges. The convolutional substrate information is fuzzy, and the detection results are not satisfactory if the disease spot is relatively small. In particular, the detection will be difficult if the number of pixels of the spot is <32 × 32 in the image. In order to effectively address this problem, we present a super-resolution image enhancement and convolutional neural network-based algorithm for the detection of black rot on grape leaves. First, the original image is up-sampled and enhanced with local details using the bilinear interpolation. As a result, the number of pixels in the image increase. Then, the enhanced images are fed into the proposed YOLOv3-SPP network for detection. In the proposed network, the IOU (Intersection Over Union, IOU) in the original YOLOv3 network is replaced with GIOU (Generalized Intersection Over Union, GIOU). In addition, we also add the SPP (Spatial Pyramid Pooling, SPP) module to improve the detection performance of the network. Finally, the official pre-trained weights of YOLOv3 are used for fast convergence. The test set test_pv from the Plant Village and the test set test_orchard from the orchard field were used to evaluate the network performance. The results of test_pv show that the grape leaf black rot is detected by the YOLOv3-SPP with 95.79% detection accuracy and 94.52% detector recall, which is a 5.94% greater in terms of accuracy and 10.67% greater in terms of recall as compared to the original YOLOv3. The results of test_orchard show that the method proposed in this paper can be applied in field environment with 86.69% detection precision and 82.27% detector recall, and the accuracy and recall were improved to 94.05 and 93.26% if the images with the simple background. Therefore, the detection method proposed in this work effectively solves the detection task of small targets and improves the detection effectiveness of the grape leaf black rot.

## Introduction

Grapes are one of the most commonly grown economic fruits in the world, which are often used in the production of wine, fermented beverages, and raisins (Kole et al., 2014). The larger the area used for the cultivation of grapes, the larger is the scale of the disease affecting the grapes and consequently, the probability of economic loss is higher as well. Generally, the early stages of grape diseases are evident on the leaves. Therefore, the leaves can be used for the identification and diagnosis of diseases during the early stage. Black rot is one of the most common grape diseases in the world (Molitor and Berkelmann-Loehnertz, 2011). Black rot is a fungal disease that exhibits a black spot on the grape leaves. This spot is relatively smaller as compared to the size of the leaves. This disease usually appears during the moist spring season and early summer. The black spot affects a wide area of the leaves (Pearson and Goheen, 1989). Currently, a manual method is mainly used for the identification of this disease. In this method, the farmers use their extensive experience to make a rough identification of the disease. However, it is notable that this approach not only requires a lot of manual labor but is also susceptible to the subjective factors (Chen et al., 2020). In order to ensure the grape production and economic well-being of the farmers, rapid and effective detection of black rot on grape leaves is important for the farming industry.

Currently, the machine vision technologies are widely used in various fields for detection and classification tasks. In the early stages of research on grape leaf diseases using machine learning, Agrawal et al. (2017) used SVM for the classification of grape diseases using leaves. The proposed method included image resizing, image enhancement, and image smoothing to save the memory and reduce the processing time. Waghmare et al. (2016) proposed the local binary patterns and machine learning for the detection and classification of grape diseases. The authors uniformly resize the images to 226 226 before further processing. In addition, the images are transformed from the RGB to HSV color space and the background subtraction is used to remove the unwanted background in the images. Es-Saady et al. (2016) proposed the automatic identification of plant diseases using leaves based on a serial combination of two SVM classifiers. The authors used different colors as the classification criterion in the first classifier. Then, in the second classifier, the shape and texture features of colored leaves were used for classification. Although the traditional machine learning algorithms have made some achievements in grape leaf disease detection, these methods require manual feature extraction. In addition, the accuracy of disease detection needs to be improved as well.

Recently, deep learning has been extensively used for the purpose of detection and classification in various applications. Felzenszwalb et al. (2008) proposed a region-based convolutional neural network (CNN) for target detection. There are few region-based detection algorithms presented in literature, such as RCNN (Girshick et al., 2014), Fast-RCNN (Girshick, 2015), and Faster-RCNN (Ren et al., 2017). Similarly, the well-known end-to-end detection algorithms include SSD (Liu et al., 2016) and YOLO (Redmon et al., 2016; Redmon and Farhadi, 2017, 2018). The CNNs have been used for the detection of grape diseases. Wagh et al. (2019) proposed an automatic grape disease identification system based on the AlexNet. This method has the ability to detect the bacterial spots and powdery mildew with an accuracy of 98.23%. Ji et al. (2020) proposed a unified model based on multiple CNNs for automatic identification of grape leaf diseases. This method was used for the classification of black rot, esca, isariopsis leaf spot, and healthy images. The average validation accuracy of this method is 99.17% and the test accuracy is 98.57%. Liu et al. (2020) proposed an improved CNN for the identification of grape leaf diseases. The authors use this technique to identify anthracnose, brown spot, mites, black rot, downy mildew, and leaf blight. This method utilizes the depth-separable convolution instead of standard convolutional layers. As a result, this method achieves higher convergence speed and accuracy. Xie et al. (2020) proposed a rapid detector for grape leaf diseases based on deep learning. This technique automatically extracts the disease spot features and has the ability to detect four common grape leaf diseases with high accuracy and fast detection speed. Alessandrini et al. (2021) proposed a grapevine leaves dataset for early detection and classification of Esca disease in vineyards through machine learning.

However, it is noteworthy that the existing networks, such as AlexNet, RCNN, and Fast-RCNN, suffer from non-negligible miss-detections and low recall for the small spots. For instance, if the spot pixels are <32 32 (Bosquet et al., 2018), or when the image resolution is not high. According to the definition of the international organization SPIE, a small target is a target area <80 pixels in a 256 × 256 image, that is, the target whose pixel proportion is <0.12% of the total image pixels. The small target recognition is still a challenge.

The super-resolution is an image processing method which is commonly used in the field of remote sensing (Xie et al., 2017; Arun et al., 2019), feature extraction, non-linear mapping, and image reconstruction. This technique has the ability to make small targets on the original image clear, even after the application of convolution. Bai et al. (2018a,b) applied the super-resolution for small target detection. The results show that this method effectively enhances the information of small targets, while improving the detection accuracy. Noh et al. (2019) proposed an accurate monitoring of feature super-resolution for small target detection. This method demonstrates the importance of using the appropriate high-resolution target features that share the same relative field of perception as the low-resolution input features to provide direct monitoring. Rabbi (2020) proposed remote sensing image small target detection based on an end-to-end edge-enhanced neural network and target detector network. The authors present the stochastic resonant network which has a faster response and yields the best results for small targets on satellite images.

In order to improve the detection accuracy of low-resolution small targets in the grape black rot spot detection, in this work, we propose a super-resolution image enhancement and deep learning-based detection of black rot in grape leaves. The proposed method used an improved loss function for performing detections. In addition, we also propose the spatial pyramid pooling (SPP) module in the detection network, which effectively increases the reception range of the backbone features and significantly separates the most important contextual features. Moreover, the proposed method improves the target recall and accuracy as compared with the max pooling technique.

The major contributions of this work are as follows.

- We perform enhancement of grape leaves by using the bilinear interpolation.
- We improve the YOLOv3 network. The IOU in the original yolo YOLOv3 network is replaced with GIOU. In addition, we also add the SPP module to improve the detection performance of the network.
- We perform experiments and analysis to evaluate the effectiveness of the super-resolution image enhancement and improved YOLOv3 network for grape black rot detection.

## Materials and Methods

### Data Set and Test Environment Setup

The data used to perform experiments in this work is the open dataset Plant Village. We select 1,180 images of grapevine leaf black rot for disease detection. We use LabelImg for annotating the diseased parts of the leaves. The average number of diseases present in an image is around 15, with more than 17,000 detection targets present in total. Before starting the training process, we divide 1,180 images into training and test sets. We select 1,072 images for training the network and 108 images as the test set for evaluating the network, which was named test_pv. In addition, 108 images of grape leaves with black rot spots in the orchard environment were collected as an extra test set, which was named test_orchard. We further divide the training set into two parts during the process of network training, namely training set and validation set. The division ratio of training and validation sets is 9:1. In the convolutional neural network, the training set is used for model fitting, and the validation set is a separate sample set in the process of model training, which can be used to adjust the super parameters of the model and to preliminarily evaluate the ability of the model. The test set is used to evaluate the generalization ability of the final model. In this work, the number of epochs is 200, the input batch is 8, the learning rate is 0.001, and the size of the input image is 256 × 256. The coco dataset format is used for the dataset used in this work. We use the pre-trained model weights to accelerate the convergence. We conduct the simulations on Windows 10 based on the pytorch deep learning framework. The computer on which the tests are conducted contains 8GB GPU GeForce GTX 1070Ti and an AMD Ryzen 5 1600X Six-Core Processor. The Python language is used for programming.

### Super-Resolution Enhancement of Grape Leaf Images

**Figure 1:**
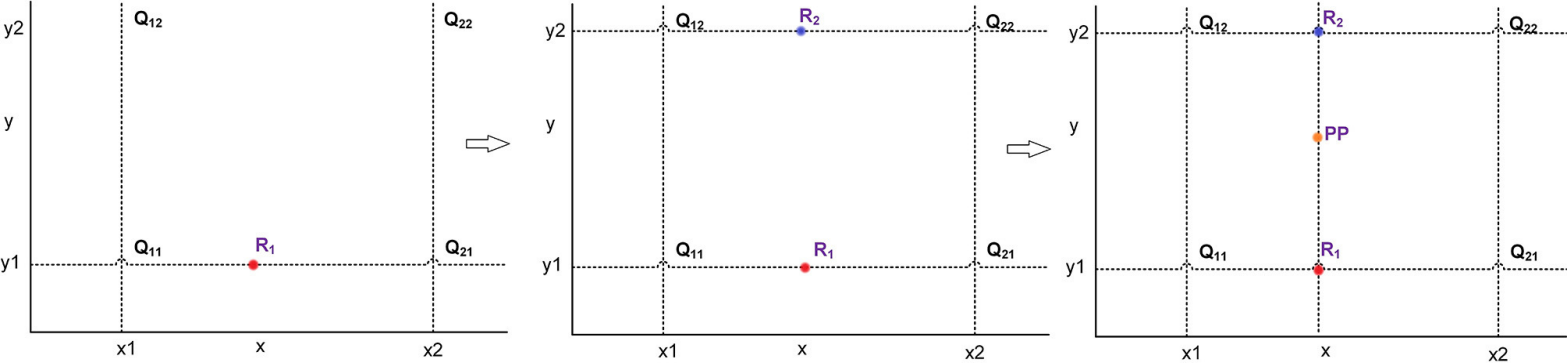
The process of bilinear interpolation.

In order to improve the resolution of the original image, we use a software method to produce a single high-quality and high-resolution image from a set of low-quality, low-resolution images. This method of transforming images is known as the super-resolution reconstruction (Shen et al., 2009). There are two types of image super-resolution reconstruction techniques. In the first technique, we synthesize a high-resolution image from multiple low-resolution images. In second technique, we acquire a high-resolution image from a single low-resolution image. The super-resolution techniques are divided into three categories, namely interpolation-based super-resolution, reconstruction-based super-resolution, and learning-based super-resolution. The interpolation-based super-resolution is relatively simple, and it is also widely used in various techniques. In reconstruction-based super-resolution, the main idea is to map the low-resolution images to high-resolution images. However, this technique is computationally expensive and requires a lot of computational resources. The learning-based super-resolution is implemented on the basis of convolutional neural networks (CNNs). In this work, we use the widely used interpolation method for image super-resolution. This technique is able to achieve results similar to the learning-based super-resolution images without requiring extensive computational resources. The bilinear interpolation (BL) is used for image super-resolution, which means that the original image is up-sampled for scaling operation. This is a technique for image scaling, which is mainly divided into two linear interpolation steps. First, we interpolate in the x-direction to find R_1_ and R_2_. The R_1_ is obtained according to the values of Q_11_ and Q_21_, and the R_2_ is obtained according to the values of Q_12_ and Q_22_. Then, the result of x-direction interpolation R1 and R2 are used to find the output of y-direction interpolation PP. The process of bilinear interpolation is shown in Figure 1.

In Figure 1, Q_11_(x_1_, y_1_), Q_12_(x_1_, y_2_), Q_21_(x_2_, y_1_), and Q_22_(x_2_, y_2_) represent the original pixel points. The first interpolation R1 along x-axis is calculated by using Q11 and Q21. The second interpolation R2 along x-axis is calculated by using Q12 and Q22. The interpolation result PP(x, y) along y-axis is calculated by using R1 and R2. The interpolation calculation process is shown in (1). The pixel points in the image are added in such a way that the image is transformed from low resolution to high resolution and super-resolution image is obtained.

(1) $$\{\begin{array}{l} f(R_{1})=\frac{x_{2}-x}{x_{2}-x_{1}}\;f(Q_{11})\;+\frac{x-x_{1}}{x_{2}-x_{1}}\;f(Q_{21})\\ f(R_{2})=\frac{x_{2}-x}{x_{2}-x_{1}}\;f(Q_{12})\;+\frac{x-x_{1}}{x_{2}-x_{1}}\;f(Q_{22})\\ f(PP)=\frac{y_{2}-y}{y_{2}-y_{1}}\;f(R_{1})\;+\frac{y-y_{1}}{y_{2}-y_{1}}\;f(R_{2}) \end{array}$$

Where, f(Q_11_), f(Q_21_), f(R_1_), f(R_2_), and f(PP) represent the value of corresponding points, respectively.

### Deep Learning Method for Grape Leaf Spot Detection

In order to improve the accuracy of grapevine leaf black rot spot detection, we deign an improved YOLOv3 network. The YOLO (Redmon et al., 2016) is a typical CNN model used in target recognition, and YOLO v3 (Redmon and Farhadi, 2018) is the third version of the YOLO, which has advantages over v1 and v2 for the detection of small targets. In the improved network, we replace the loss function IOU (Intersection Over Union, IOU) with GIOU (Generalized Intersection Over Union, GIOU), and GIOU provides better regression boxes during the training process. In addition, we introduce SPP (Spatial Pyramid Pooling, SPP) before the output of feature layer, which is conducive to the detection of large differences in the target size in the image. We enhance the images by applying the linear interpolation before using the image as an input to the CNN, which is super-resolution of the image and can improve the effect of target detection.

### Loss Function GIOU

In the original YOLOv3 target detection network, the engagement ratio IOU of the bounding box and the ground truth is used as the loss function. In the improved network proposed in this work, the IOU is replaced by GIOU in the deep learning network. The GIOU is calculated as shown in (2).

(2) $$\text{GIOU=IOU- }\frac{{\text{|A}}_{\text{c }}\text{-U|}}{{\text{|A}}_{\text{c}}\text{|}}$$

where, Ac denotes the area of the minimum closure region of the two boxes, i.e., the ground truth and the predicted bounding box, and U denotes the intersection area of the two boxes. By using the GIOU as a loss function, we avoid the problem caused when the two target boxes have no overlap. So, the gradient is continuously updated and better regression boxes are available during the training process.

### SPP Module

**Figure 2:**
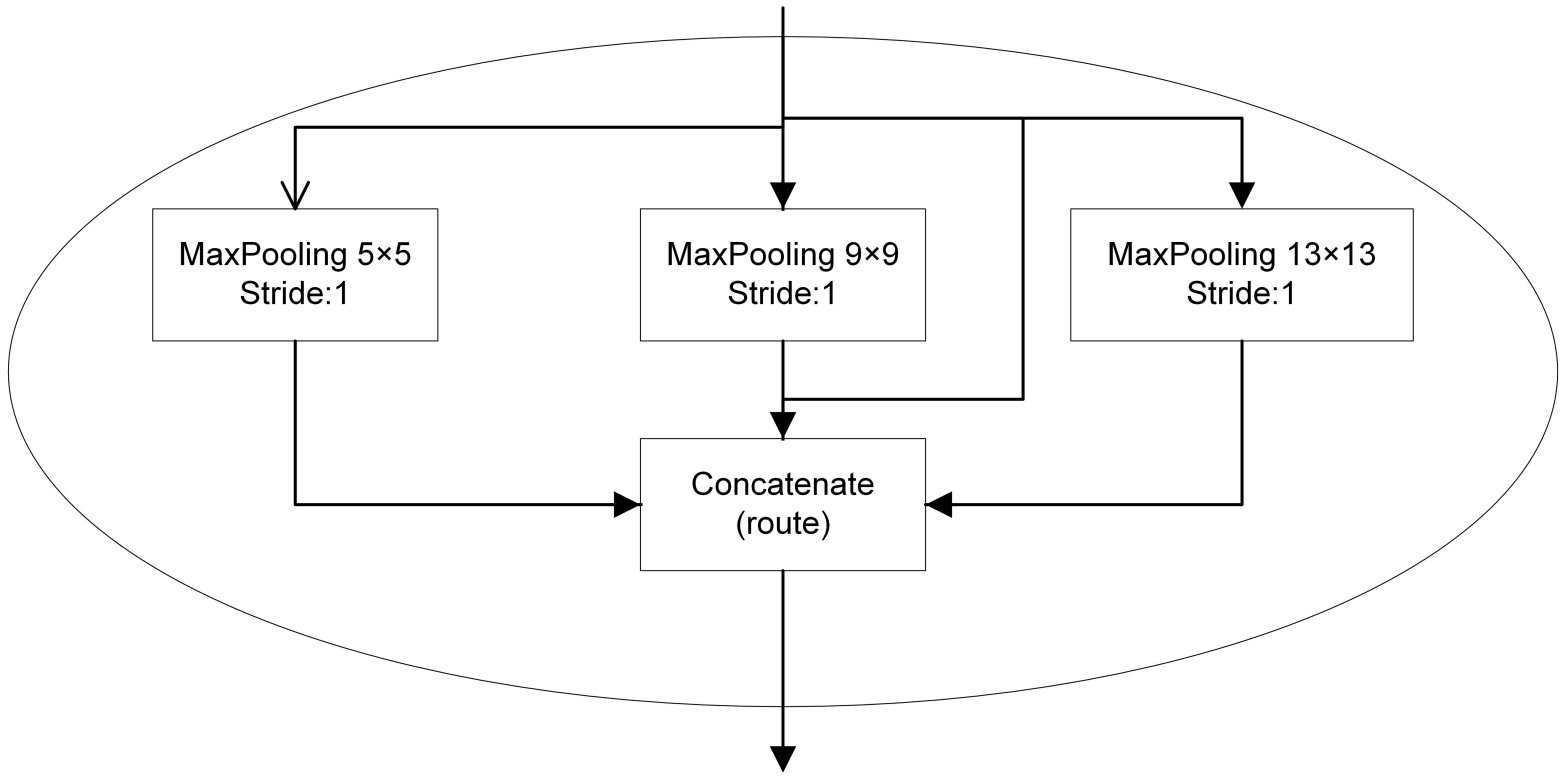
The SPP structure in the improved YOLOv3 proposed in this work.

The SPP works on the idea of the spatial pyramid (He et al., 2014). On the basis of SPP module, we achieve the fusion of local and global features. This feature fusion enhances the expressiveness of the feature map which is conducive in the detection of large differences in the target size in the image. In the YOLOv3-SPP network proposed in this work, the SPP module consists of four parallel branches with kernel sizes of 5 × 5, 9 × 9, 13 × 13, and 16 × 16. In this work, the input of SPP model is the 16 × 16 size image after subsampling, and the output is the fusion of four parallel branches. The SPP module is placed after the 16 × 16 convolutional layer and before the output. This structure is shown in Figure 2.

### Improved YOLOv3-SPP Network Architecture

**Figure 3:**
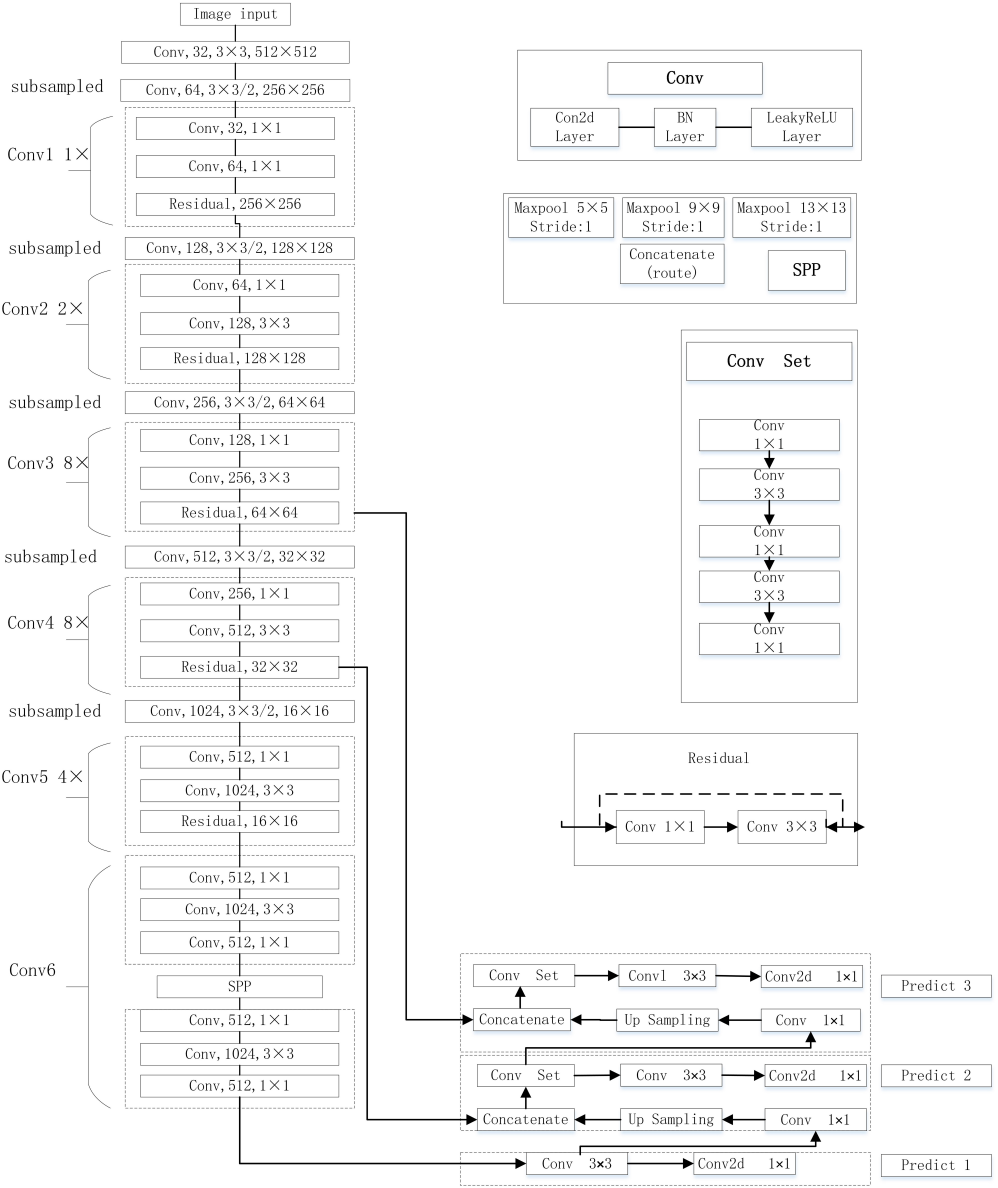
The improved YOLOv3 network proposed in this work.

The YOLOv3-SPP network proposed in this work is implemented by improving the original YOLOv3. The original YOLOv3 network uses Darknet-53 as the backbone network. The Darknet-53 mainly consists of 5 residual blocks. This structure uses the idea of residual neural network, and the idea of FPN (Feature Pyramid Networks, FPN). The up-sampling fusion is adopted to detect the target independently by fusing multiple feature maps at three different scales, including 16 × 16, 32 × 32, and 64 × 64. The size of the minimum prediction frame is 8 × 8 (image size divided by grid size 512/64), which effectively obtains the feature information at low and high levels. This end-to-end network is not only more accurate but is computationally efficient as well. In this work, the SPP module is introduced in the Conv6 layer of the YOLOv3. The YOLOv3-SPP network structure is shown in Figure 3.

### Evaluation Indicators

We use the precision (P) and recall (R) as evaluation metrics. The precision of any algorithm is computed as

(3) $$\text{P= }\frac{\text{TP}}{\text{TP+FP}}\times \text{100}\%$$

where, TP represents the true positives, which is expressed as the number of manually labeled grape disease pixels that overlap with pixels in the region automatically detected by the model as grape disease. FP represents the false positives, which is expressed as the number of pixels in the region manually considered as background, but automatically detected by the model as grape leaf region pixels. We calculate recall by using the following expression.

(4) $$\text{R=}\frac{\text{TP}}{\text{TP+FN}}\times \text{100}\%$$

where, FN denotes the false negatives, which indicates the number of pixels that are manually labeled as the grape leaf area pixels, but are detected by the model as background area pixels.

## Results

### Super-Resolution-Based Image Enhancement Results

**Figure 4:**
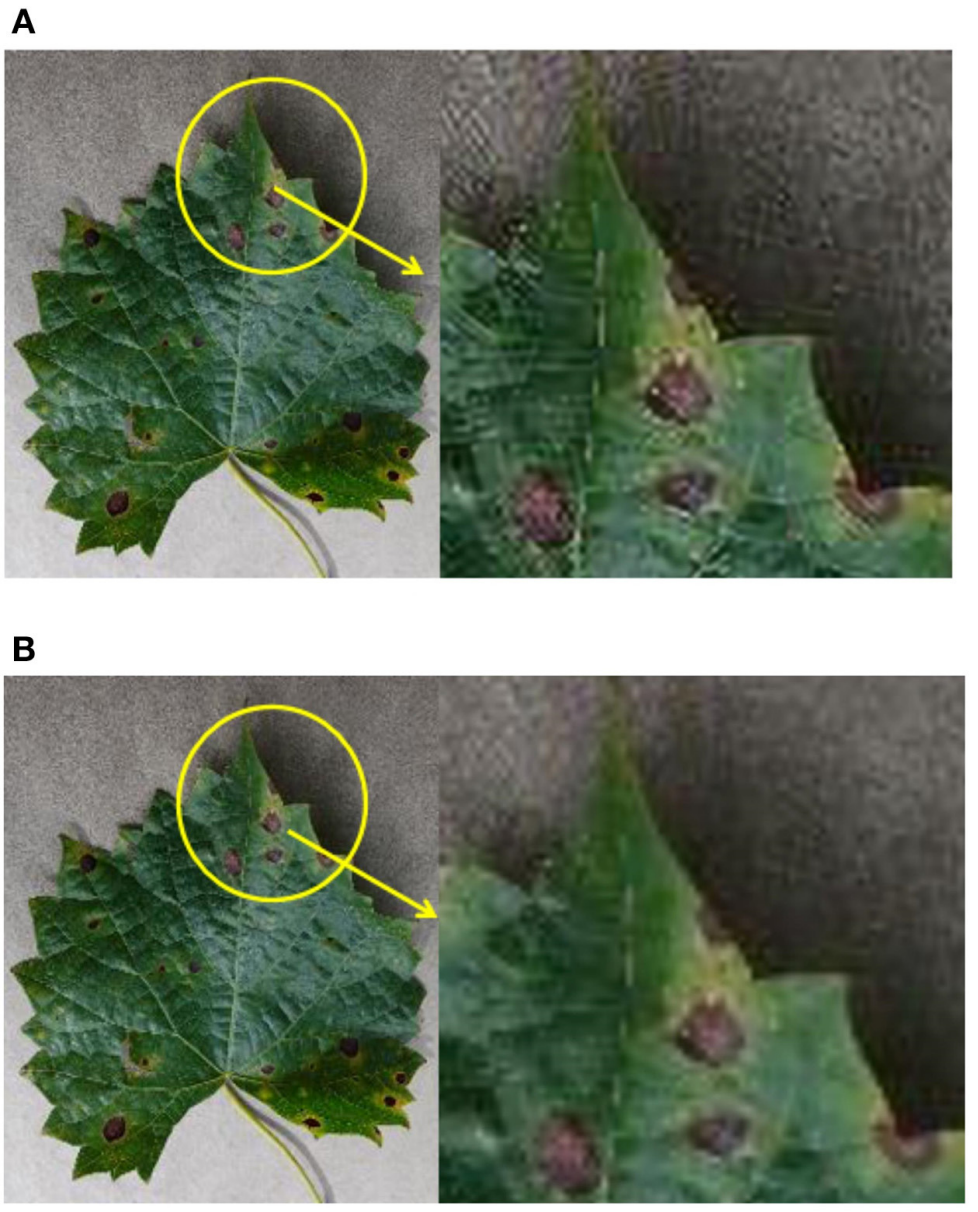
The comparison of the original image and the enhanced image. **(A)** The original image. **(B)** Super-resolution enhanced image.

The size of an image in the original dataset is 256 × 256, and the average image size is around 20 kb. After the application of super-resolution technique, we enhance the input image to a size of 512 × 512. Now, the average image size is 100 kb. Figure 4 shows the comparison between the original image and the resulting enhanced image. It is evident from Figure 4 that the local parts of the image are equally magnified 4 times. The clarity of the image after the super-resolution is significantly higher than that of the original image.

### Original Image YOLOv3-SPP Detection Results

**Figure 5:**
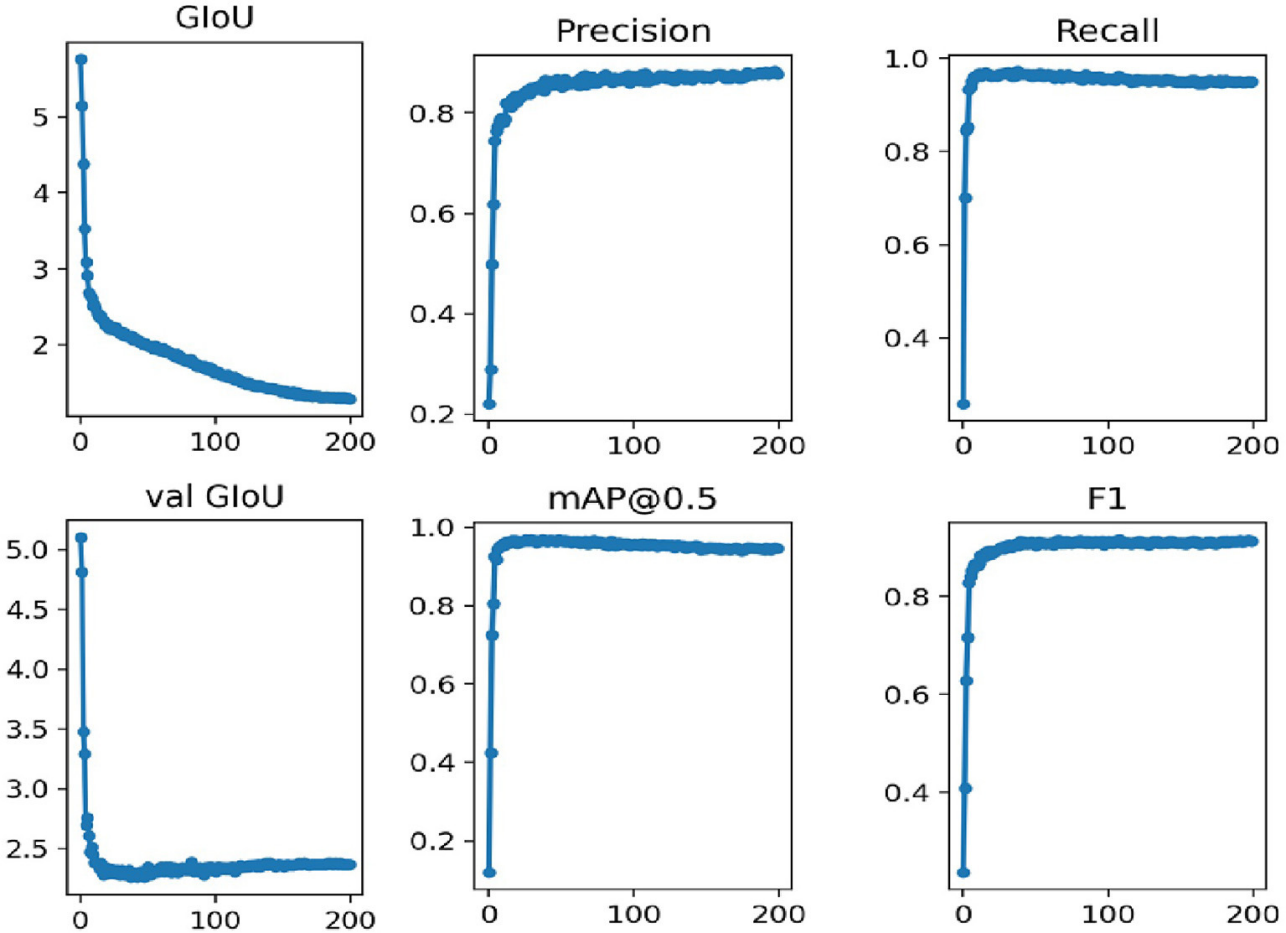
The training performance of the original dataset YOLOv3-SPP network.

In this work, we use the annotated images to train the network. The network is trained for 200 epochs which takes around 6 h. The training results are presented in Figure 5.

In Figure 5, the GIOU denotes the loss function uses in this work, the val GIOU denotes the validated loss function, map@0.5 denotes the average accuracy, and F1 represents an evaluation metric which combines the effect of precision and recall. It is evident from Figure 5 that the network converges rapidly for the first 100 iterations. In addition, the loss function GIOU decreases rapidly until it gets flat around the 100th iteration. The evaluation metrics, i.e., precision, recall, map@0.5, and F1 also become flat at this stage.

### Enhanced Image YOLOv3-SPP Detection Results

**Figure 6:**
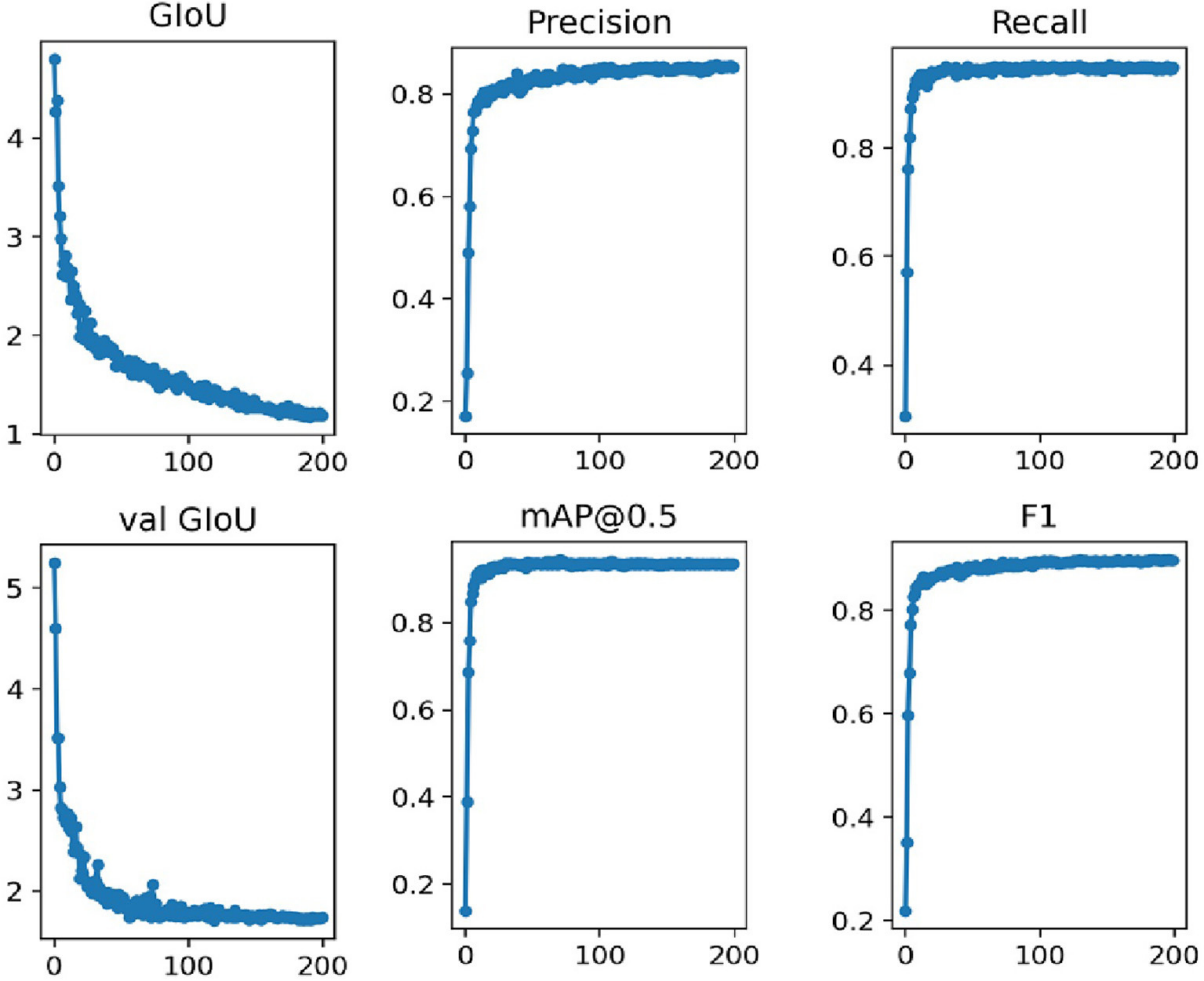
The training performance of YOLOv3-SPP network by using the enhanced dataset.

The enhanced image dataset with annotation information is fed into the YOLOv3-SPP network for training. Please note that the network parameters are the same as used in the case of training the network on original images. This training process consumes around 7 h. The training results are presented in Figure 6. It is evident form Figure 6 that the model is continuously optimized during the epochs, and the loss function decreases rapidly in the initial iteration until it gets flat. This is consistent with training the network using original images which indicates that the improved YOLOv3-SPP network converges rapidly regardless of whether it uses the original images or the enhanced images as the input data. The trend of precision is similar to the case of training the network on original images. However, other evaluation metrics, i.e., recall, map@0.5, and F1 rise rapidly and reach a smooth state at the beginning of the training and show a small decrease after 100 iterations.

## Discussion

### Comparison of the Effect of YOLOv3 Network Improvement Before and After

**Figure 7:**
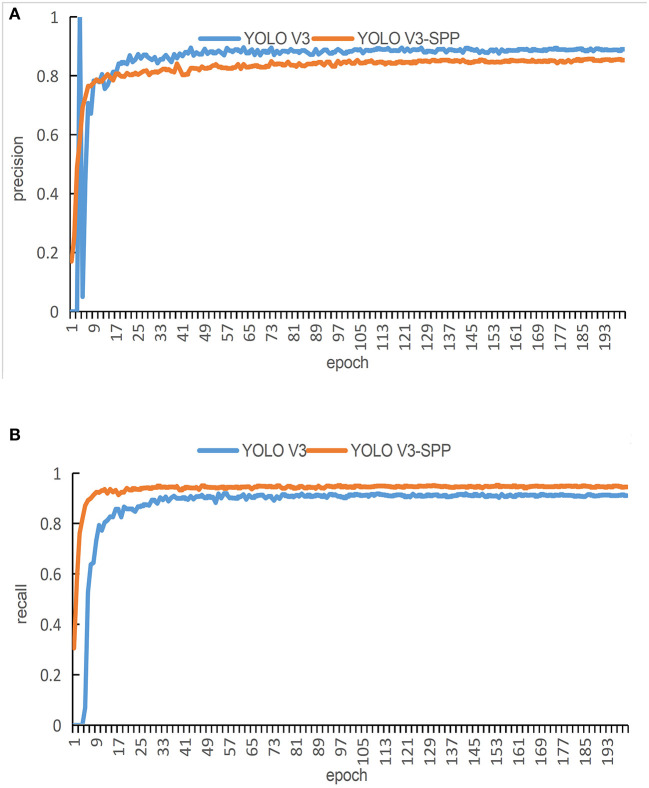
The training results of the detection algorithm by using the original images. **(A)** A comparison of the original and improved YOLOv3 in terms of precision for detection. **(B)** A comparison of the original and improved YOLOv3 in terms of recall for detection.

In order to evaluate the performance of the proposed network in terms of detection accuracy, we train the original YOLOv3 network and the YOLOv3-SPP network by using the original images. The precision and recall of both techniques are compared in Figure 7. Figure 7A presents the comparison of precision and Figure 7B presents the comparison of recall of both algorithms.

It is evident from Figure 7A that the red curve converges faster than the blue curve. In addition, the blue curve shows huge fluctuations at the beginning epochs. These fluctuations continue for around 100 epochs. After 100 epochs in the training process, both curves reach a relatively stable state. The curve at the final epochs shows that the blue curve maintains an interval of about 2% from the red curve, i.e., the blue curve is more precise than the red curve. Therefore, in terms of the detection, the original YOLOv3 performs slightly better than the proposed YOLOv3-SPP.

It is evident form Figure 7B that the red curve converges faster as compared with the blue curve. There is an interval of about 3% between the two curves after reaching a steady state. During the entire training process of 200 epochs, the precision of the red curve is always greater than the blue curve. In terms of the detection, the proposed YOLOv3-SPP performs better than the original YOLOv3.

In order to further verify the detection accuracy of the original and the proposed YOLOv3, we present the recognition results of the test_pv in Table 1. There are 108 images with 1,532 spots of grape leaf black rot in the test_pv set. The results show that the proposed YOLOv3-SPP successfully identifies 1,427 spots. Contrary, the original YOLOv3 only identifies a total of 1,283 spots. It is also noteworthy that the proposed YOLOv3-SPP misidentified 87 spots, which is 58 less than the misdetections of original YOLOv3. The number of FN of YOLOv3-SPP was 105, which was 154 less than that of YOLOv3. The proposed YOLOv3-SPP achieves the detection accuracy of 94.25% and a recall of 93.15%. These results are 4 and 9% higher than the original YOLOv3, respectively.

**Table 1:** The detection results of the test_pv before and after the improvement of YOLOv3 network.

**Original image of the test_pv with 1,532 objectives**	**TP**	**FP**	**FN**	**Precision**	**Recall**
YOLOv3	1,283	145	259	89.85%	83.75%
YOLOv3-SPP	1,427	87	105	94.25%	93.15%

**Figure 8:**
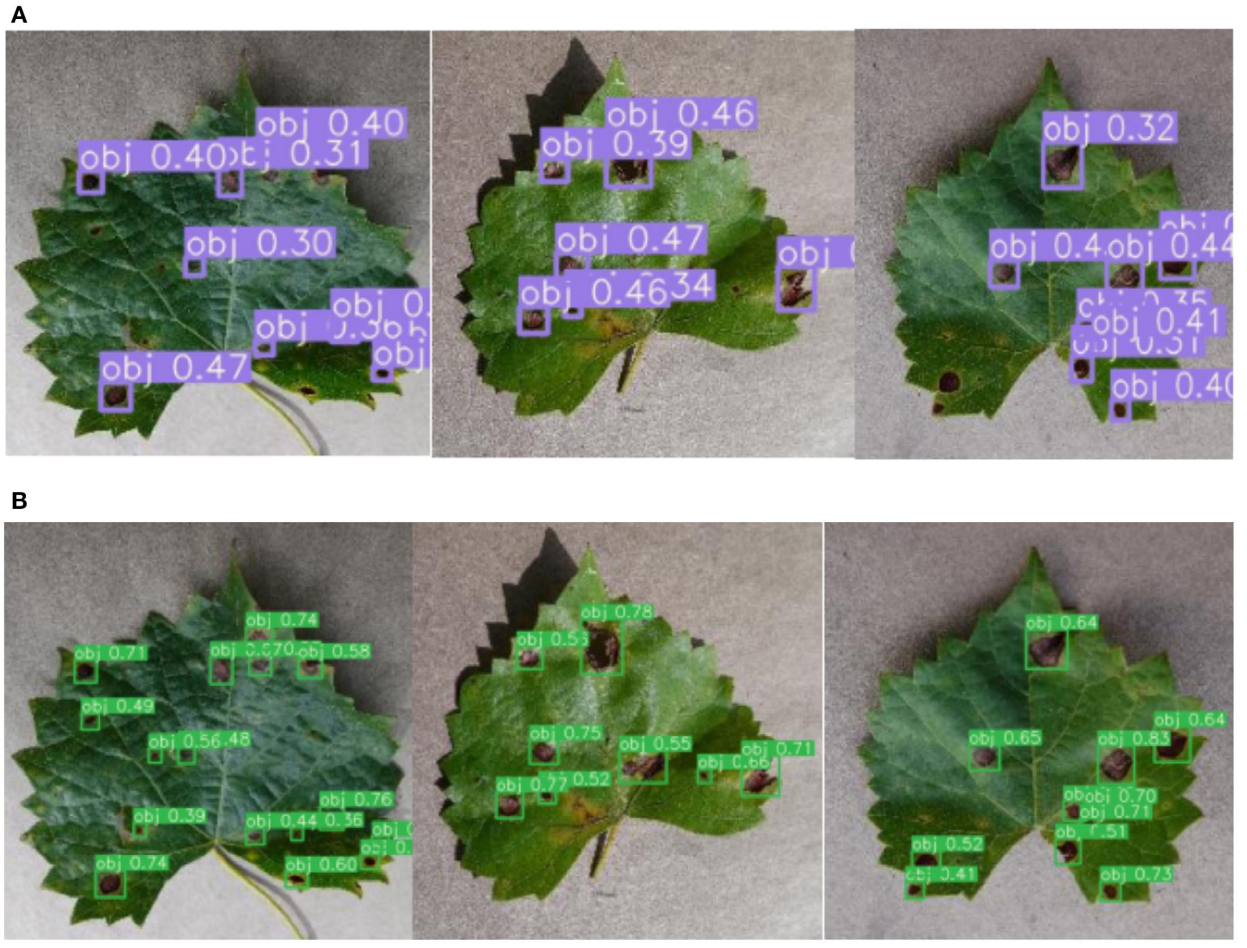
The comparison of detection results before and after the improvement of YOLOv3 network. **(A)** The detection results of the original YOLOv3 for original images. **(B)** The detection results of the improved YOLOv3-SPP for original images.

Figure 8 shows the detection results of the original and the improved algorithm. Figure 8A presents the recognition results of the original YOLOv3 and Figure 8B presents the recognition results of the improved YOLO V3-SPP. It is evident from Figure 8 that the recognition results of the improved algorithm are significantly better than the original algorithm. It is noteworthy that the targets missed by the original network are also recognized by the improved network. This shows that the addition of SPP module and the replacement of the loss function in the improved algorithm improve the recall of the detected targets effectively. Although the accuracy of the improved network is slightly lower than the original network. This indicates that the overall performance of the proposed YOLOv3-SPP algorithm is higher than that of the original YOLO V3, and the results of the improved method for grapevine leaf black rot detection are satisfactory.

### Comparison of Different Super-Resolution Algorithms for Image Enhancement

In this work, the BL is used to enhance the images. The enhanced images are then used for target detection. In this work, before we select the BL as a choice for this work, we compare the two other different super-resolution methods, i.e., the nearest interpolation and the enhanced deep residual networks (EDSR) (Lim et al., 2017) with BL. The nearest interpolation is a traditional and simplest interpolation method which is used to enhance the image by directly copying the values of neighboring pixels. On the other hand, EDSR has the ability to handle multiple scaling factors (scales) of super-resolution simultaneously in a single network. The training process of EDSR models using multiple scales significantly improve the performance. However, EDSR-type architectures require bicubic interpolation. This interpolation technique is computationally expensive and needs more storage space as well.

**Figure 9:**
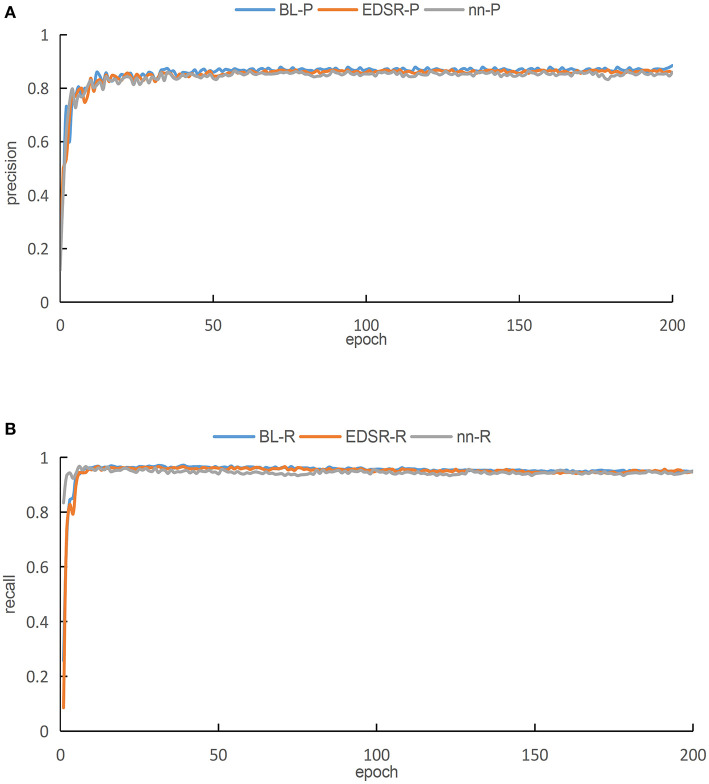
The training results of YOLOv3-SPP network after image enhancement by using different super-resolution algorithms. **(A)** A comparison of accuracies for different super resolutions. **(B)** A comparison of recall for different super resolutions.

In this work, we enhance the original images in the training dataset comprising 1,072 images by using all the three aforementioned super-resolution methods. These images are input into the proposed YOLOv3-SPP network for training. The training results are shown in Figure 9. Please note that Figure 9A presents a graph comparing the accuracies of different super-resolutions. As presented in Figure 9, the blue curve represents the result of BL, the red curve represents the result of EDSR, and the green curve represents the results of nearest interpolation.

As presented in Figure 9A, it is evident that when the images of the three different interpolation methods are fed into the proposed YOLOv3-SPP network separately, after 50 iterations, the precisions of all the networks tend to be stable. After 50 iterations, the precision of all three training methods fluctuates minutely, but the overall trend is stable. The comparison shows that the blue curve is better than the other two curves after achieving steady state. On the other hand, the green curve has a lower precision than the other two algorithms after becoming steady state. Therefore, we conclude that the BL technique has the highest accuracy rate P as compared with the other two super-resolution enhancement methods under the same parameters. As presented in Figure 9B, when the images of the three different interpolation methods are fed into the YOLOv3-SPP network separately, the resulting recalls approach 1 after 20 iterations approximately.

Please note that the red and blue curves are almost equal, however, the green curve is slightly below the other two curves. After processing the original image by using the three super-resolution image enhancement methods, the recalls approach 1, which are all higher than directly training the network using the original images. This indicates that the super-resolution enhancement of the images before using it as an input of the CNN for detection is better than using the original images directly. The evaluation metrics, i.e., precision and recall, make it evident that the network performs best when the image is enhanced using the BL technique as compared to the nearest interpolation. As compared to the EDSR method, the result of image enhancement performed using BL method has higher precision. and almost equal recall. However, the BL method has no complex residual convolution and is relatively less computationally intensive. We enhance 108 images in tese_pvaccording to the three aforementioned methods and then use the resultant images to perform detections using the proposed YOLOv3-SPP network. The corresponding results are shown in Table 2. Please note that the BL method has the highest recognition precision for the spots and the recall rate is only 0.13% lower than the EDSR method. The results of simulations performed using test_pv show that all the image enhancement methods perform well, however, considering the overall performance using the evaluation metrics shows that the BL method is superior.

**Table 2:** The evaluation of test_pv by using different super-resolution methods.

**Evaluation indicators**	**BL**	**Nearest interpolation**	**EDSR**
TP	1,448	1,430	1,450
FP	65	78	70
FN	84	102	82
Precision	95.79%	94.83%	95.39%
Recall	94.52%	93.34%	94.65%

### Comparison of the Detection Effect of Super-Resolution Image and Original Image Under the Improved Network

**Figure 10:**
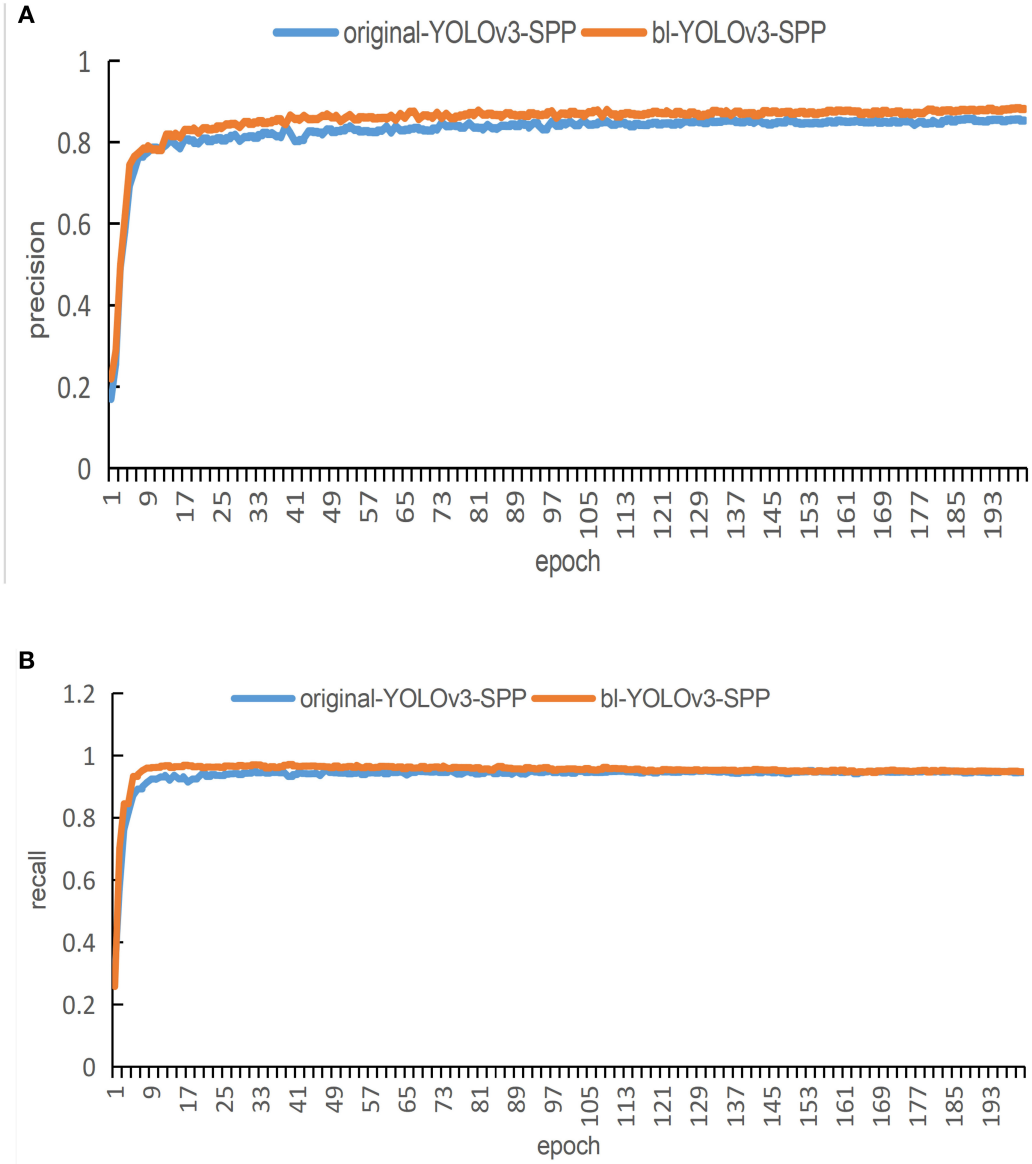
The training results of the network before and after image enhancement. **(A)** A comparison of the detection accuracy under super-resolution image and original image. **(B)** A comparison of the detection recalls under the super-resolution image and the original image.

The training results of the proposed YOLO V3-SPP network by using the original and the enhanced images are presented in Figure 10. The blue and red curves in Figure 10 represent the training results for original and improved images, respectively.

Figure 10A shows the comparison of detection accuracy under the super-resolution image and the original image. The results show that the red curve and the blue curve almost converge simultaneously, however, the red curve after smoothing is close to 0.9. The blue curve lags behind the red by about 1.2%. Both curves have occasional fluctuations during the process of convergence. After reaching 140 epochs, both curves smooth out. After enhancing the images using super-resolution, the accuracy of the network is higher than the accuracy obtained by using the unenhanced images.

Figure 10B shows the comparison of detection accuracy under the super-resolution images and the original images. The results show that the two curves rapidly stabilize at the beginning of the training process. Please note that the blue curve is lower than the red curve for first 100 epochs, but after 100 iterations both curves are relatively stable. The figure shows that both curves exhibit good recall. This indicates that after enhancing the images using super-resolution, the performance of the network reflects that its recall is not lower than that of the unenhanced images.

The test result of the proposed YOLOv3-SPP trained with the original and the enhanced images in test_pv are shown in Table 3. The YOLOv3-SPP trained using the enhanced images correctly identifies a total of 1,448 spots, that is 21 more spots than the YOLOv3-SPP trained using the original images. In addition, the number of false identifications is reduced by 22, and the number of miss identifications decreased by 21. In terms of precision and recognition rate, the proposed YOLOv3-SPP network trained on the enhanced images improved the performance by 1.54 and 1.37%, respectively.

**Table 3:** The detection results on test_pv set.

**YOLOv3-SPP**	**Objectives**	**TP**	**FP**	**FN**	**Precision**	**Recall**
Original image of test_pv	1,532	1,427	87	105	94.25%	93.15%
Super Resolution of test_pv	1,532	1,448	65	84	95.79%	94.52%

**Figure 11:**
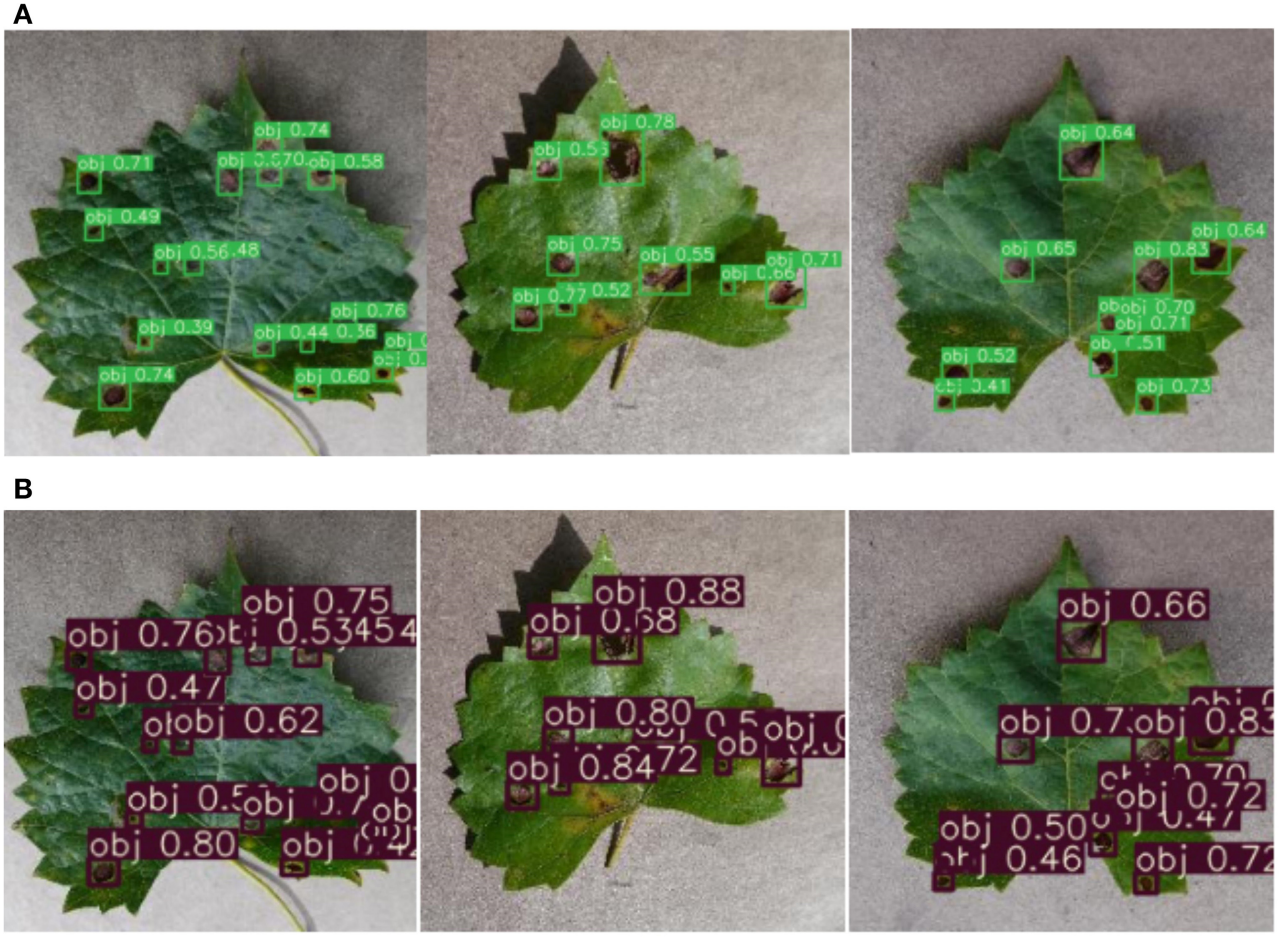
The detection results before and after the data is enhanced for test_pv. **(A)** The detection results of YOLOv3-SPP trained on the unenhanced images. **(B)** The detection results of YOLOv3-SPP trained on the enhanced images.

Figure 11 shows the recognition results before and after the images are enhanced. Figure 11A shows the detection results of YOLOv3-SPP trained using the unenhanced images. Figure 11B presents the detection results of YOLOv3-SPP trained using the enhanced images. As presented in Figure 11, the proposed YOLOv3-SPP trained using the enhanced images to detect the black rot of grape leaves has significantly better results than the recognition results of the network trained using the unenhanced images. This indicates that the introduction of super-resolution images for grape leaf black rot detection improves the accuracy of the target detectors without reducing the recall rate.

### The Detection Effect in the Orchard Environment

**Figure 12:**
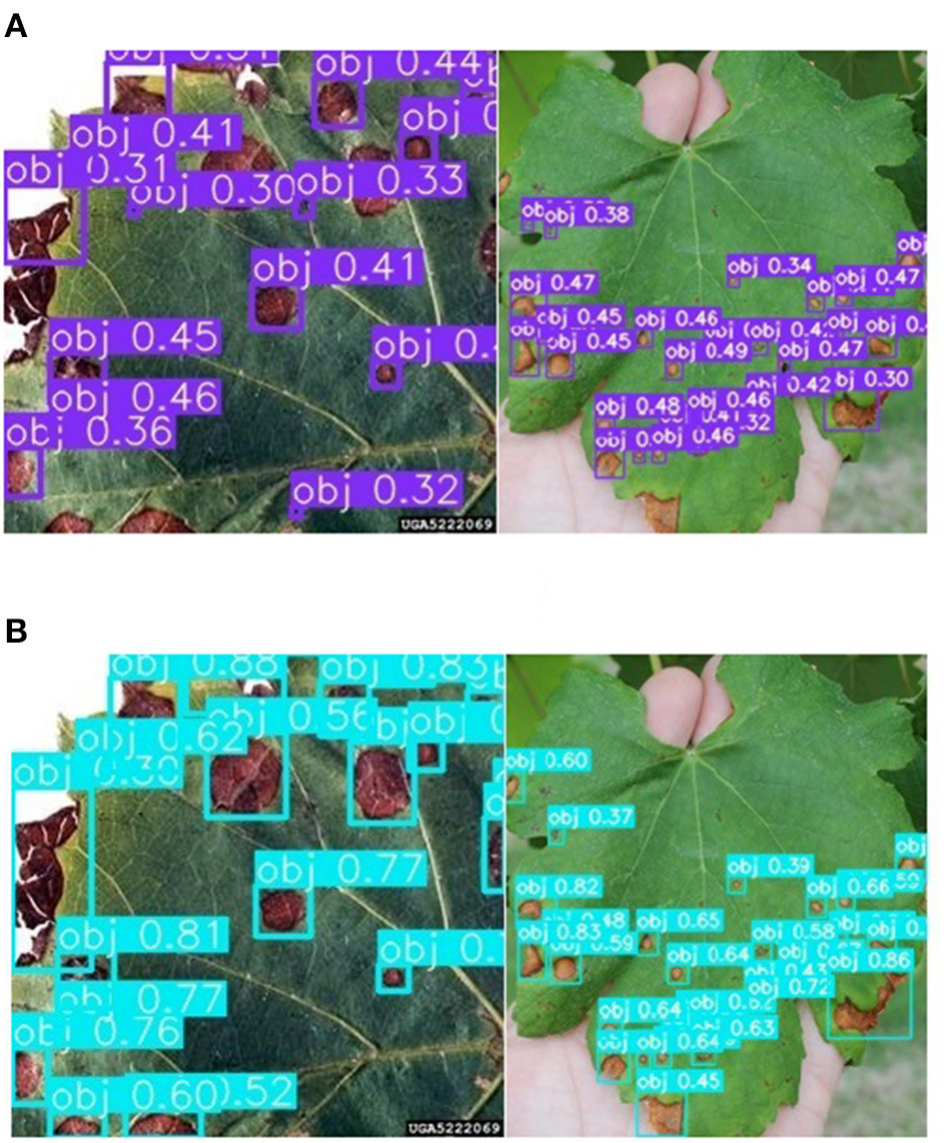
The detection results before and after the data is enhanced for test_orchard. **(A)** The detection results of YOLOv3-SPP trained on the unenhanced images. **(B)** The detection results of YOLOv3-SPP trained on the enhanced images.

The method of super-resolution image enhancement and deep learning can improve the detection effect of grape leaf black rot, which has been proved in the test_pv data set. An additional test set, test_orchard, was used to test the effectiveness of the proposed method in the orchard environment. There are 108 images of grape leaves with 1,275 spots of grape leaf black rot from different orchard environments. The results of spot identification by YOLOv3-SPP were shown in Figure 12. It can be seen from Figure 12 that not only the detection precision of the spot was improved, but also the recall was improved after enhancing the images using super-resolution. Some undetected spots in the original image were identified on the enhanced images. The statistical data of identification results of the test_orchard was shown in Table 4. There are 1,275 spots in the test set, and 1,028 spots were recognized after inputting the original images into the YOLOv3-SPP, of which 186 spots were misidentified. The number of unrecognized spots was 247. The precision of disease spot detection was 84.68% and the recall was 80.63% for the original images of test_orchard. However, a total of 1,049 disease spots were identified for the images after enhancement by super-resolution, of which 161 spots were misidentified. The number of unrecognized spots was 247. The precision of disease spot detection was 86.69% and the recall was 82.27% for the super-resolution images of test_orchard. The number of misidentified and unrecognized spots decreased, and the precision and recall increased 2.01 and 1.64%, respectively after the images were enhanced by super-resolution.

**Table 4:** The detection results on test_orchard set.

**YOLOv3-SPP**	**Objective**	**TP**	**FP**	**FN**	**Precision**	**Recall**
Original image test_orchard set	1,275	1,028	186	247	84.68%	80.63%
Super-resolution test_orchard set	1,275	1,049	161	226	86.69%	82.27%

**Figure 13:**
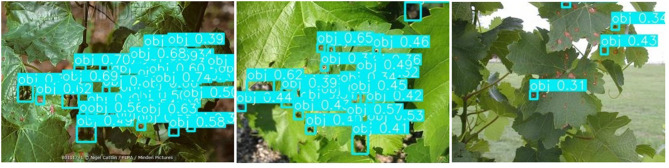
Detection results of multi-leaf images.

The detection precision and recall of test_orchard were lower than that of test_pv, because the images of test_orchard are from orchards, while the images of test_pv from Plant Village, which photograph indoors. The environment of orchards is complex compared to the indoor. The images of the test_orchard were classified into single-leaf and multi-leaf based on the number of grape leaves in the images, to compare the influence of different image acquisition ways on the detection effect. The detection results of the multi-leaf images were shown in Figure 13, and it can be seen that the leaf slits to be misrecognized as disease spots, which reduced the precision. The statistical results of the detection were shown in Table 5 for the two category images. There are 701 spots in single-leaf images totally, 601 spots were detected, 71 disease spots were wrong identified and 91 spots were missed. The detection precision was 89.57% and the recall was 87.02% for the single-leaf images. A total of 574 disease spots in multi-leaf images and 135 spots were missed detection. Among the identified 439 spots, 90 spots were misidentified. The detection precision was 82.99% and the recall was 76.48% for the multi-leaf images. The detection precision and recall of single-leaf images are 6.76 and 10.54% higher than that of multi-leaf images.

**Table 5:** The statistical results of the detection of single-leaf and multi-leaf images for test_orchard.

**BL-YOLOv3-SPP**	**Objective**	**TP**	**FP**	**FN**	**Precision**	**Recall**
Single-leaf	701	610	71	91	89.57%	87.02%
Multi-leaf	574	439	90	135	82.99%	76.48%

This shows that the acquisition of single grape leaf image is more conductive to detection.

**Figure 14:**
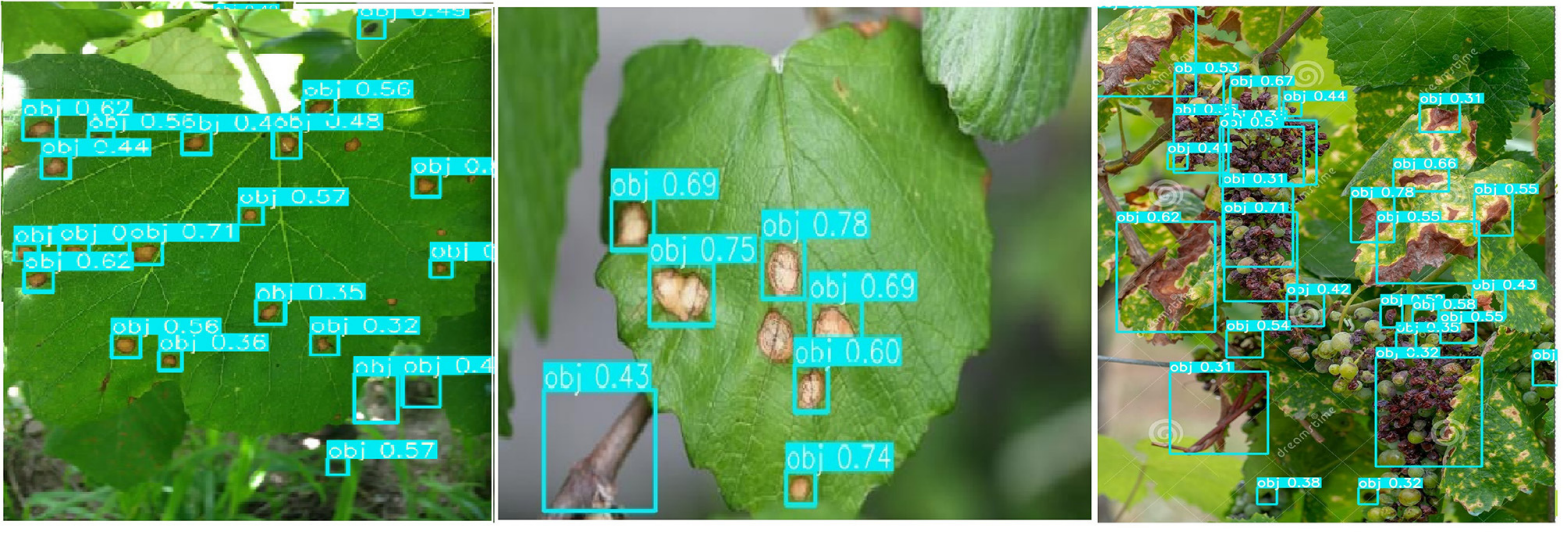
Detection results of grapevine leaf black rot with complex background.

The background of images also affected the detection effect, so the set of test_orchard was divided into two subsets, simple background images and complex background images based on the status of the background. The images are considered to be complex background, which concludes fruits, branches, and soil except for grape leaf, otherwise, they are considered simple background. The detection results of the complex background images were shown in Figure 14, and it can be seen that the rotten fruits, gray branches, and soil to be misrecognized as disease spots, which also reduced the precision. The statistical results of the detection were shown in Table 6 for the test_orchard images in the different backgrounds. There are 712 spots in simple background images totally, 664 spots were detected, 42 disease spots were wrong identified and 48 spots were missed. The detection precision was 94.05% and the recall was 93.26% for the simple background images, which was close to the detection effect of test_pv. A total of 563 disease spots in complex background images and 178 spots were missed detection. Among the identified 386 spots, 119 spots were misidentified. The detection precision was 76.39% and the recall was 68.38% for the complex background images. The detection precision and recall of simple background images are 17.66 and 24.9% higher than that of complex background images. It can also be seen from the data in Table 6, that the influence of background in the image is greater than that of multiple leaves in the image for the grape leaf black rot.

**Table 6:** The statistical results of the detection of different background for test_orchard.

**BL-YOLOv3-SPP**	**Objective**	**TP**	**FP**	**FN**	**Precision**	**Recall**
Simple background	712	664	42	48	94.05%	93.26%
Complicated background	563	385	119	178	76.39%	68.38%

The method proposed in this work can be used for the detection of grape leaf black rot in the natural environment through the test and analysis of the images collected from orchards, and the detection effect is satisfactory especially in the case of the simple background. At the same time, the analysis results also provide a reference for the field image acquisition, that is, to avoid other objects appearing in the image except for grape leaf.

## Conclusions

In this work, we propose an improved YOLOv3-SPP model for the detection of black rot of grape leaves. This method replaces the loss function in the original YOLOv3 with GIOU. In addition, we also add the SPP module. We enhance the training images of YOLOv3-SPP by using BL super-resolution method. Two test sets from Plant Village dataset and orchards are performed on the model. The results show that the YOLOv3-SPP network performs better for grape leaf black rot detection and has a precision of 95.79% and recall of 94.52% for the test set from Plant Village. For the orchards test set, the precision is 86.69% and the recall is 82.27%, it also has better performs than the original images of the test set. In addition, the precision and recall are improved to 94.05 and 93.26% for those images without fruits, branches, and soil in the background. Moreover, the image enhancement of the training set using the BL method improves the results in terms of precision and recall. The current method requires image enhancement and then trains the deep learning network. In future work, we will attempt to combine these steps.

## Data Availability Statement

The original contributions presented in the study are included in the article/Supplementary Material, further inquiries can be directed to the corresponding author.

## Author Contributions

JZ, MC, and HY: conceived the idea and proposed the method. JZ and QW: contributed to the preparation of equipment and acquisition of data, and wrote the code and tested the method. JZ, QW, and MC: validation results. JZ and HY: wrote the paper. JZ, HY, and ZC: revised the paper. All authors read and approved the final manuscript.

## Conflict of Interest

The authors declare that the research was conducted in the absence of any commercial or financial relationships that could be construed as a potential conflict of interest.
