# Supplementary figures and images for: Grape Leaf Black Rot Detection Based on Super-Resolution Image Enhancement and Deep Learning (part 2 of 6)
Source: Front Plant Sci. 2021 Jun 29;12:695749. doi: 10.3389/fpls.2021.695749 (PMC8277438; doi:10.3389/fpls.2021.695749)

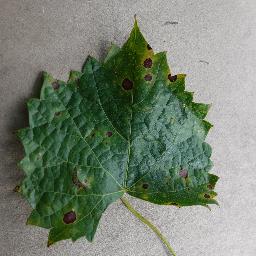

Supplement: Supplementary file 1 [file Data_Sheet_1.ZIP › training data/1098.jpg]

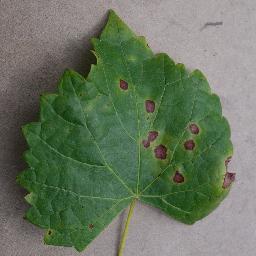

Supplement: Supplementary file 1 [file Data_Sheet_1.ZIP › training data/1099.jpg]

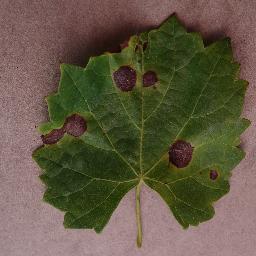

Supplement: Supplementary file 1 [file Data_Sheet_1.ZIP › training data/110.JPG]

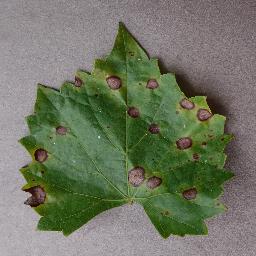

Supplement: Supplementary file 1 [file Data_Sheet_1.ZIP › training data/1100.jpg]

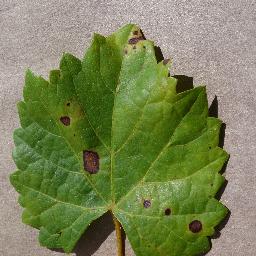

Supplement: Supplementary file 1 [file Data_Sheet_1.ZIP › training data/1101.jpg]

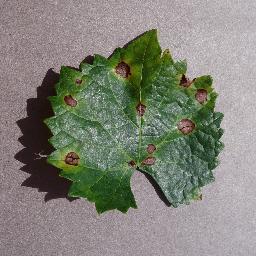

Supplement: Supplementary file 1 [file Data_Sheet_1.ZIP › training data/1102.jpg]

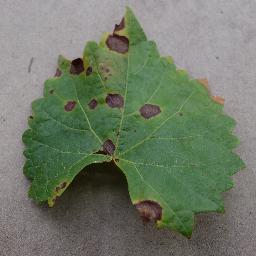

Supplement: Supplementary file 1 [file Data_Sheet_1.ZIP › training data/1103.jpg]

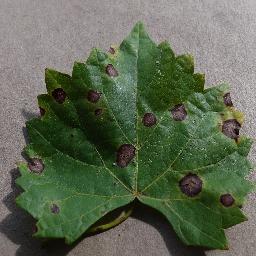

Supplement: Supplementary file 1 [file Data_Sheet_1.ZIP › training data/1104.jpg]

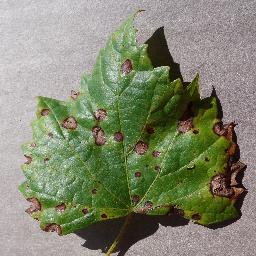

Supplement: Supplementary file 1 [file Data_Sheet_1.ZIP › training data/1105.jpg]

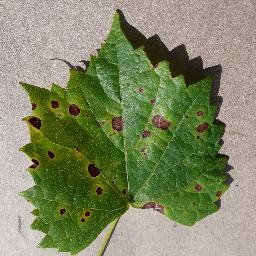

Supplement: Supplementary file 1 [file Data_Sheet_1.ZIP › training data/1106.jpg]

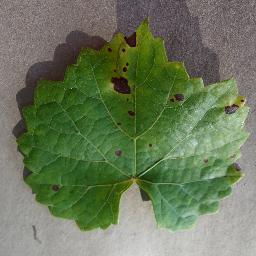

Supplement: Supplementary file 1 [file Data_Sheet_1.ZIP › training data/1107.jpg]

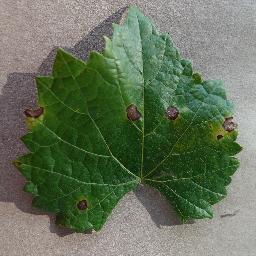

Supplement: Supplementary file 1 [file Data_Sheet_1.ZIP › training data/1108.jpg]

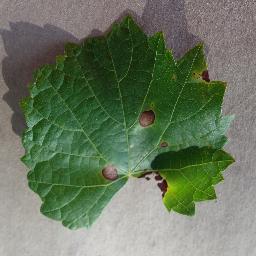

Supplement: Supplementary file 1 [file Data_Sheet_1.ZIP › training data/1109.jpg]

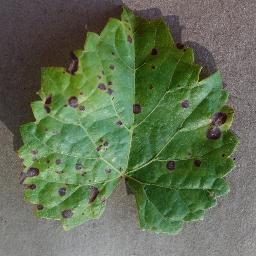

Supplement: Supplementary file 1 [file Data_Sheet_1.ZIP › training data/111.JPG]

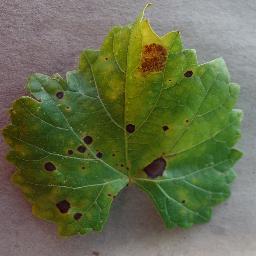

Supplement: Supplementary file 1 [file Data_Sheet_1.ZIP › training data/1110.jpg]

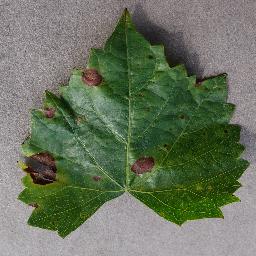

Supplement: Supplementary file 1 [file Data_Sheet_1.ZIP › training data/1111.jpg]

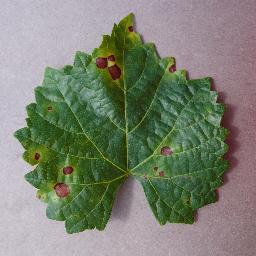

Supplement: Supplementary file 1 [file Data_Sheet_1.ZIP › training data/1112.jpg]

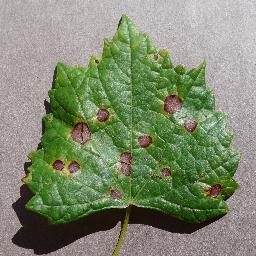

Supplement: Supplementary file 1 [file Data_Sheet_1.ZIP › training data/1113.jpg]

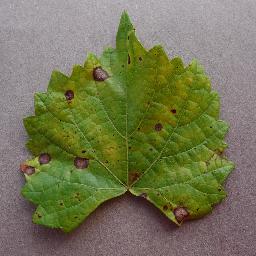

Supplement: Supplementary file 1 [file Data_Sheet_1.ZIP › training data/1114.jpg]

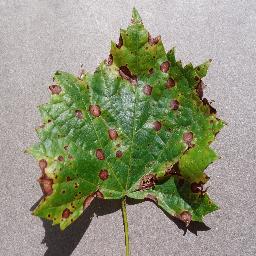

Supplement: Supplementary file 1 [file Data_Sheet_1.ZIP › training data/1115.jpg]

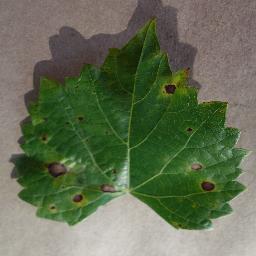

Supplement: Supplementary file 1 [file Data_Sheet_1.ZIP › training data/1116.jpg]

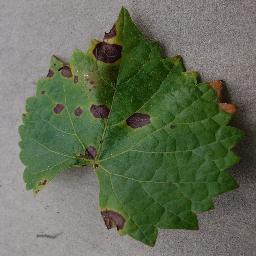

Supplement: Supplementary file 1 [file Data_Sheet_1.ZIP › training data/1117.jpg]

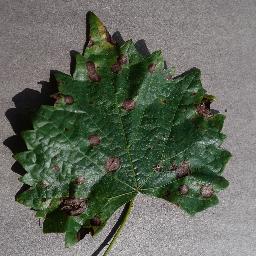

Supplement: Supplementary file 1 [file Data_Sheet_1.ZIP › training data/1118.jpg]

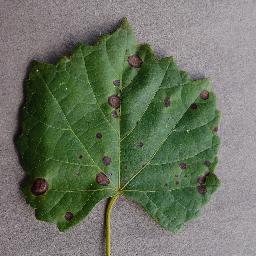

Supplement: Supplementary file 1 [file Data_Sheet_1.ZIP › training data/1119.jpg]

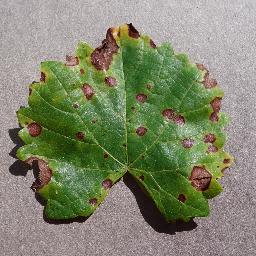

Supplement: Supplementary file 1 [file Data_Sheet_1.ZIP › training data/112.JPG]

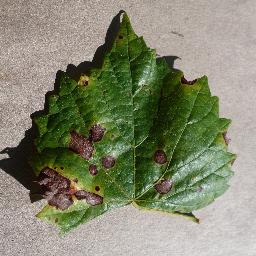

Supplement: Supplementary file 1 [file Data_Sheet_1.ZIP › training data/1120.jpg]

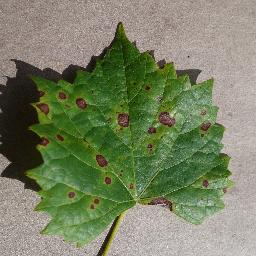

Supplement: Supplementary file 1 [file Data_Sheet_1.ZIP › training data/1121.jpg]

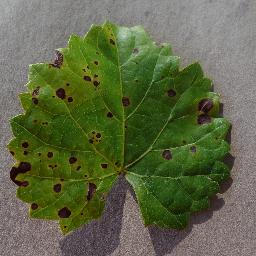

Supplement: Supplementary file 1 [file Data_Sheet_1.ZIP › training data/1122.jpg]

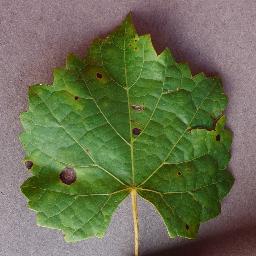

Supplement: Supplementary file 1 [file Data_Sheet_1.ZIP › training data/1123.jpg]

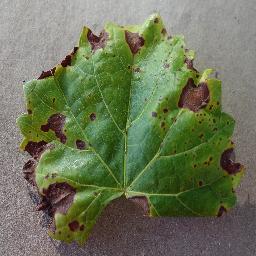

Supplement: Supplementary file 1 [file Data_Sheet_1.ZIP › training data/1124.jpg]

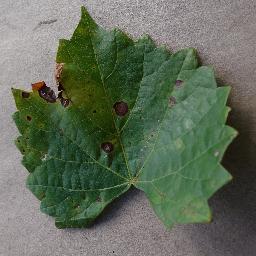

Supplement: Supplementary file 1 [file Data_Sheet_1.ZIP › training data/1125.jpg]

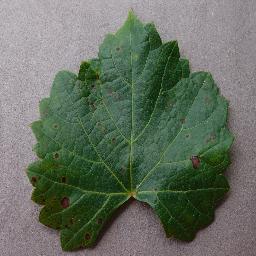

Supplement: Supplementary file 1 [file Data_Sheet_1.ZIP › training data/1126.jpg]

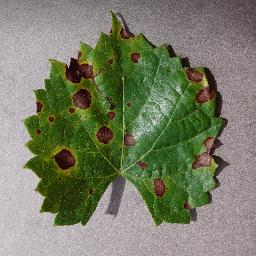

Supplement: Supplementary file 1 [file Data_Sheet_1.ZIP › training data/1127.jpg]

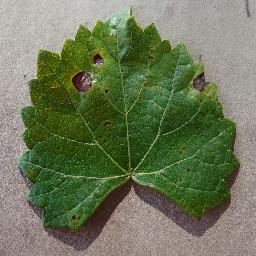

Supplement: Supplementary file 1 [file Data_Sheet_1.ZIP › training data/1128.jpg]

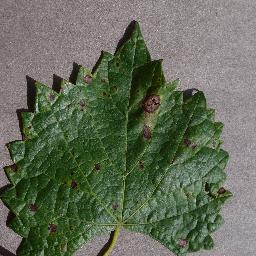

Supplement: Supplementary file 1 [file Data_Sheet_1.ZIP › training data/1129.jpg]

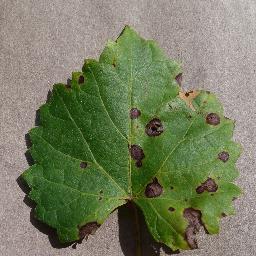

Supplement: Supplementary file 1 [file Data_Sheet_1.ZIP › training data/113.JPG]

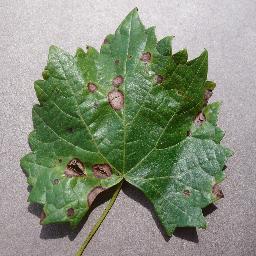

Supplement: Supplementary file 1 [file Data_Sheet_1.ZIP › training data/1130.jpg]

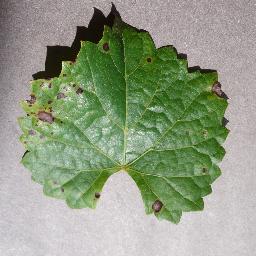

Supplement: Supplementary file 1 [file Data_Sheet_1.ZIP › training data/1131.jpg]

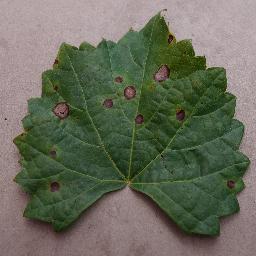

Supplement: Supplementary file 1 [file Data_Sheet_1.ZIP › training data/1132.jpg]

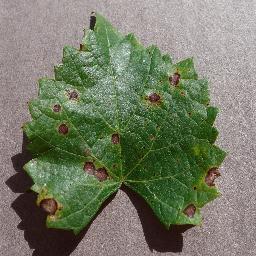

Supplement: Supplementary file 1 [file Data_Sheet_1.ZIP › training data/1133.jpg]

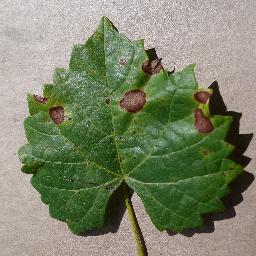

Supplement: Supplementary file 1 [file Data_Sheet_1.ZIP › training data/1134.jpg]

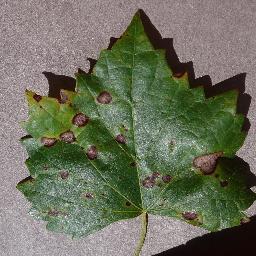

Supplement: Supplementary file 1 [file Data_Sheet_1.ZIP › training data/1135.jpg]

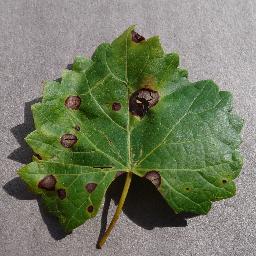

Supplement: Supplementary file 1 [file Data_Sheet_1.ZIP › training data/1136.jpg]

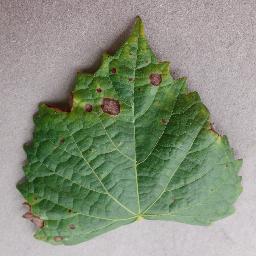

Supplement: Supplementary file 1 [file Data_Sheet_1.ZIP › training data/1137.jpg]

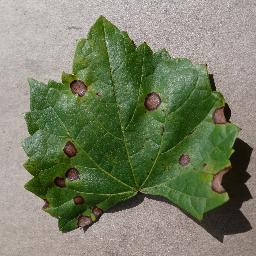

Supplement: Supplementary file 1 [file Data_Sheet_1.ZIP › training data/1138.jpg]

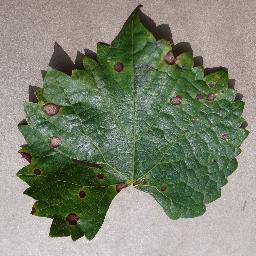

Supplement: Supplementary file 1 [file Data_Sheet_1.ZIP › training data/1139.jpg]

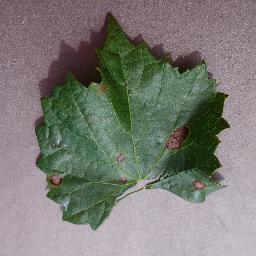

Supplement: Supplementary file 1 [file Data_Sheet_1.ZIP › training data/114.JPG]

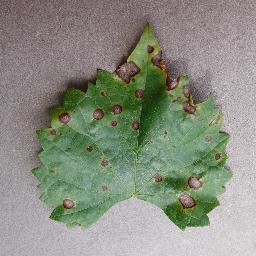

Supplement: Supplementary file 1 [file Data_Sheet_1.ZIP › training data/1140.jpg]

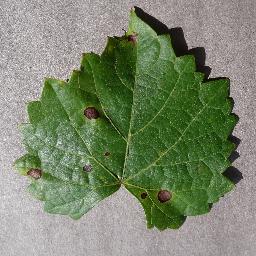

Supplement: Supplementary file 1 [file Data_Sheet_1.ZIP › training data/1141.jpg]

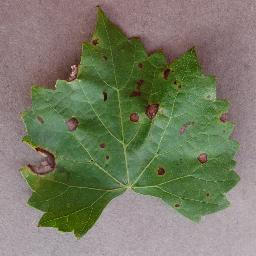

Supplement: Supplementary file 1 [file Data_Sheet_1.ZIP › training data/1142.jpg]

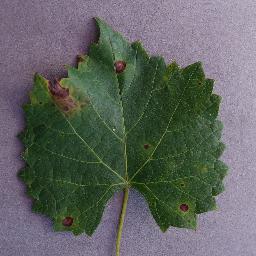

Supplement: Supplementary file 1 [file Data_Sheet_1.ZIP › training data/1143.jpg]

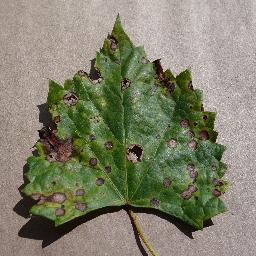

Supplement: Supplementary file 1 [file Data_Sheet_1.ZIP › training data/1144.jpg]

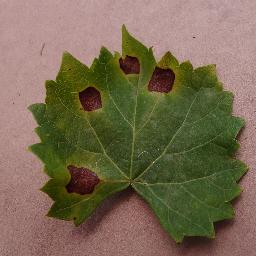

Supplement: Supplementary file 1 [file Data_Sheet_1.ZIP › training data/1145.jpg]

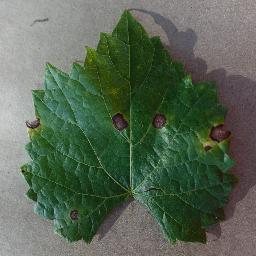

Supplement: Supplementary file 1 [file Data_Sheet_1.ZIP › training data/1147.jpg]

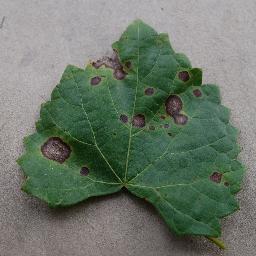

Supplement: Supplementary file 1 [file Data_Sheet_1.ZIP › training data/1148.jpg]

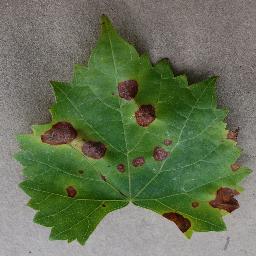

Supplement: Supplementary file 1 [file Data_Sheet_1.ZIP › training data/1149.jpg]

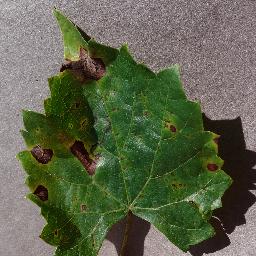

Supplement: Supplementary file 1 [file Data_Sheet_1.ZIP › training data/115.JPG]

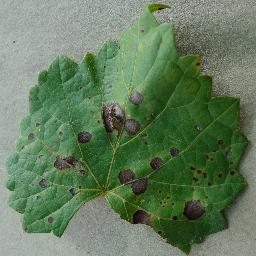

Supplement: Supplementary file 1 [file Data_Sheet_1.ZIP › training data/1150.jpg]

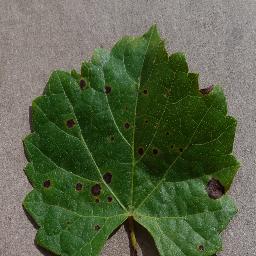

Supplement: Supplementary file 1 [file Data_Sheet_1.ZIP › training data/1151.jpg]

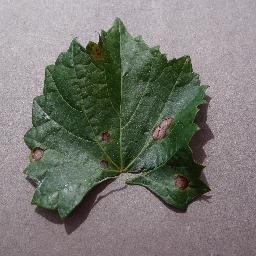

Supplement: Supplementary file 1 [file Data_Sheet_1.ZIP › training data/1153.jpg]

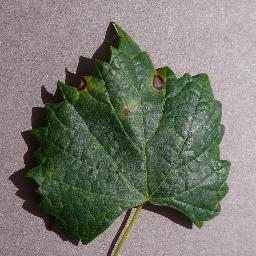

Supplement: Supplementary file 1 [file Data_Sheet_1.ZIP › training data/1154.jpg]

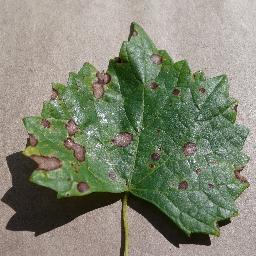

Supplement: Supplementary file 1 [file Data_Sheet_1.ZIP › training data/1155.jpg]

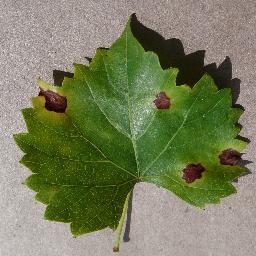

Supplement: Supplementary file 1 [file Data_Sheet_1.ZIP › training data/1157.jpg]

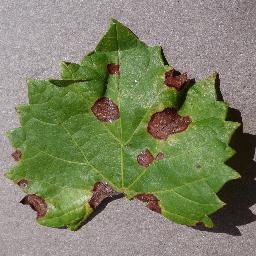

Supplement: Supplementary file 1 [file Data_Sheet_1.ZIP › training data/1158.jpg]

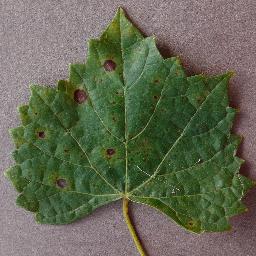

Supplement: Supplementary file 1 [file Data_Sheet_1.ZIP › training data/1159.jpg]

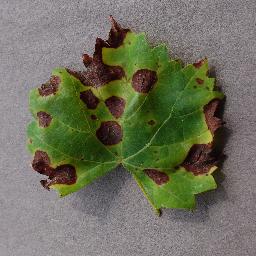

Supplement: Supplementary file 1 [file Data_Sheet_1.ZIP › training data/116.JPG]

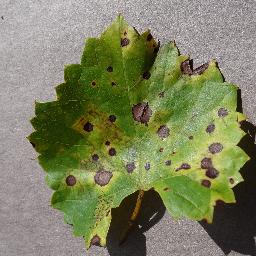

Supplement: Supplementary file 1 [file Data_Sheet_1.ZIP › training data/1160.jpg]

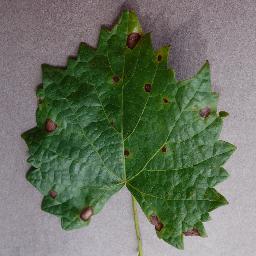

Supplement: Supplementary file 1 [file Data_Sheet_1.ZIP › training data/1161.jpg]

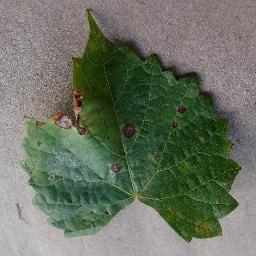

Supplement: Supplementary file 1 [file Data_Sheet_1.ZIP › training data/1162.jpg]

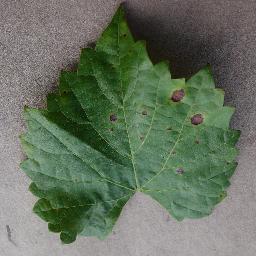

Supplement: Supplementary file 1 [file Data_Sheet_1.ZIP › training data/1163.jpg]

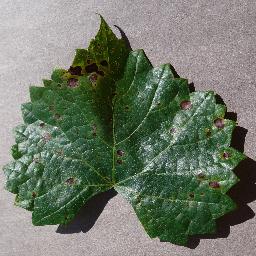

Supplement: Supplementary file 1 [file Data_Sheet_1.ZIP › training data/1164.jpg]

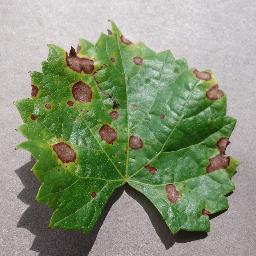

Supplement: Supplementary file 1 [file Data_Sheet_1.ZIP › training data/1165.jpg]

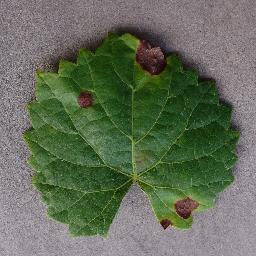

Supplement: Supplementary file 1 [file Data_Sheet_1.ZIP › training data/1166.jpg]

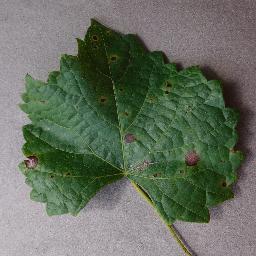

Supplement: Supplementary file 1 [file Data_Sheet_1.ZIP › training data/1167.jpg]

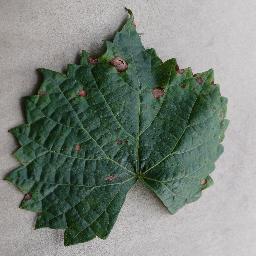

Supplement: Supplementary file 1 [file Data_Sheet_1.ZIP › training data/1168.jpg]

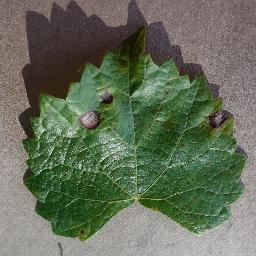

Supplement: Supplementary file 1 [file Data_Sheet_1.ZIP › training data/1169.jpg]

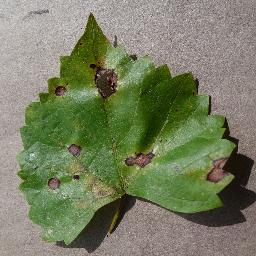

Supplement: Supplementary file 1 [file Data_Sheet_1.ZIP › training data/117.JPG]

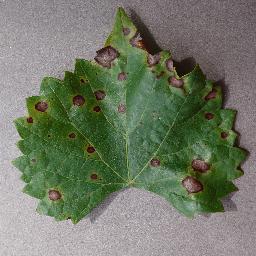

Supplement: Supplementary file 1 [file Data_Sheet_1.ZIP › training data/1170.jpg]

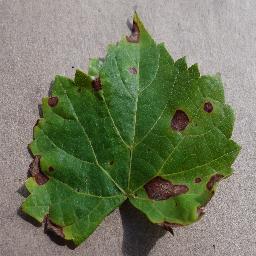

Supplement: Supplementary file 1 [file Data_Sheet_1.ZIP › training data/1171.jpg]

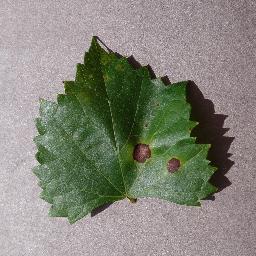

Supplement: Supplementary file 1 [file Data_Sheet_1.ZIP › training data/1172.jpg]

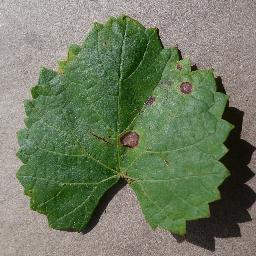

Supplement: Supplementary file 1 [file Data_Sheet_1.ZIP › training data/1173.jpg]

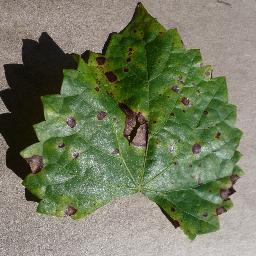

Supplement: Supplementary file 1 [file Data_Sheet_1.ZIP › training data/1174.jpg]

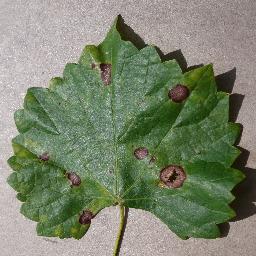

Supplement: Supplementary file 1 [file Data_Sheet_1.ZIP › training data/1175.jpg]

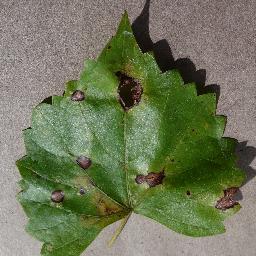

Supplement: Supplementary file 1 [file Data_Sheet_1.ZIP › training data/1176.jpg]

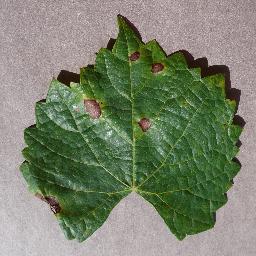

Supplement: Supplementary file 1 [file Data_Sheet_1.ZIP › training data/1177.jpg]

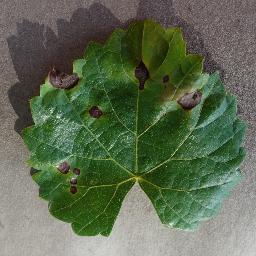

Supplement: Supplementary file 1 [file Data_Sheet_1.ZIP › training data/1178.jpg]

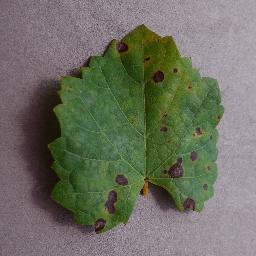

Supplement: Supplementary file 1 [file Data_Sheet_1.ZIP › training data/1179.jpg]

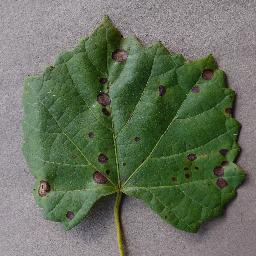

Supplement: Supplementary file 1 [file Data_Sheet_1.ZIP › training data/118.JPG]

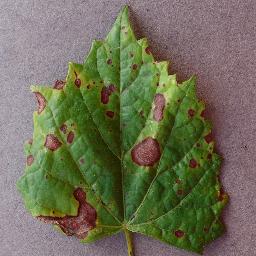

Supplement: Supplementary file 1 [file Data_Sheet_1.ZIP › training data/119.jpg]

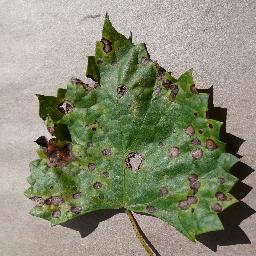

Supplement: Supplementary file 1 [file Data_Sheet_1.ZIP › training data/129.jpg]

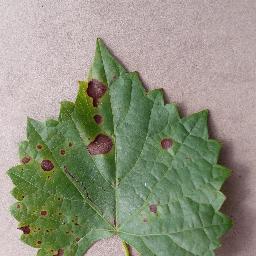

Supplement: Supplementary file 1 [file Data_Sheet_1.ZIP › training data/130.jpg]

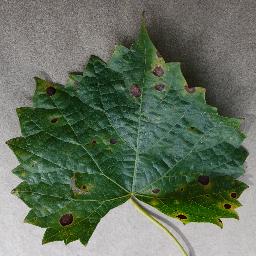

Supplement: Supplementary file 2 [file Data_Sheet_2.ZIP › test_pv/0.JPG]

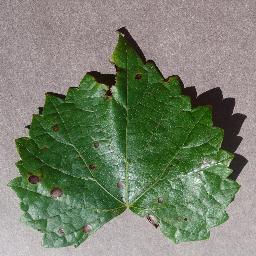

Supplement: Supplementary file 2 [file Data_Sheet_2.ZIP › test_pv/1.JPG]

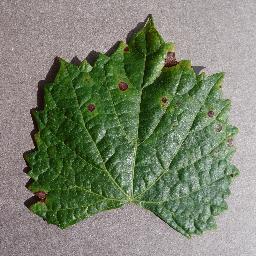

Supplement: Supplementary file 2 [file Data_Sheet_2.ZIP › test_pv/10.JPG]

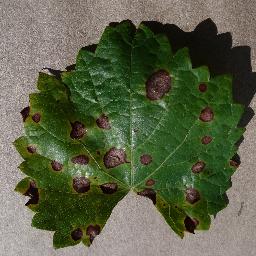

Supplement: Supplementary file 2 [file Data_Sheet_2.ZIP › test_pv/100.JPG]

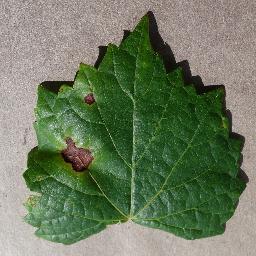

Supplement: Supplementary file 2 [file Data_Sheet_2.ZIP › test_pv/101.JPG]

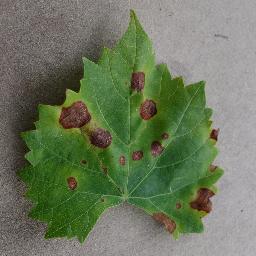

Supplement: Supplementary file 2 [file Data_Sheet_2.ZIP › test_pv/102.JPG]

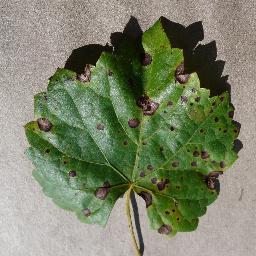

Supplement: Supplementary file 2 [file Data_Sheet_2.ZIP › test_pv/103.JPG]

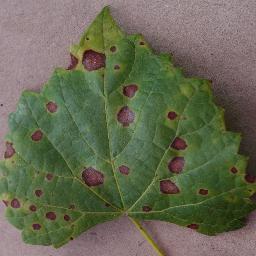

Supplement: Supplementary file 2 [file Data_Sheet_2.ZIP › test_pv/104.JPG]

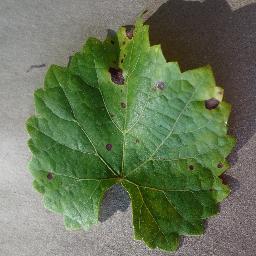

Supplement: Supplementary file 2 [file Data_Sheet_2.ZIP › test_pv/105.JPG]
