# Supplementary figures and images for: Grape Leaf Black Rot Detection Based on Super-Resolution Image Enhancement and Deep Learning (part 1 of 6)
Source: Front Plant Sci. 2021 Jun 29;12:695749. doi: 10.3389/fpls.2021.695749 (PMC8277438; doi:10.3389/fpls.2021.695749)

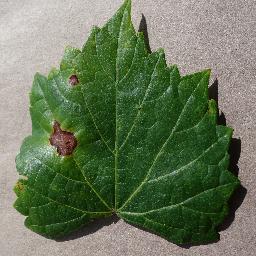

Supplement: Supplementary file 1 [file Data_Sheet_1.ZIP › training data/1000.jpg]

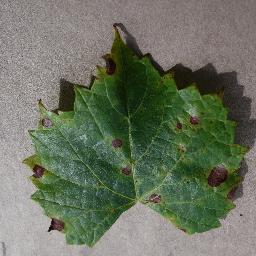

Supplement: Supplementary file 1 [file Data_Sheet_1.ZIP › training data/1001.jpg]

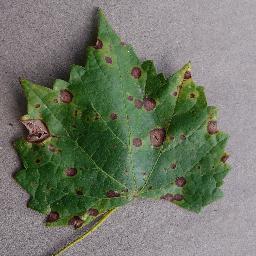

Supplement: Supplementary file 1 [file Data_Sheet_1.ZIP › training data/1002.jpg]

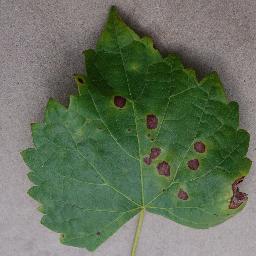

Supplement: Supplementary file 1 [file Data_Sheet_1.ZIP › training data/1003.jpg]

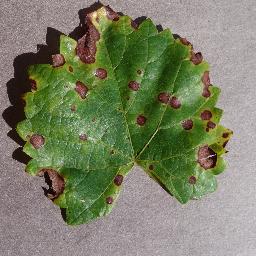

Supplement: Supplementary file 1 [file Data_Sheet_1.ZIP › training data/1004.jpg]

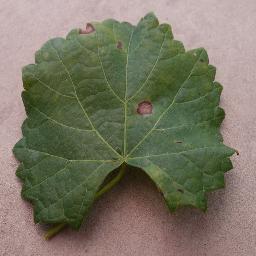

Supplement: Supplementary file 1 [file Data_Sheet_1.ZIP › training data/1005.jpg]

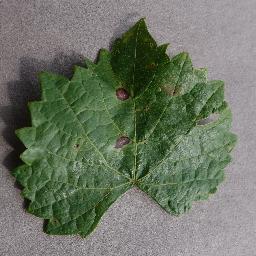

Supplement: Supplementary file 1 [file Data_Sheet_1.ZIP › training data/1006.jpg]

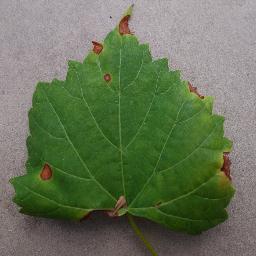

Supplement: Supplementary file 1 [file Data_Sheet_1.ZIP › training data/1007.jpg]

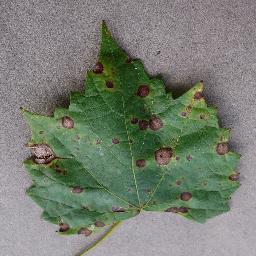

Supplement: Supplementary file 1 [file Data_Sheet_1.ZIP › training data/1008.jpg]

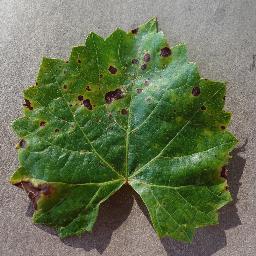

Supplement: Supplementary file 1 [file Data_Sheet_1.ZIP › training data/1009.jpg]

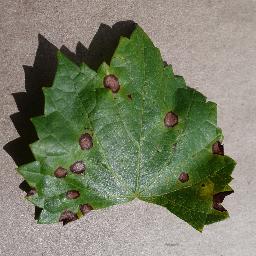

Supplement: Supplementary file 1 [file Data_Sheet_1.ZIP › training data/1010.jpg]

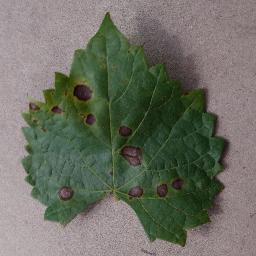

Supplement: Supplementary file 1 [file Data_Sheet_1.ZIP › training data/1011.jpg]

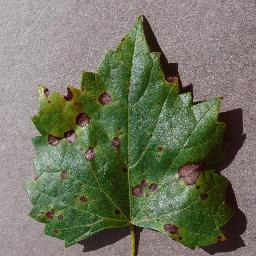

Supplement: Supplementary file 1 [file Data_Sheet_1.ZIP › training data/1012.jpg]

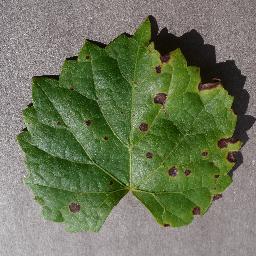

Supplement: Supplementary file 1 [file Data_Sheet_1.ZIP › training data/1013.jpg]

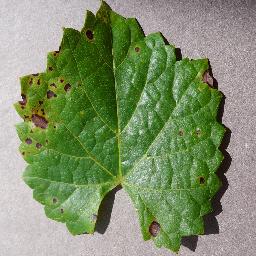

Supplement: Supplementary file 1 [file Data_Sheet_1.ZIP › training data/1014.jpg]

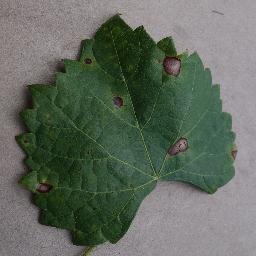

Supplement: Supplementary file 1 [file Data_Sheet_1.ZIP › training data/1015.jpg]

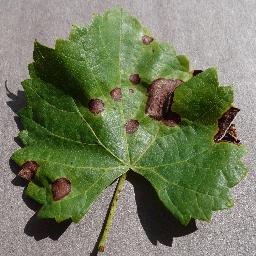

Supplement: Supplementary file 1 [file Data_Sheet_1.ZIP › training data/1016.jpg]

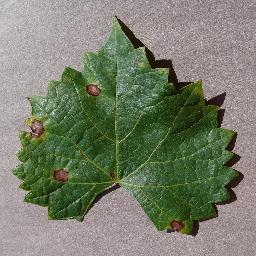

Supplement: Supplementary file 1 [file Data_Sheet_1.ZIP › training data/1017.jpg]

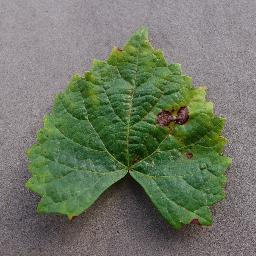

Supplement: Supplementary file 1 [file Data_Sheet_1.ZIP › training data/1018.jpg]

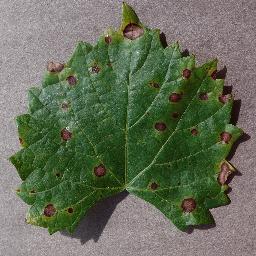

Supplement: Supplementary file 1 [file Data_Sheet_1.ZIP › training data/1019.jpg]

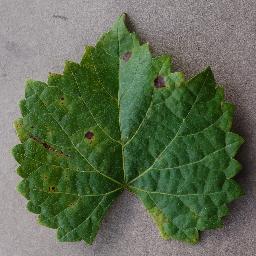

Supplement: Supplementary file 1 [file Data_Sheet_1.ZIP › training data/1020.jpg]

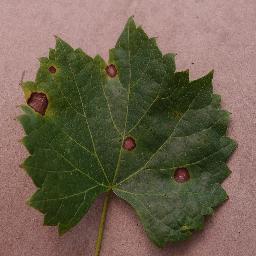

Supplement: Supplementary file 1 [file Data_Sheet_1.ZIP › training data/1021.jpg]

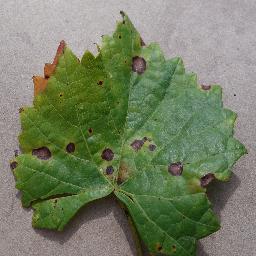

Supplement: Supplementary file 1 [file Data_Sheet_1.ZIP › training data/1022.jpg]

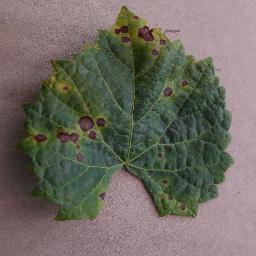

Supplement: Supplementary file 1 [file Data_Sheet_1.ZIP › training data/1023.jpg]

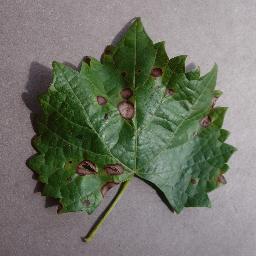

Supplement: Supplementary file 1 [file Data_Sheet_1.ZIP › training data/1024.jpg]

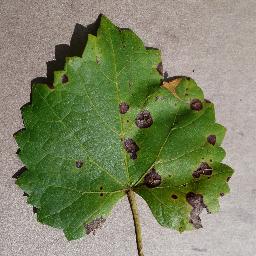

Supplement: Supplementary file 1 [file Data_Sheet_1.ZIP › training data/1025.jpg]

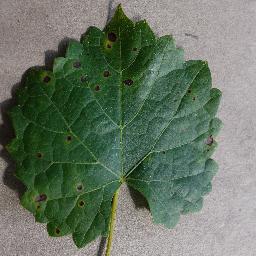

Supplement: Supplementary file 1 [file Data_Sheet_1.ZIP › training data/1026.jpg]

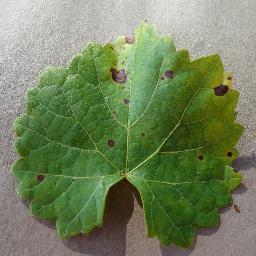

Supplement: Supplementary file 1 [file Data_Sheet_1.ZIP › training data/1027.jpg]

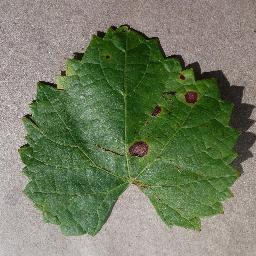

Supplement: Supplementary file 1 [file Data_Sheet_1.ZIP › training data/1028.jpg]

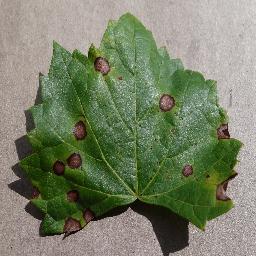

Supplement: Supplementary file 1 [file Data_Sheet_1.ZIP › training data/1029.jpg]

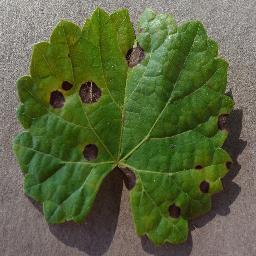

Supplement: Supplementary file 1 [file Data_Sheet_1.ZIP › training data/1030.jpg]

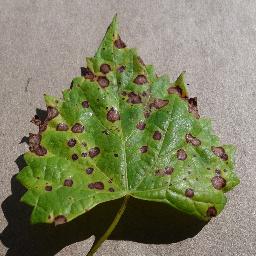

Supplement: Supplementary file 1 [file Data_Sheet_1.ZIP › training data/1031.jpg]

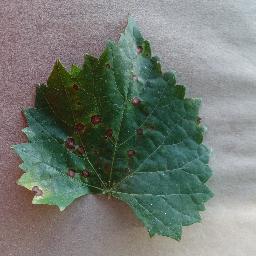

Supplement: Supplementary file 1 [file Data_Sheet_1.ZIP › training data/1032.jpg]

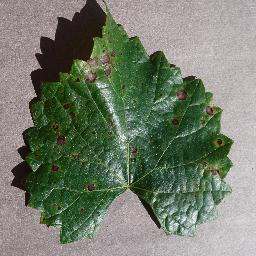

Supplement: Supplementary file 1 [file Data_Sheet_1.ZIP › training data/1033.jpg]

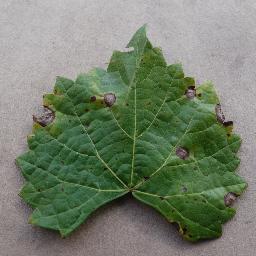

Supplement: Supplementary file 1 [file Data_Sheet_1.ZIP › training data/1034.jpg]

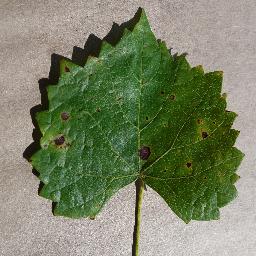

Supplement: Supplementary file 1 [file Data_Sheet_1.ZIP › training data/1035.jpg]

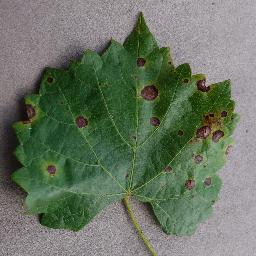

Supplement: Supplementary file 1 [file Data_Sheet_1.ZIP › training data/1036.jpg]

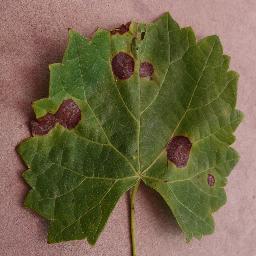

Supplement: Supplementary file 1 [file Data_Sheet_1.ZIP › training data/1037.jpg]

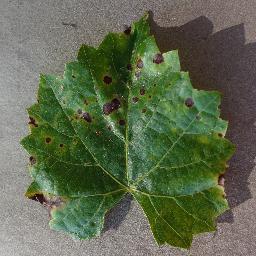

Supplement: Supplementary file 1 [file Data_Sheet_1.ZIP › training data/1038.jpg]

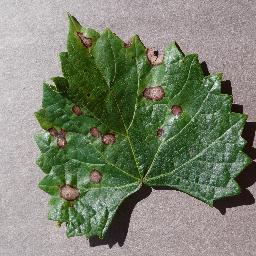

Supplement: Supplementary file 1 [file Data_Sheet_1.ZIP › training data/1039.jpg]

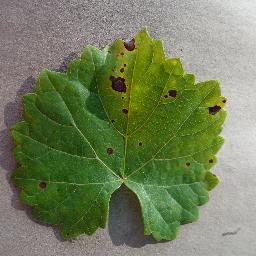

Supplement: Supplementary file 1 [file Data_Sheet_1.ZIP › training data/1040.jpg]

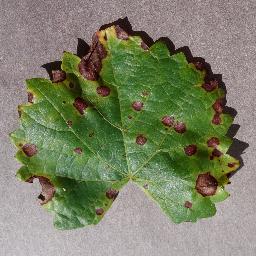

Supplement: Supplementary file 1 [file Data_Sheet_1.ZIP › training data/1041.jpg]

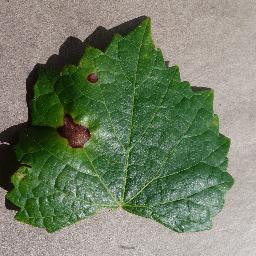

Supplement: Supplementary file 1 [file Data_Sheet_1.ZIP › training data/1042.jpg]

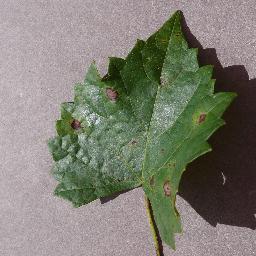

Supplement: Supplementary file 1 [file Data_Sheet_1.ZIP › training data/1043.jpg]

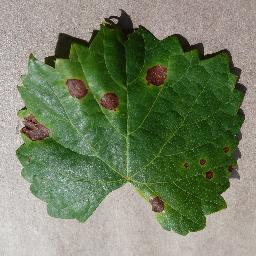

Supplement: Supplementary file 1 [file Data_Sheet_1.ZIP › training data/1044.jpg]

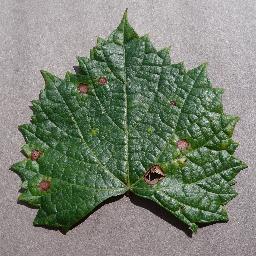

Supplement: Supplementary file 1 [file Data_Sheet_1.ZIP › training data/1045.jpg]

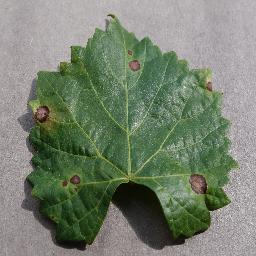

Supplement: Supplementary file 1 [file Data_Sheet_1.ZIP › training data/1046.jpg]

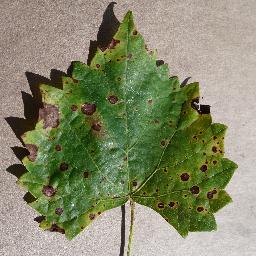

Supplement: Supplementary file 1 [file Data_Sheet_1.ZIP › training data/1047.jpg]

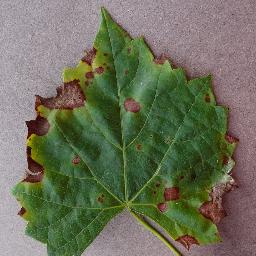

Supplement: Supplementary file 1 [file Data_Sheet_1.ZIP › training data/1048.jpg]

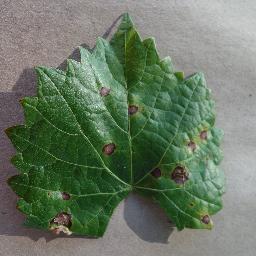

Supplement: Supplementary file 1 [file Data_Sheet_1.ZIP › training data/1049.jpg]

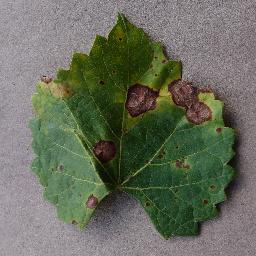

Supplement: Supplementary file 1 [file Data_Sheet_1.ZIP › training data/1050.jpg]

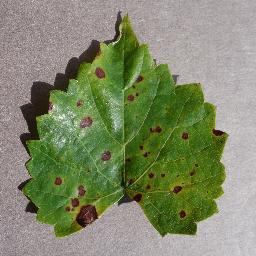

Supplement: Supplementary file 1 [file Data_Sheet_1.ZIP › training data/1051.jpg]

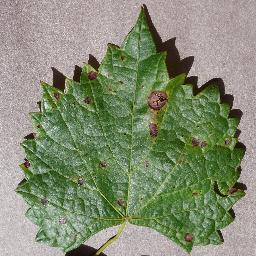

Supplement: Supplementary file 1 [file Data_Sheet_1.ZIP › training data/1052.jpg]

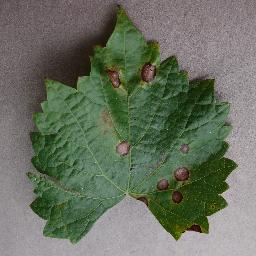

Supplement: Supplementary file 1 [file Data_Sheet_1.ZIP › training data/1053.jpg]

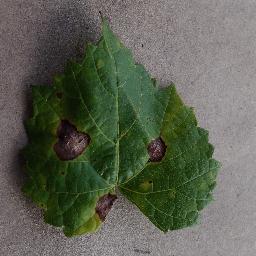

Supplement: Supplementary file 1 [file Data_Sheet_1.ZIP › training data/1054.jpg]

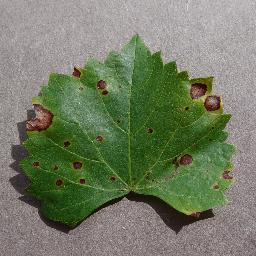

Supplement: Supplementary file 1 [file Data_Sheet_1.ZIP › training data/1055.jpg]

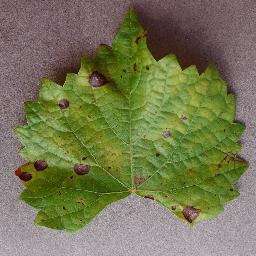

Supplement: Supplementary file 1 [file Data_Sheet_1.ZIP › training data/1056.jpg]

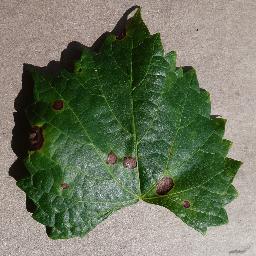

Supplement: Supplementary file 1 [file Data_Sheet_1.ZIP › training data/1057.jpg]

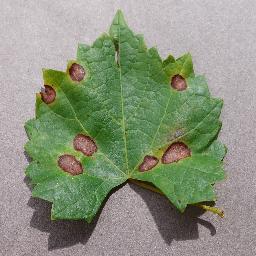

Supplement: Supplementary file 1 [file Data_Sheet_1.ZIP › training data/1058.jpg]

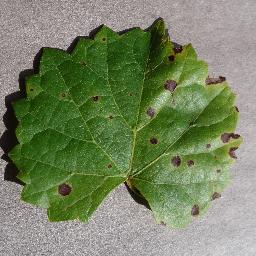

Supplement: Supplementary file 1 [file Data_Sheet_1.ZIP › training data/1059.jpg]

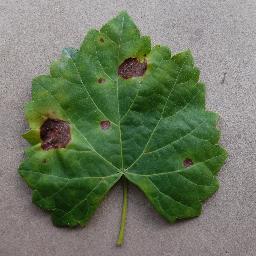

Supplement: Supplementary file 1 [file Data_Sheet_1.ZIP › training data/1060.jpg]

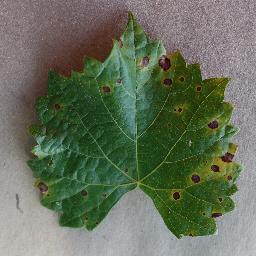

Supplement: Supplementary file 1 [file Data_Sheet_1.ZIP › training data/1061.jpg]

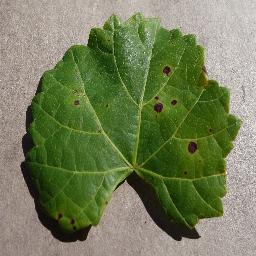

Supplement: Supplementary file 1 [file Data_Sheet_1.ZIP › training data/1062.jpg]

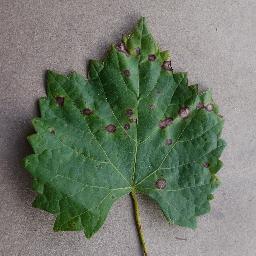

Supplement: Supplementary file 1 [file Data_Sheet_1.ZIP › training data/1063.jpg]

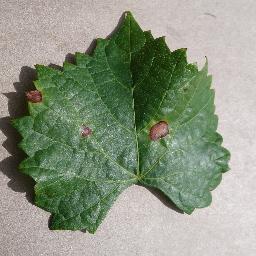

Supplement: Supplementary file 1 [file Data_Sheet_1.ZIP › training data/1064.jpg]

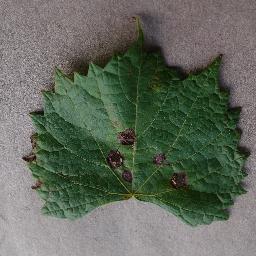

Supplement: Supplementary file 1 [file Data_Sheet_1.ZIP › training data/1065.jpg]

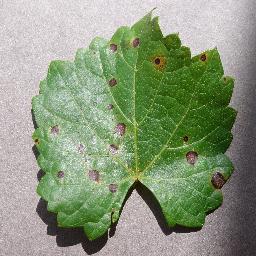

Supplement: Supplementary file 1 [file Data_Sheet_1.ZIP › training data/1066.jpg]

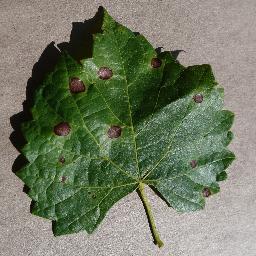

Supplement: Supplementary file 1 [file Data_Sheet_1.ZIP › training data/1067.jpg]

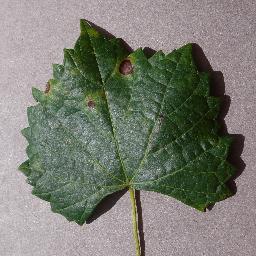

Supplement: Supplementary file 1 [file Data_Sheet_1.ZIP › training data/1068.jpg]

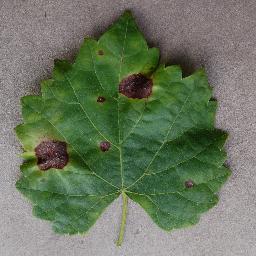

Supplement: Supplementary file 1 [file Data_Sheet_1.ZIP › training data/1069.jpg]

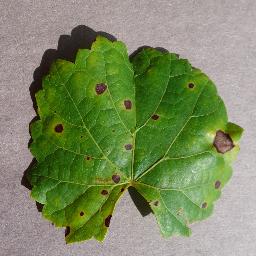

Supplement: Supplementary file 1 [file Data_Sheet_1.ZIP › training data/1070.jpg]

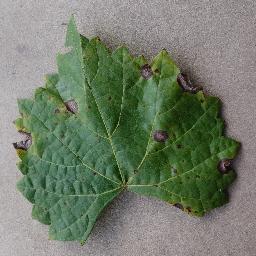

Supplement: Supplementary file 1 [file Data_Sheet_1.ZIP › training data/1071.jpg]

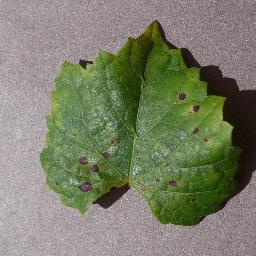

Supplement: Supplementary file 1 [file Data_Sheet_1.ZIP › training data/1072.jpg]

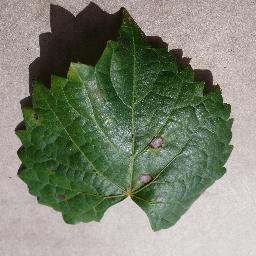

Supplement: Supplementary file 1 [file Data_Sheet_1.ZIP › training data/1073.jpg]

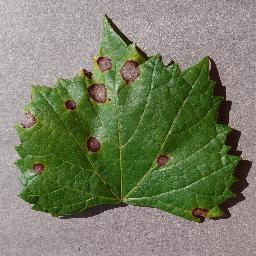

Supplement: Supplementary file 1 [file Data_Sheet_1.ZIP › training data/1074.jpg]

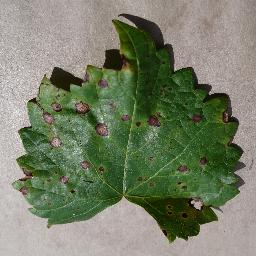

Supplement: Supplementary file 1 [file Data_Sheet_1.ZIP › training data/1075.jpg]

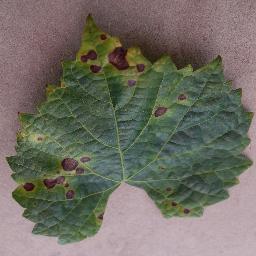

Supplement: Supplementary file 1 [file Data_Sheet_1.ZIP › training data/1076.jpg]

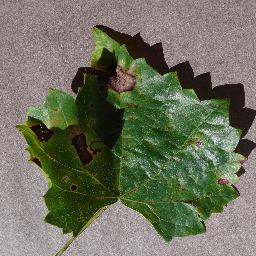

Supplement: Supplementary file 1 [file Data_Sheet_1.ZIP › training data/1077.jpg]

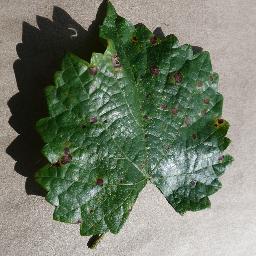

Supplement: Supplementary file 1 [file Data_Sheet_1.ZIP › training data/1078.jpg]

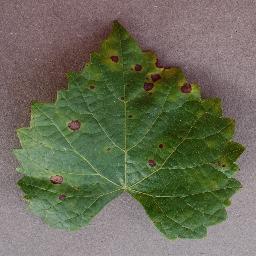

Supplement: Supplementary file 1 [file Data_Sheet_1.ZIP › training data/1079.jpg]

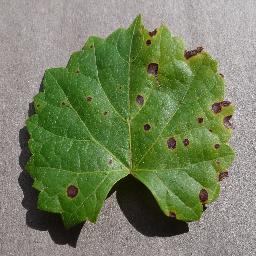

Supplement: Supplementary file 1 [file Data_Sheet_1.ZIP › training data/108.JPG]

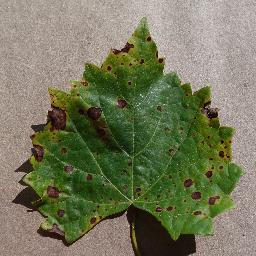

Supplement: Supplementary file 1 [file Data_Sheet_1.ZIP › training data/1080.jpg]

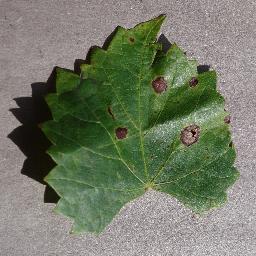

Supplement: Supplementary file 1 [file Data_Sheet_1.ZIP › training data/1081.jpg]

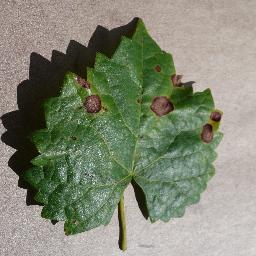

Supplement: Supplementary file 1 [file Data_Sheet_1.ZIP › training data/1082.jpg]

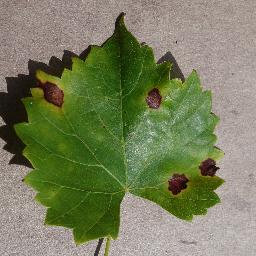

Supplement: Supplementary file 1 [file Data_Sheet_1.ZIP › training data/1083.jpg]

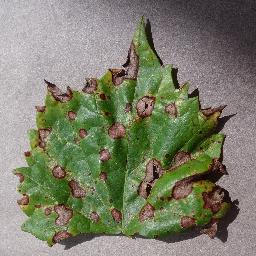

Supplement: Supplementary file 1 [file Data_Sheet_1.ZIP › training data/1084.jpg]

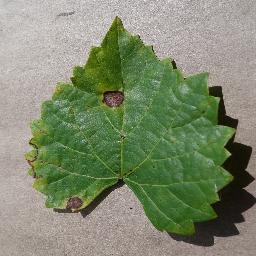

Supplement: Supplementary file 1 [file Data_Sheet_1.ZIP › training data/1085.jpg]

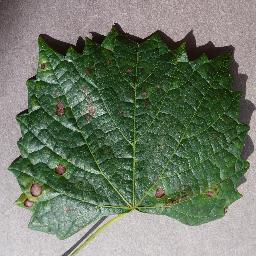

Supplement: Supplementary file 1 [file Data_Sheet_1.ZIP › training data/1086.jpg]

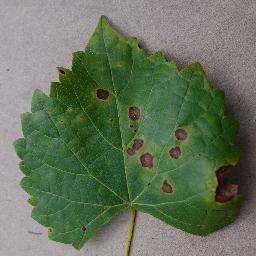

Supplement: Supplementary file 1 [file Data_Sheet_1.ZIP › training data/1087.jpg]

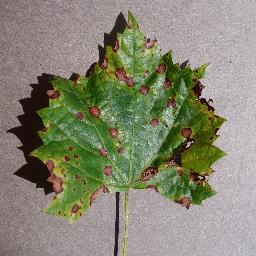

Supplement: Supplementary file 1 [file Data_Sheet_1.ZIP › training data/1088.jpg]

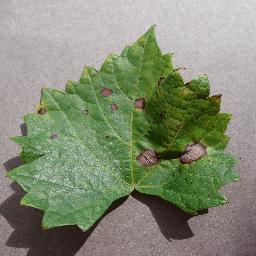

Supplement: Supplementary file 1 [file Data_Sheet_1.ZIP › training data/1089.jpg]

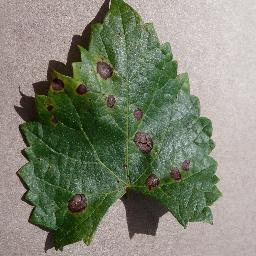

Supplement: Supplementary file 1 [file Data_Sheet_1.ZIP › training data/109.JPG]

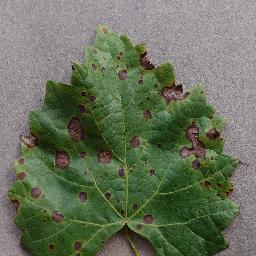

Supplement: Supplementary file 1 [file Data_Sheet_1.ZIP › training data/1090.jpg]

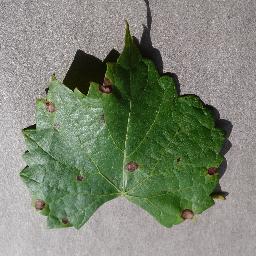

Supplement: Supplementary file 1 [file Data_Sheet_1.ZIP › training data/1091.jpg]

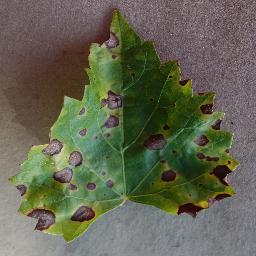

Supplement: Supplementary file 1 [file Data_Sheet_1.ZIP › training data/1092.jpg]

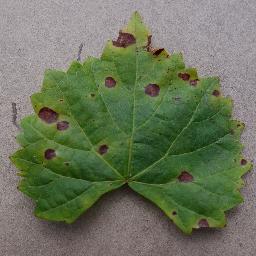

Supplement: Supplementary file 1 [file Data_Sheet_1.ZIP › training data/1093.jpg]

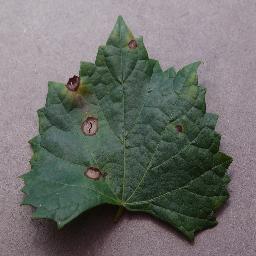

Supplement: Supplementary file 1 [file Data_Sheet_1.ZIP › training data/1094.jpg]

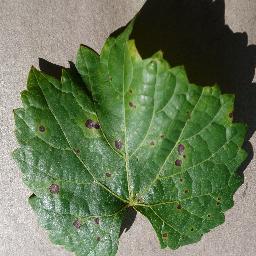

Supplement: Supplementary file 1 [file Data_Sheet_1.ZIP › training data/1095.jpg]

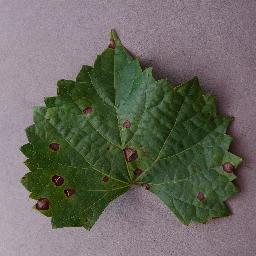

Supplement: Supplementary file 1 [file Data_Sheet_1.ZIP › training data/1096.jpg]

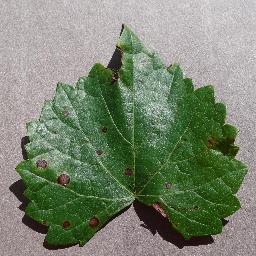

Supplement: Supplementary file 1 [file Data_Sheet_1.ZIP › training data/1097.jpg]
