# Supplementary figures and images for: Grape Leaf Black Rot Detection Based on Super-Resolution Image Enhancement and Deep Learning (part 3 of 6)
Source: Front Plant Sci. 2021 Jun 29;12:695749. doi: 10.3389/fpls.2021.695749 (PMC8277438; doi:10.3389/fpls.2021.695749)

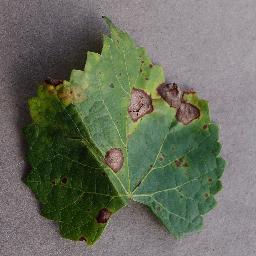

Supplement: Supplementary file 2 [file Data_Sheet_2.ZIP › test_pv/106.JPG]

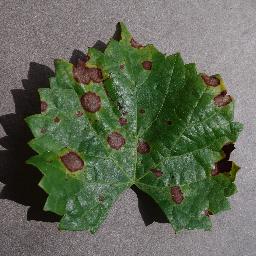

Supplement: Supplementary file 2 [file Data_Sheet_2.ZIP › test_pv/107.JPG]

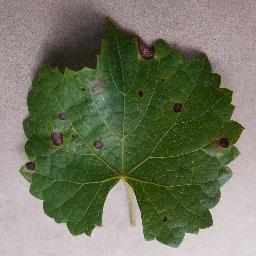

Supplement: Supplementary file 2 [file Data_Sheet_2.ZIP › test_pv/11.JPG]

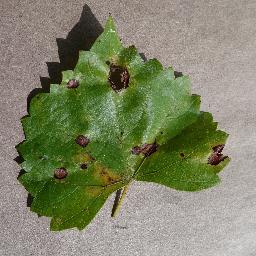

Supplement: Supplementary file 2 [file Data_Sheet_2.ZIP › test_pv/12.JPG]

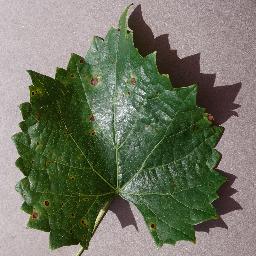

Supplement: Supplementary file 2 [file Data_Sheet_2.ZIP › test_pv/13.JPG]

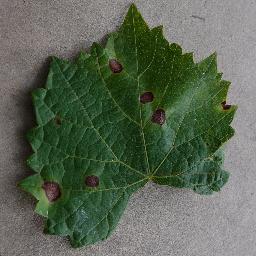

Supplement: Supplementary file 2 [file Data_Sheet_2.ZIP › test_pv/14.JPG]

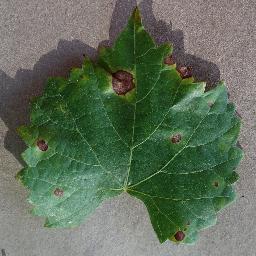

Supplement: Supplementary file 2 [file Data_Sheet_2.ZIP › test_pv/15.JPG]

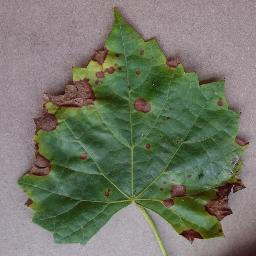

Supplement: Supplementary file 2 [file Data_Sheet_2.ZIP › test_pv/16.JPG]

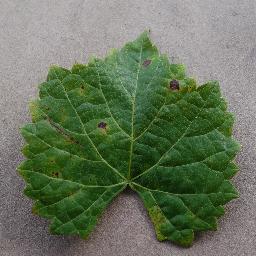

Supplement: Supplementary file 2 [file Data_Sheet_2.ZIP › test_pv/17.JPG]

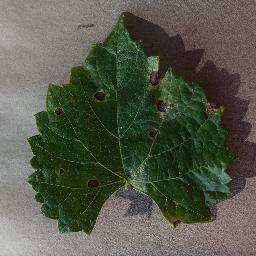

Supplement: Supplementary file 2 [file Data_Sheet_2.ZIP › test_pv/18.JPG]

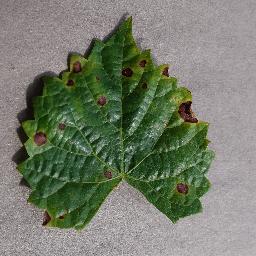

Supplement: Supplementary file 2 [file Data_Sheet_2.ZIP › test_pv/19.JPG]

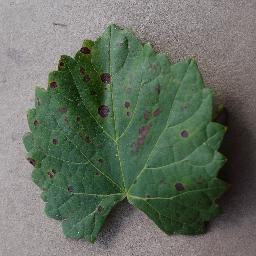

Supplement: Supplementary file 2 [file Data_Sheet_2.ZIP › test_pv/2.JPG]

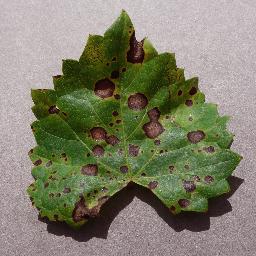

Supplement: Supplementary file 2 [file Data_Sheet_2.ZIP › test_pv/20.JPG]

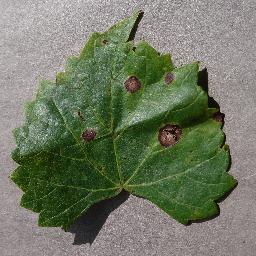

Supplement: Supplementary file 2 [file Data_Sheet_2.ZIP › test_pv/21.JPG]

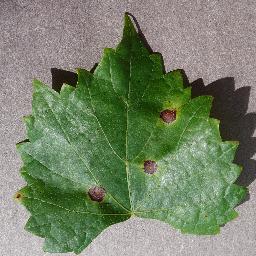

Supplement: Supplementary file 2 [file Data_Sheet_2.ZIP › test_pv/22.JPG]

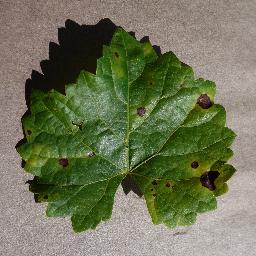

Supplement: Supplementary file 2 [file Data_Sheet_2.ZIP › test_pv/23.JPG]

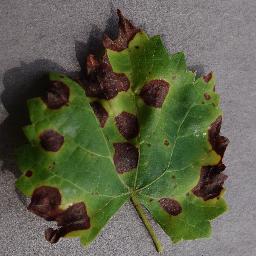

Supplement: Supplementary file 2 [file Data_Sheet_2.ZIP › test_pv/24.JPG]

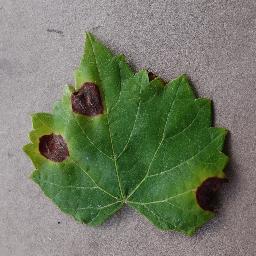

Supplement: Supplementary file 2 [file Data_Sheet_2.ZIP › test_pv/25.JPG]

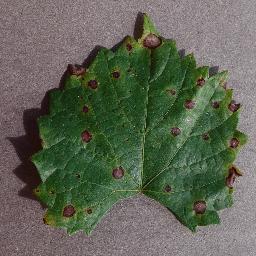

Supplement: Supplementary file 2 [file Data_Sheet_2.ZIP › test_pv/26.JPG]

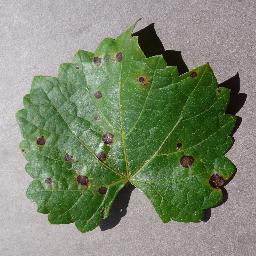

Supplement: Supplementary file 2 [file Data_Sheet_2.ZIP › test_pv/27.JPG]

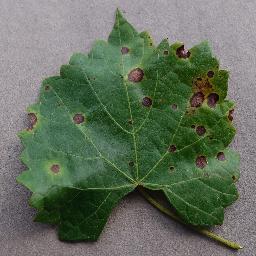

Supplement: Supplementary file 2 [file Data_Sheet_2.ZIP › test_pv/28.JPG]

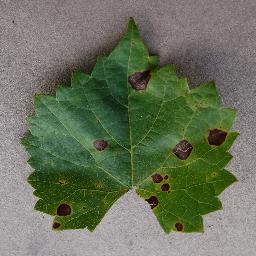

Supplement: Supplementary file 2 [file Data_Sheet_2.ZIP › test_pv/29.JPG]

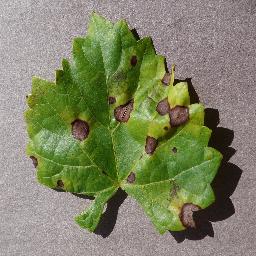

Supplement: Supplementary file 2 [file Data_Sheet_2.ZIP › test_pv/3.JPG]

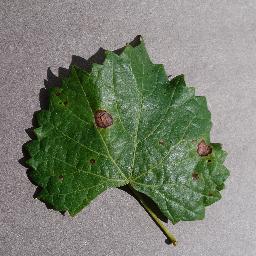

Supplement: Supplementary file 2 [file Data_Sheet_2.ZIP › test_pv/30.JPG]

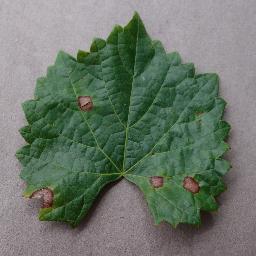

Supplement: Supplementary file 2 [file Data_Sheet_2.ZIP › test_pv/31.JPG]

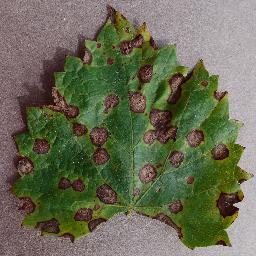

Supplement: Supplementary file 2 [file Data_Sheet_2.ZIP › test_pv/32.JPG]

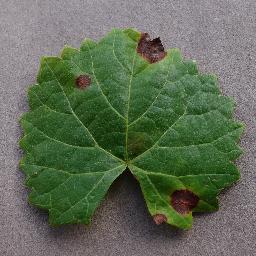

Supplement: Supplementary file 2 [file Data_Sheet_2.ZIP › test_pv/33.JPG]

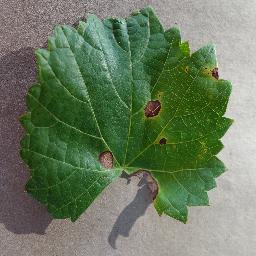

Supplement: Supplementary file 2 [file Data_Sheet_2.ZIP › test_pv/34.JPG]

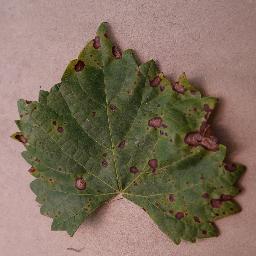

Supplement: Supplementary file 2 [file Data_Sheet_2.ZIP › test_pv/35.JPG]

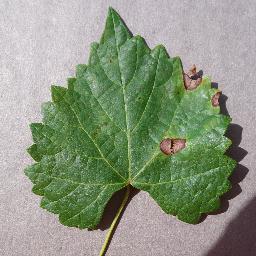

Supplement: Supplementary file 2 [file Data_Sheet_2.ZIP › test_pv/36.JPG]

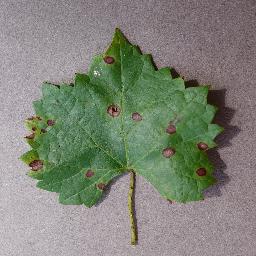

Supplement: Supplementary file 2 [file Data_Sheet_2.ZIP › test_pv/37.JPG]

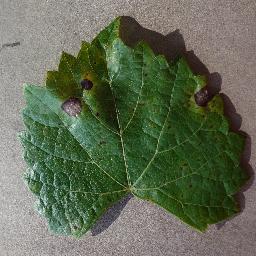

Supplement: Supplementary file 2 [file Data_Sheet_2.ZIP › test_pv/38.JPG]

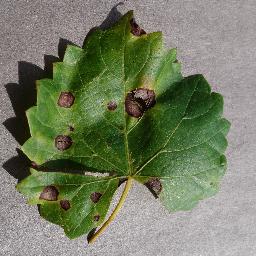

Supplement: Supplementary file 2 [file Data_Sheet_2.ZIP › test_pv/39.JPG]

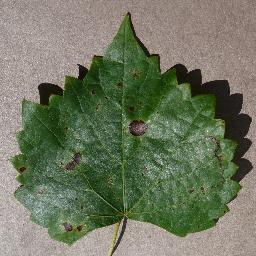

Supplement: Supplementary file 2 [file Data_Sheet_2.ZIP › test_pv/4.JPG]

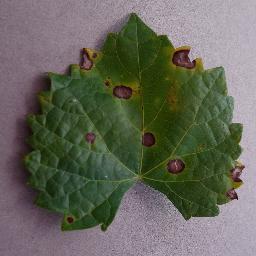

Supplement: Supplementary file 2 [file Data_Sheet_2.ZIP › test_pv/40.JPG]

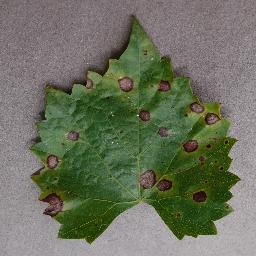

Supplement: Supplementary file 2 [file Data_Sheet_2.ZIP › test_pv/41.JPG]

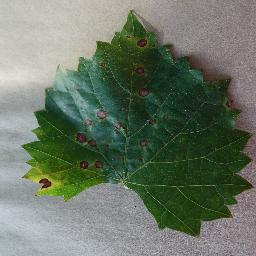

Supplement: Supplementary file 2 [file Data_Sheet_2.ZIP › test_pv/42.JPG]

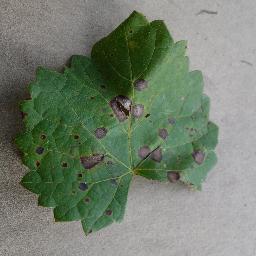

Supplement: Supplementary file 2 [file Data_Sheet_2.ZIP › test_pv/43.JPG]

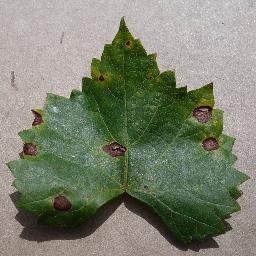

Supplement: Supplementary file 2 [file Data_Sheet_2.ZIP › test_pv/44.JPG]

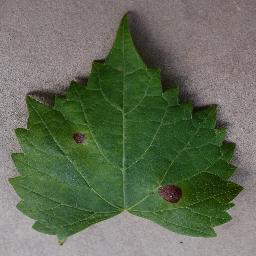

Supplement: Supplementary file 2 [file Data_Sheet_2.ZIP › test_pv/45.JPG]

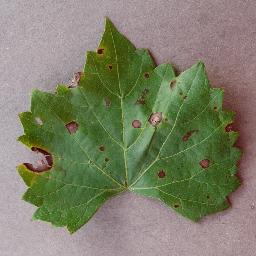

Supplement: Supplementary file 2 [file Data_Sheet_2.ZIP › test_pv/46.JPG]

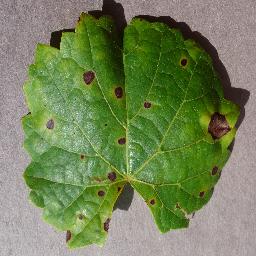

Supplement: Supplementary file 2 [file Data_Sheet_2.ZIP › test_pv/47.JPG]

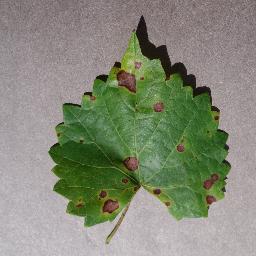

Supplement: Supplementary file 2 [file Data_Sheet_2.ZIP › test_pv/48.JPG]

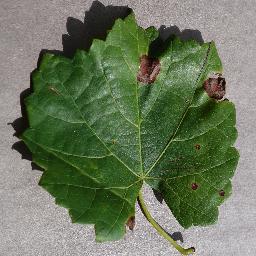

Supplement: Supplementary file 2 [file Data_Sheet_2.ZIP › test_pv/49.JPG]

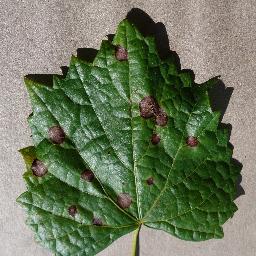

Supplement: Supplementary file 2 [file Data_Sheet_2.ZIP › test_pv/5.JPG]

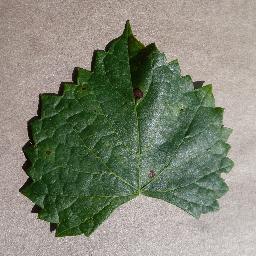

Supplement: Supplementary file 2 [file Data_Sheet_2.ZIP › test_pv/50.JPG]

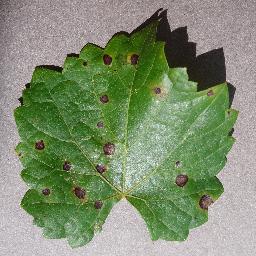

Supplement: Supplementary file 2 [file Data_Sheet_2.ZIP › test_pv/51.JPG]

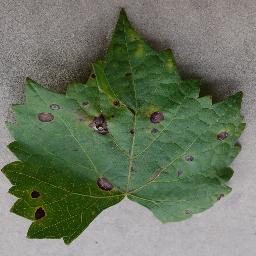

Supplement: Supplementary file 2 [file Data_Sheet_2.ZIP › test_pv/52.JPG]

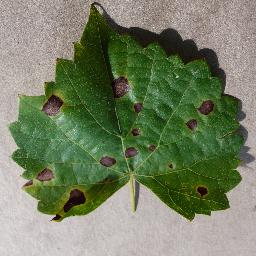

Supplement: Supplementary file 2 [file Data_Sheet_2.ZIP › test_pv/53.JPG]

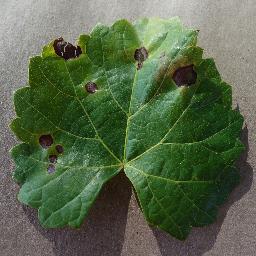

Supplement: Supplementary file 2 [file Data_Sheet_2.ZIP › test_pv/54.JPG]

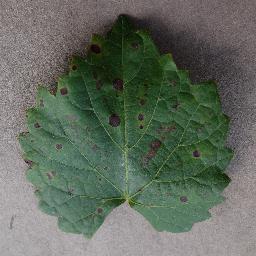

Supplement: Supplementary file 2 [file Data_Sheet_2.ZIP › test_pv/55.JPG]

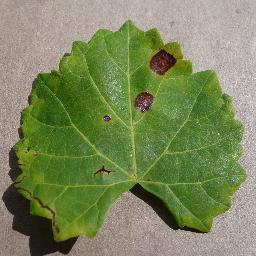

Supplement: Supplementary file 2 [file Data_Sheet_2.ZIP › test_pv/56.JPG]

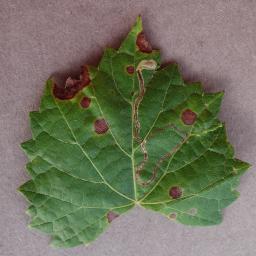

Supplement: Supplementary file 2 [file Data_Sheet_2.ZIP › test_pv/57.JPG]

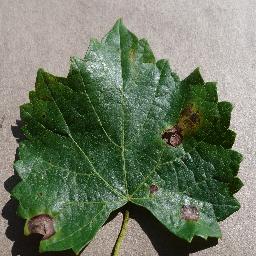

Supplement: Supplementary file 2 [file Data_Sheet_2.ZIP › test_pv/58.JPG]

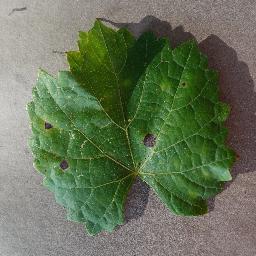

Supplement: Supplementary file 2 [file Data_Sheet_2.ZIP › test_pv/59.JPG]

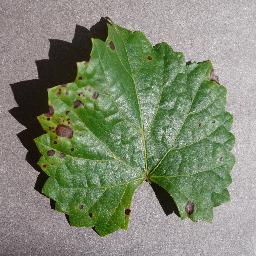

Supplement: Supplementary file 2 [file Data_Sheet_2.ZIP › test_pv/6.JPG]

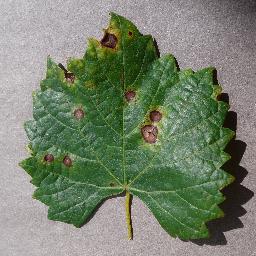

Supplement: Supplementary file 2 [file Data_Sheet_2.ZIP › test_pv/60.JPG]

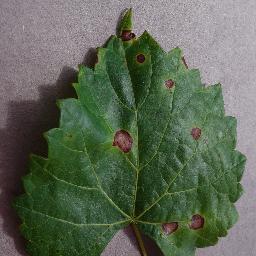

Supplement: Supplementary file 2 [file Data_Sheet_2.ZIP › test_pv/61.JPG]

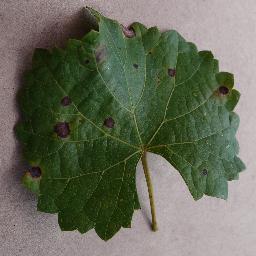

Supplement: Supplementary file 2 [file Data_Sheet_2.ZIP › test_pv/62.JPG]

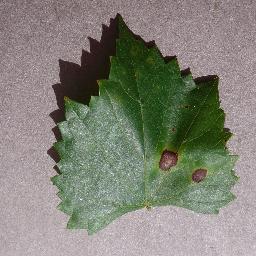

Supplement: Supplementary file 2 [file Data_Sheet_2.ZIP › test_pv/63.JPG]

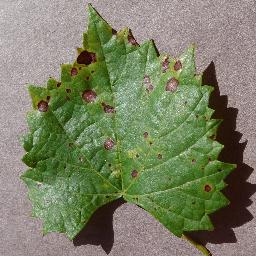

Supplement: Supplementary file 2 [file Data_Sheet_2.ZIP › test_pv/64.JPG]

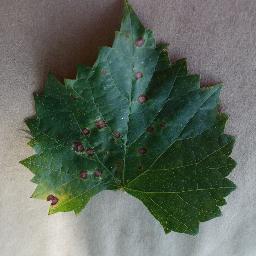

Supplement: Supplementary file 2 [file Data_Sheet_2.ZIP › test_pv/65.JPG]

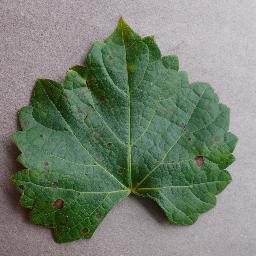

Supplement: Supplementary file 2 [file Data_Sheet_2.ZIP › test_pv/66.JPG]

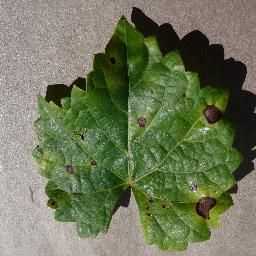

Supplement: Supplementary file 2 [file Data_Sheet_2.ZIP › test_pv/67.JPG]

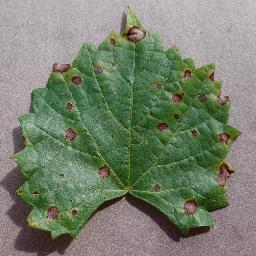

Supplement: Supplementary file 2 [file Data_Sheet_2.ZIP › test_pv/68.JPG]

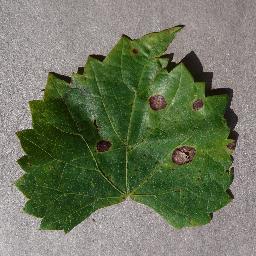

Supplement: Supplementary file 2 [file Data_Sheet_2.ZIP › test_pv/69.JPG]

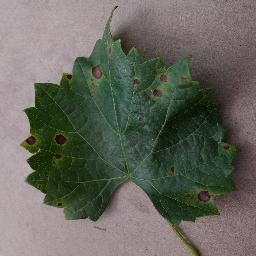

Supplement: Supplementary file 2 [file Data_Sheet_2.ZIP › test_pv/7.JPG]

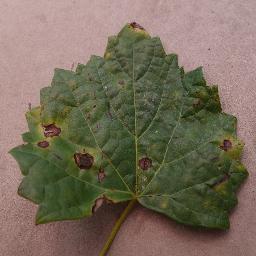

Supplement: Supplementary file 2 [file Data_Sheet_2.ZIP › test_pv/70.JPG]

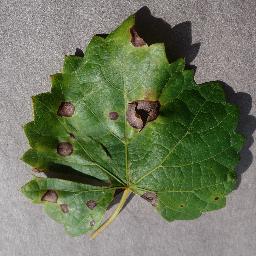

Supplement: Supplementary file 2 [file Data_Sheet_2.ZIP › test_pv/71.JPG]

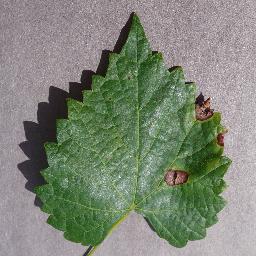

Supplement: Supplementary file 2 [file Data_Sheet_2.ZIP › test_pv/72.JPG]

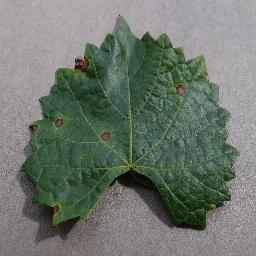

Supplement: Supplementary file 2 [file Data_Sheet_2.ZIP › test_pv/73.JPG]

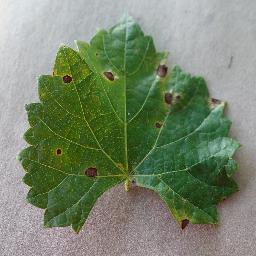

Supplement: Supplementary file 2 [file Data_Sheet_2.ZIP › test_pv/74.JPG]

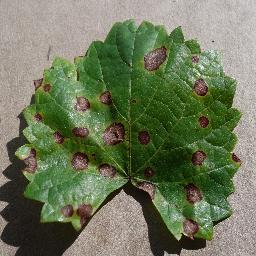

Supplement: Supplementary file 2 [file Data_Sheet_2.ZIP › test_pv/75.JPG]

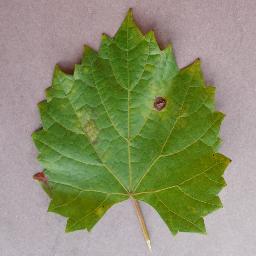

Supplement: Supplementary file 2 [file Data_Sheet_2.ZIP › test_pv/76.JPG]

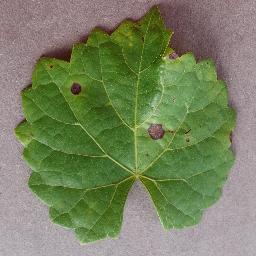

Supplement: Supplementary file 2 [file Data_Sheet_2.ZIP › test_pv/77.JPG]

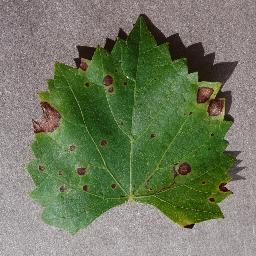

Supplement: Supplementary file 2 [file Data_Sheet_2.ZIP › test_pv/78.JPG]

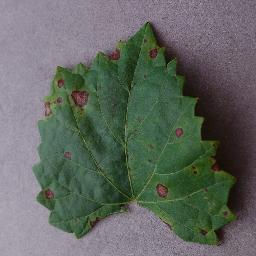

Supplement: Supplementary file 2 [file Data_Sheet_2.ZIP › test_pv/79.JPG]

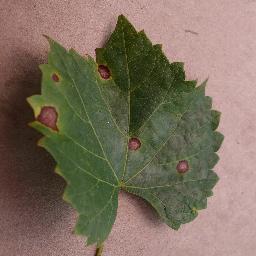

Supplement: Supplementary file 2 [file Data_Sheet_2.ZIP › test_pv/8.JPG]

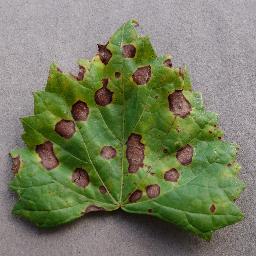

Supplement: Supplementary file 2 [file Data_Sheet_2.ZIP › test_pv/80.JPG]

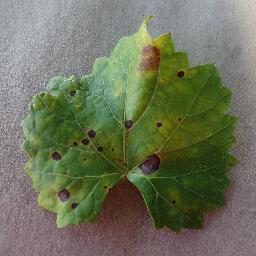

Supplement: Supplementary file 2 [file Data_Sheet_2.ZIP › test_pv/81.JPG]

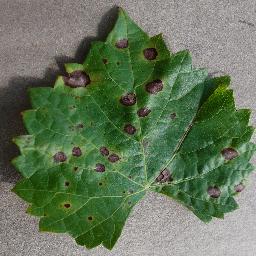

Supplement: Supplementary file 2 [file Data_Sheet_2.ZIP › test_pv/82.JPG]

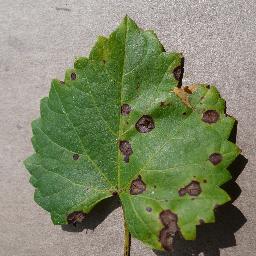

Supplement: Supplementary file 2 [file Data_Sheet_2.ZIP › test_pv/83.JPG]

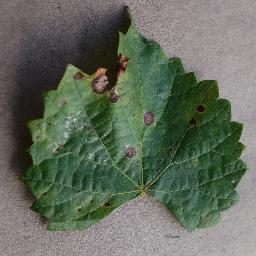

Supplement: Supplementary file 2 [file Data_Sheet_2.ZIP › test_pv/84.JPG]

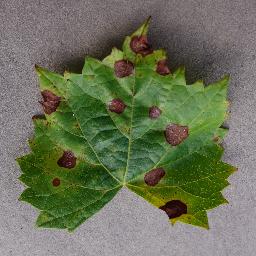

Supplement: Supplementary file 2 [file Data_Sheet_2.ZIP › test_pv/85.JPG]

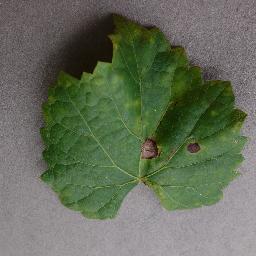

Supplement: Supplementary file 2 [file Data_Sheet_2.ZIP › test_pv/86.JPG]

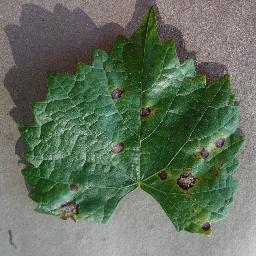

Supplement: Supplementary file 2 [file Data_Sheet_2.ZIP › test_pv/87.JPG]

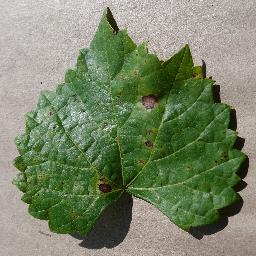

Supplement: Supplementary file 2 [file Data_Sheet_2.ZIP › test_pv/88.JPG]

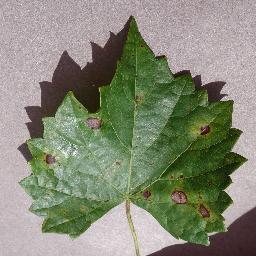

Supplement: Supplementary file 2 [file Data_Sheet_2.ZIP › test_pv/89.JPG]

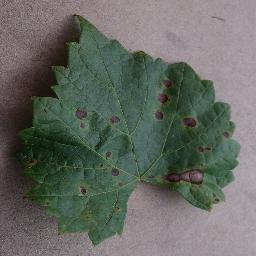

Supplement: Supplementary file 2 [file Data_Sheet_2.ZIP › test_pv/9.JPG]

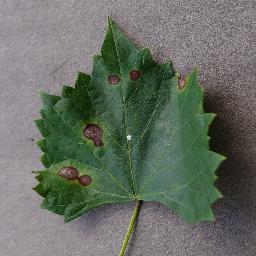

Supplement: Supplementary file 2 [file Data_Sheet_2.ZIP › test_pv/90.JPG]

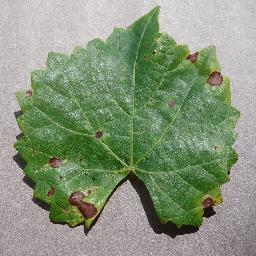

Supplement: Supplementary file 2 [file Data_Sheet_2.ZIP › test_pv/91.JPG]

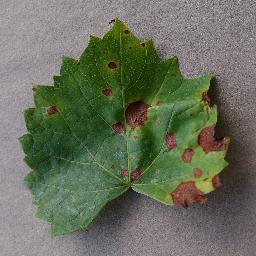

Supplement: Supplementary file 2 [file Data_Sheet_2.ZIP › test_pv/92.JPG]

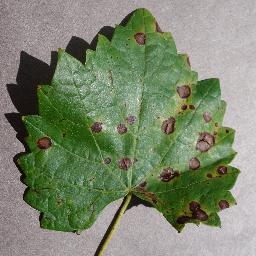

Supplement: Supplementary file 2 [file Data_Sheet_2.ZIP › test_pv/93.JPG]

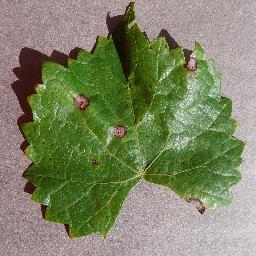

Supplement: Supplementary file 2 [file Data_Sheet_2.ZIP › test_pv/94.JPG]

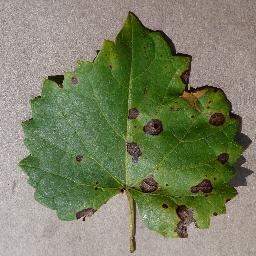

Supplement: Supplementary file 2 [file Data_Sheet_2.ZIP › test_pv/95.JPG]

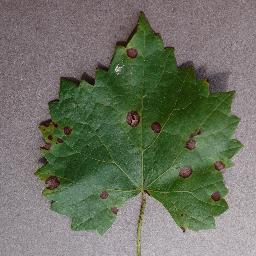

Supplement: Supplementary file 2 [file Data_Sheet_2.ZIP › test_pv/96.JPG]

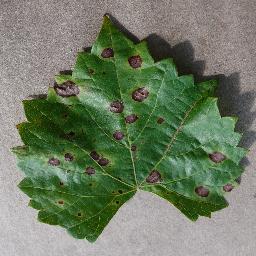

Supplement: Supplementary file 2 [file Data_Sheet_2.ZIP › test_pv/97.JPG]

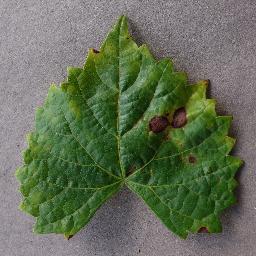

Supplement: Supplementary file 2 [file Data_Sheet_2.ZIP › test_pv/98.JPG]

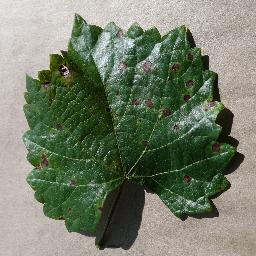

Supplement: Supplementary file 2 [file Data_Sheet_2.ZIP › test_pv/99.JPG]

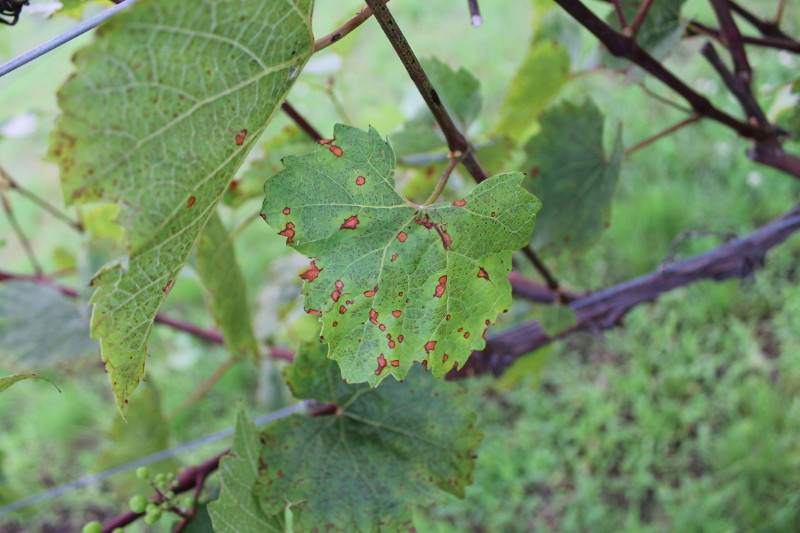

Supplement: Supplementary file 3 [file Data_Sheet_3.ZIP › test_orchard_1/0.jpg]
