# Supplementary figures and images for: Grape Leaf Black Rot Detection Based on Super-Resolution Image Enhancement and Deep Learning (part 4 of 6)
Source: Front Plant Sci. 2021 Jun 29;12:695749. doi: 10.3389/fpls.2021.695749 (PMC8277438; doi:10.3389/fpls.2021.695749)

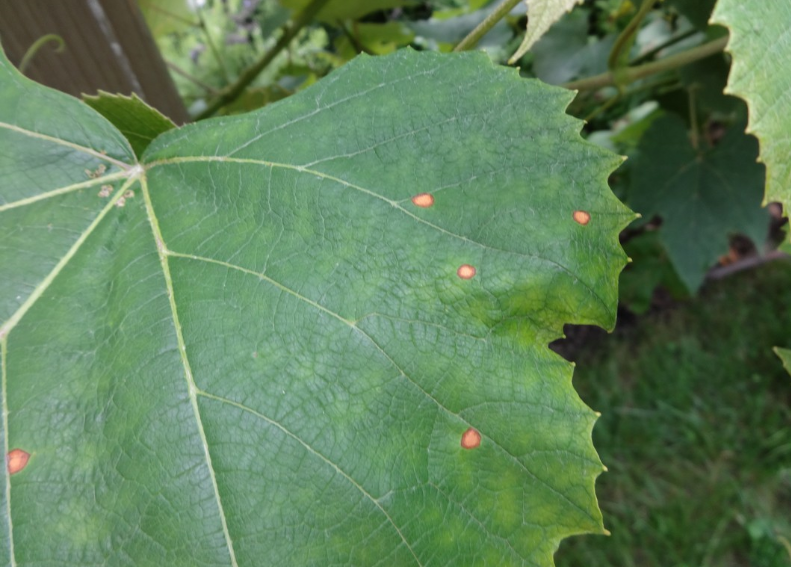

Supplement: Supplementary file 3 [file Data_Sheet_3.ZIP › test_orchard_1/1.png]

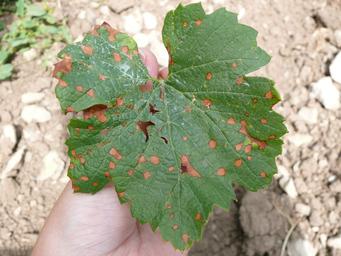

Supplement: Supplementary file 3 [file Data_Sheet_3.ZIP › test_orchard_1/2.jpg]

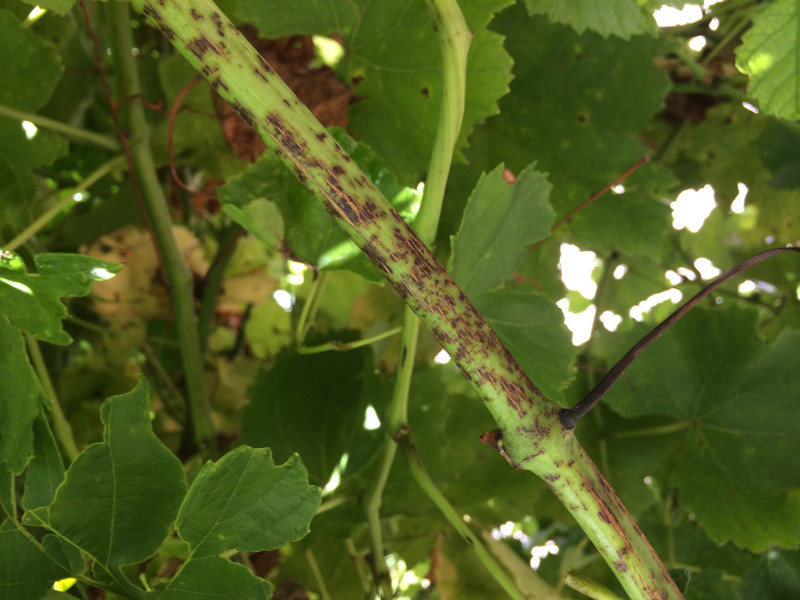

Supplement: Supplementary file 3 [file Data_Sheet_3.ZIP › test_orchard_1/3.jpg]

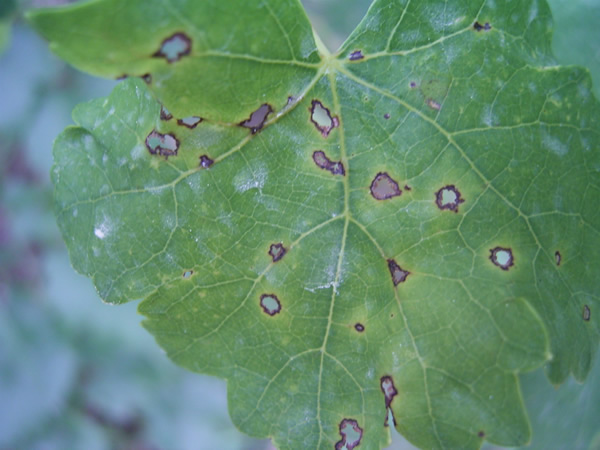

Supplement: Supplementary file 3 [file Data_Sheet_3.ZIP › test_orchard_1/4.jpg]

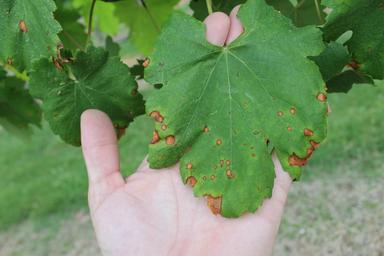

Supplement: Supplementary file 3 [file Data_Sheet_3.ZIP › test_orchard_1/5.jpg]

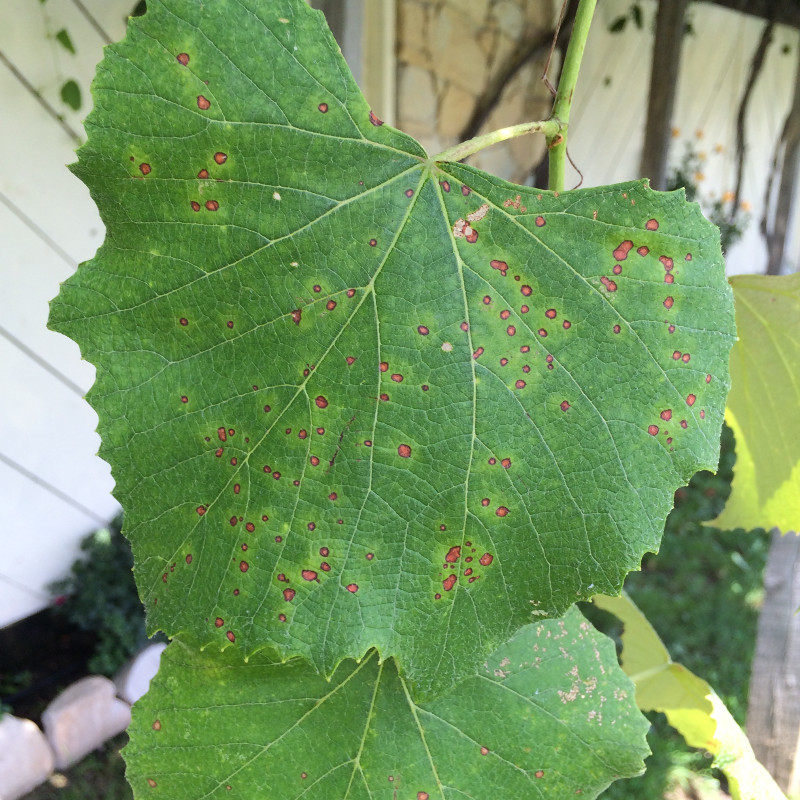

Supplement: Supplementary file 3 [file Data_Sheet_3.ZIP › test_orchard_1/6.jpg]

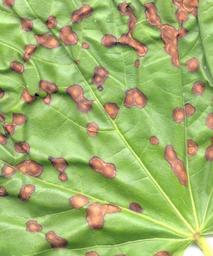

Supplement: Supplementary file 3 [file Data_Sheet_3.ZIP › test_orchard_1/7.jpg]

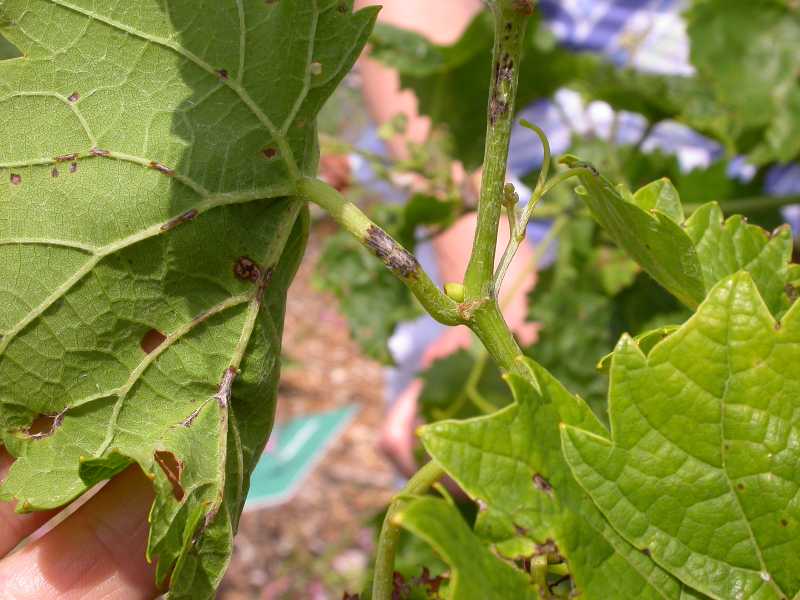

Supplement: Supplementary file 3 [file Data_Sheet_3.ZIP › test_orchard_1/8.jpg]

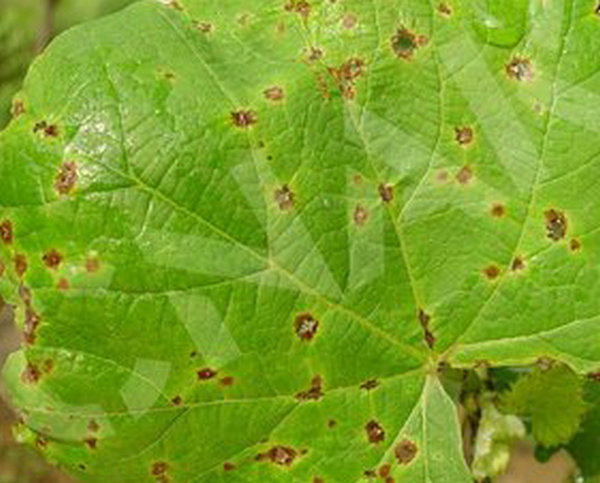

Supplement: Supplementary file 3 [file Data_Sheet_3.ZIP › test_orchard_1/9.jpg]

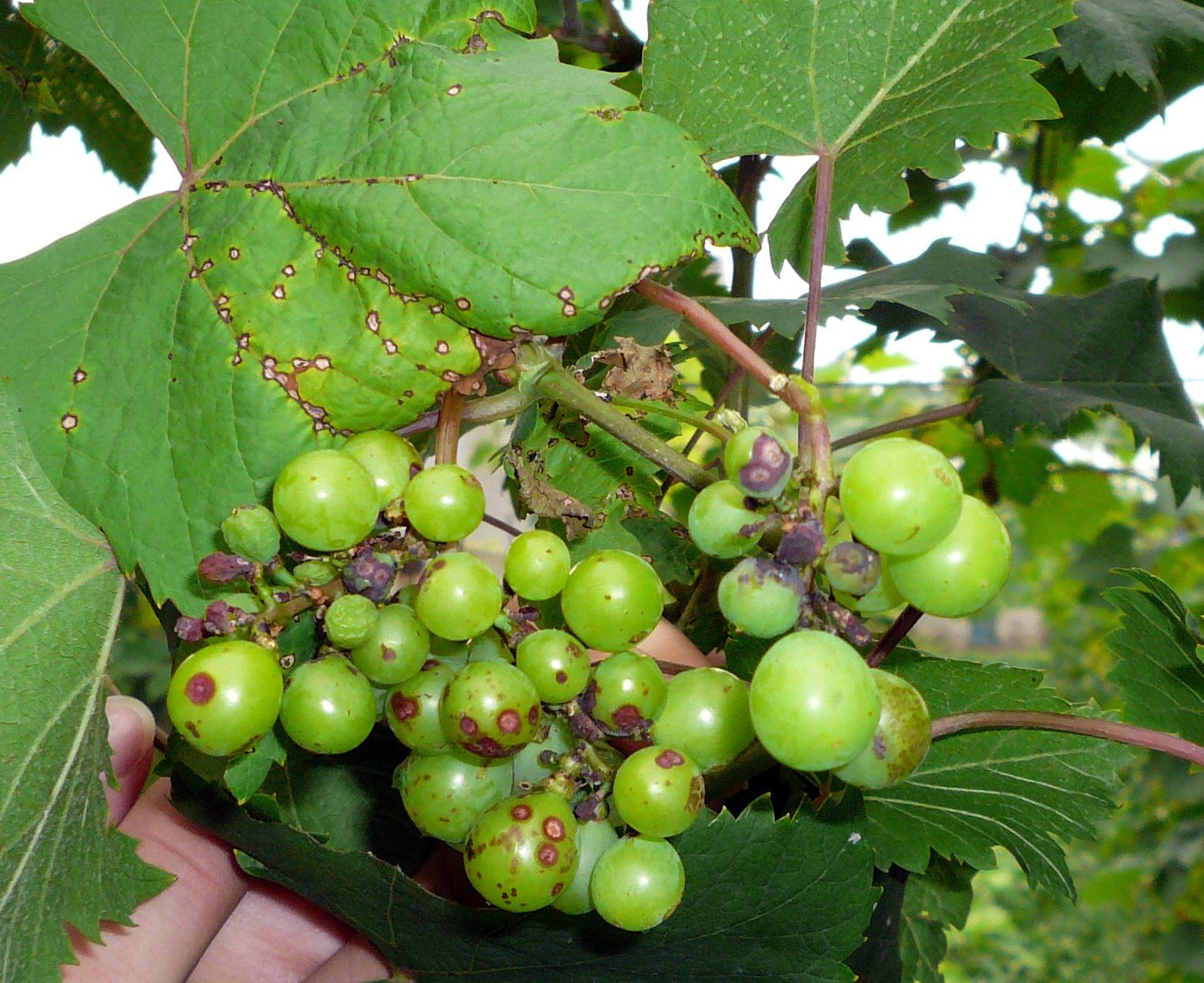

Supplement: Supplementary file 3 [file Data_Sheet_3.ZIP › test_orchard_1/10.jpg]

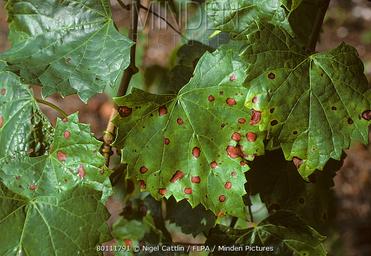

Supplement: Supplementary file 3 [file Data_Sheet_3.ZIP › test_orchard_1/11.jpg]

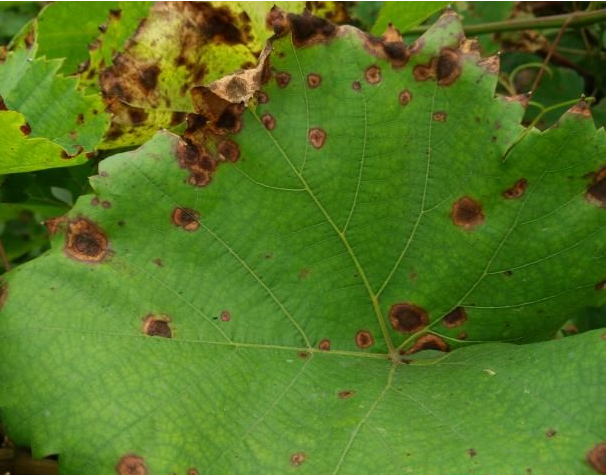

Supplement: Supplementary file 3 [file Data_Sheet_3.ZIP › test_orchard_1/12.png]

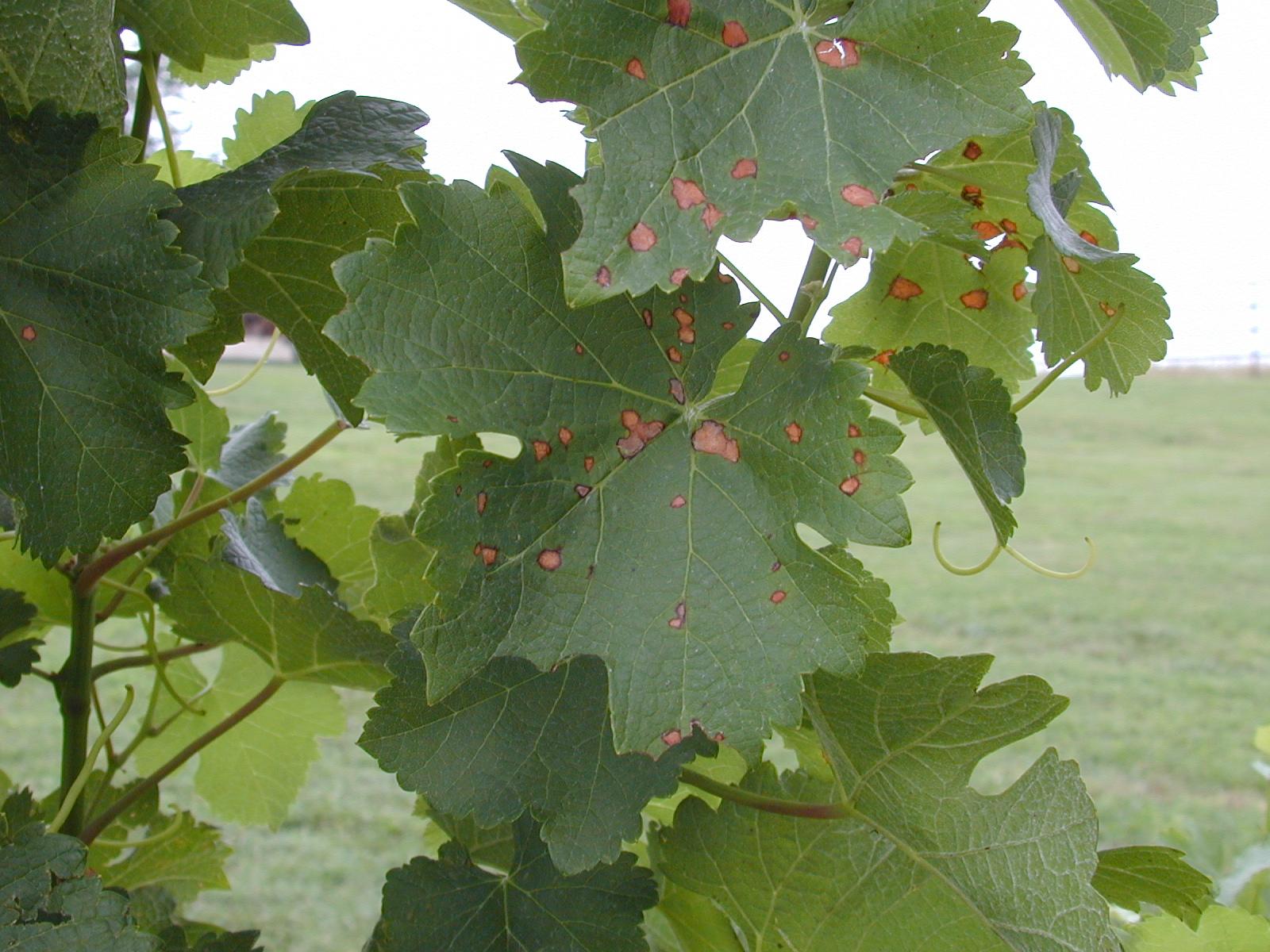

Supplement: Supplementary file 3 [file Data_Sheet_3.ZIP › test_orchard_1/13.jpg]

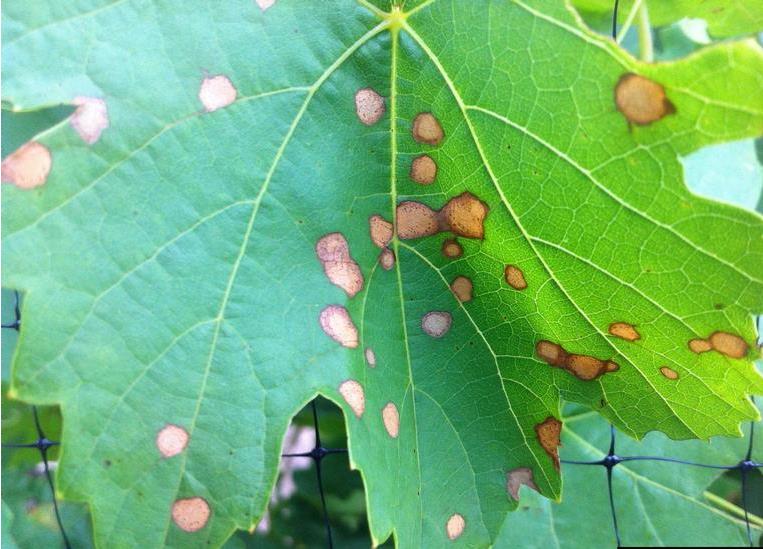

Supplement: Supplementary file 3 [file Data_Sheet_3.ZIP › test_orchard_1/14.jpg]

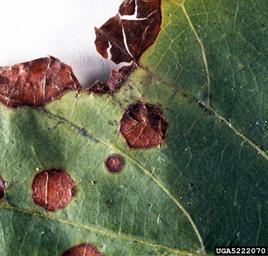

Supplement: Supplementary file 3 [file Data_Sheet_3.ZIP › test_orchard_1/15.jpg]

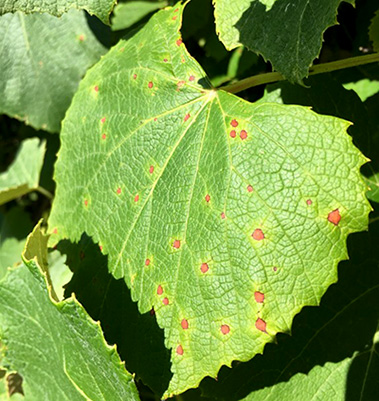

Supplement: Supplementary file 3 [file Data_Sheet_3.ZIP › test_orchard_1/16.jpg]

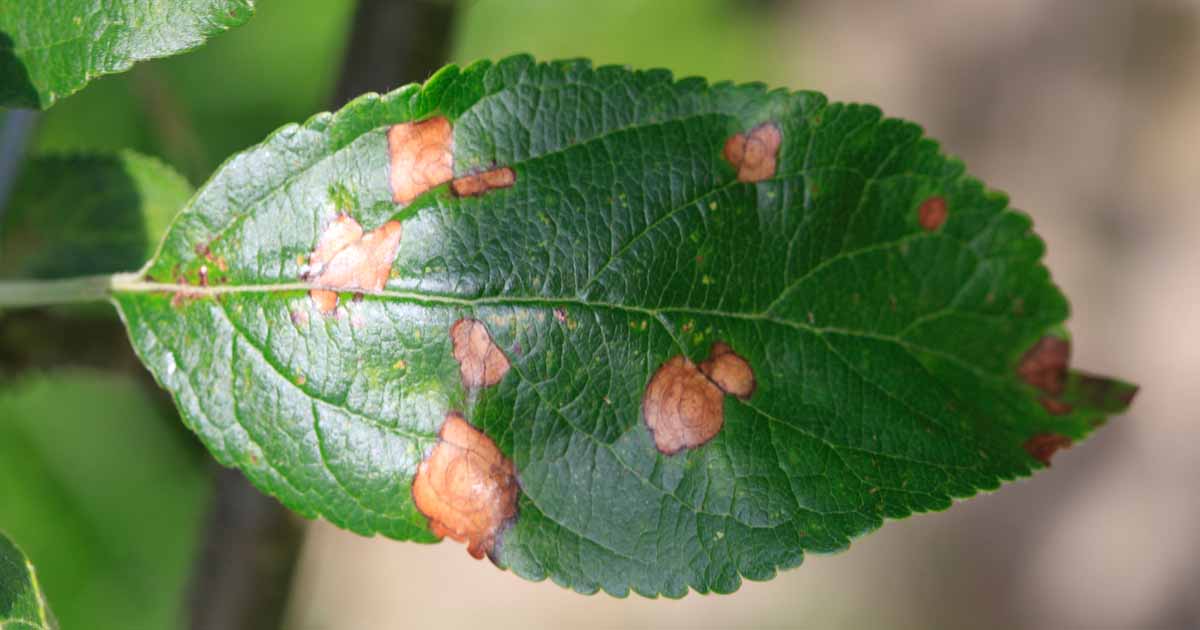

Supplement: Supplementary file 3 [file Data_Sheet_3.ZIP › test_orchard_1/17.jpg]

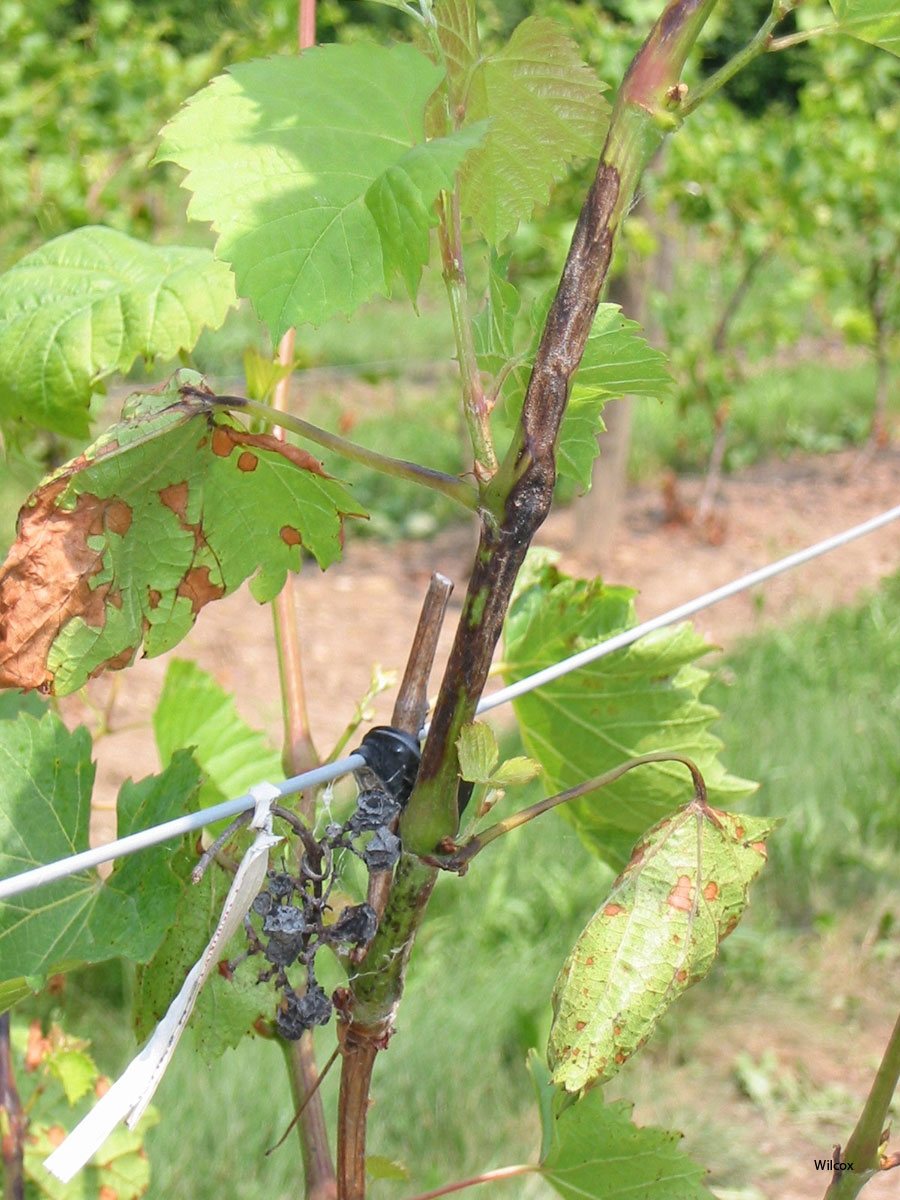

Supplement: Supplementary file 3 [file Data_Sheet_3.ZIP › test_orchard_1/18.jpg]

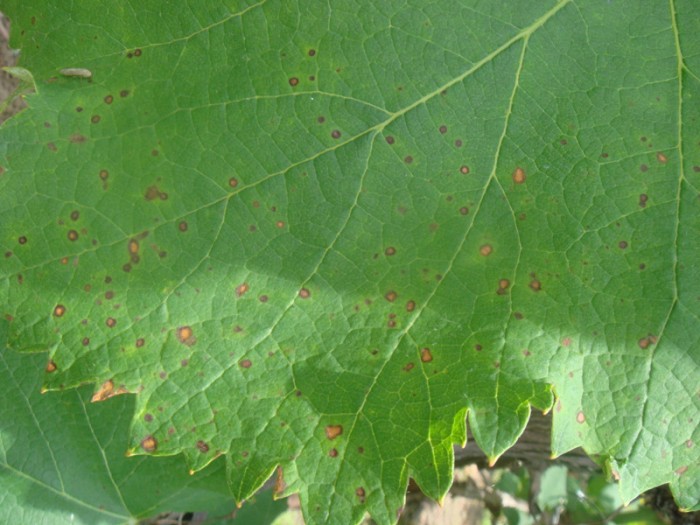

Supplement: Supplementary file 3 [file Data_Sheet_3.ZIP › test_orchard_1/19.jpg]

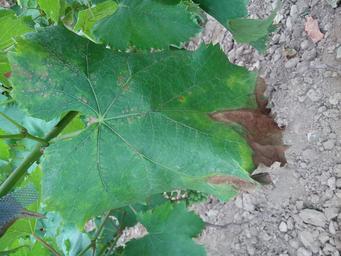

Supplement: Supplementary file 3 [file Data_Sheet_3.ZIP › test_orchard_1/20.jpg]

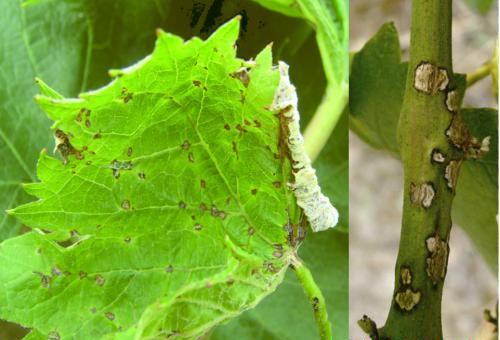

Supplement: Supplementary file 3 [file Data_Sheet_3.ZIP › test_orchard_1/21.png]

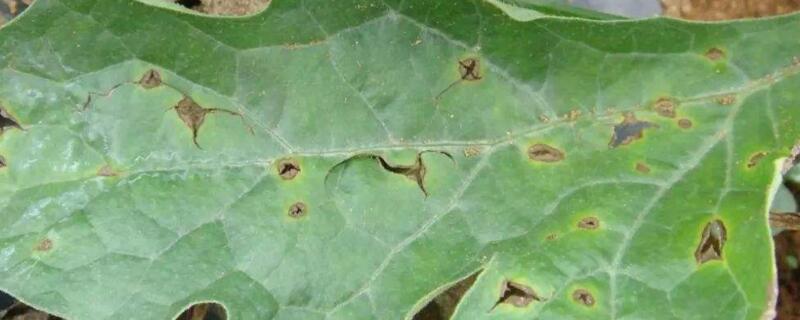

Supplement: Supplementary file 3 [file Data_Sheet_3.ZIP › test_orchard_1/22.jpg]

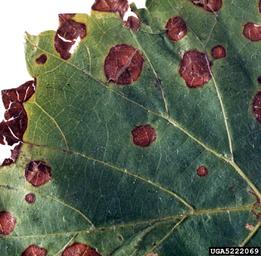

Supplement: Supplementary file 3 [file Data_Sheet_3.ZIP › test_orchard_1/23.jpg]

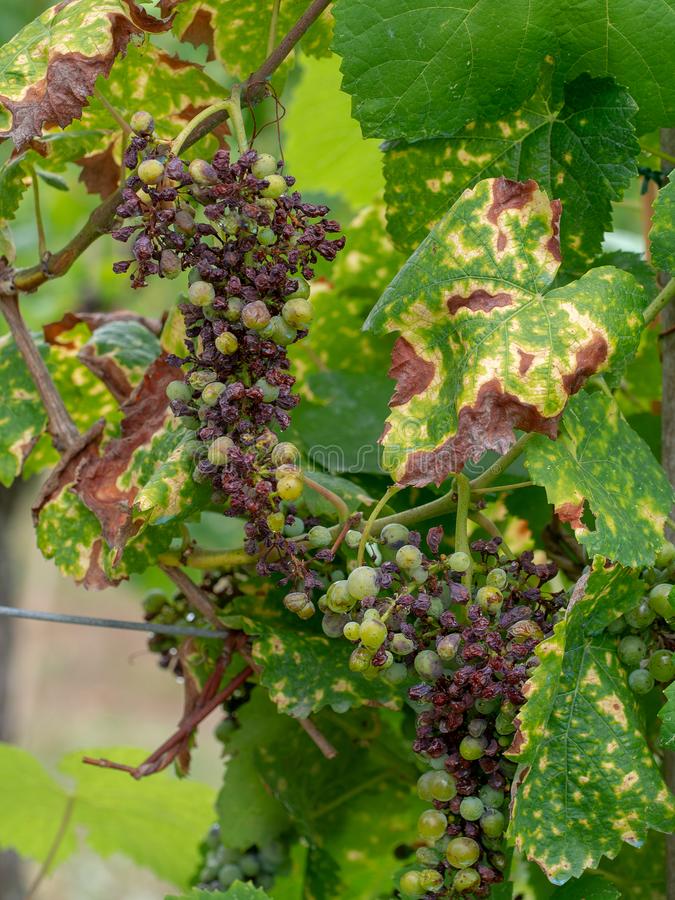

Supplement: Supplementary file 3 [file Data_Sheet_3.ZIP › test_orchard_1/24.jpg]

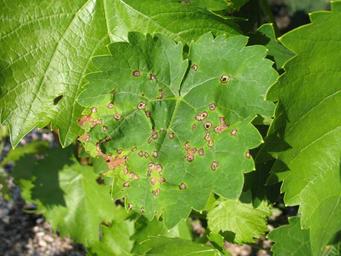

Supplement: Supplementary file 3 [file Data_Sheet_3.ZIP › test_orchard_1/25.jpg]

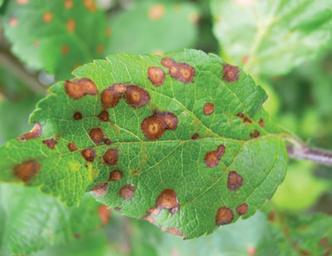

Supplement: Supplementary file 3 [file Data_Sheet_3.ZIP › test_orchard_1/26.jpg]

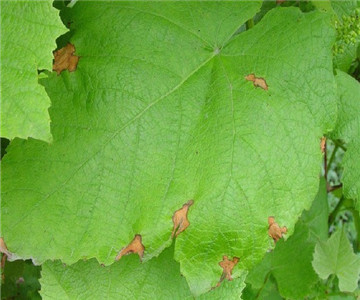

Supplement: Supplementary file 3 [file Data_Sheet_3.ZIP › test_orchard_1/27.jpg]

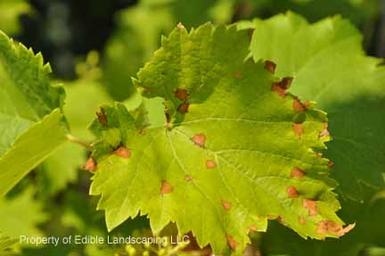

Supplement: Supplementary file 3 [file Data_Sheet_3.ZIP › test_orchard_1/28.jpg]

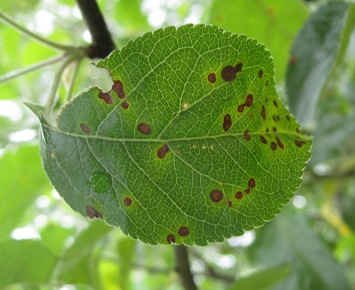

Supplement: Supplementary file 3 [file Data_Sheet_3.ZIP › test_orchard_1/29.jpg]

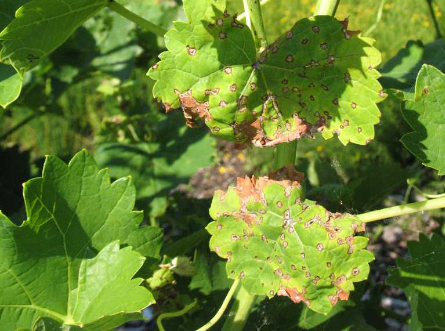

Supplement: Supplementary file 3 [file Data_Sheet_3.ZIP › test_orchard_1/30.png]

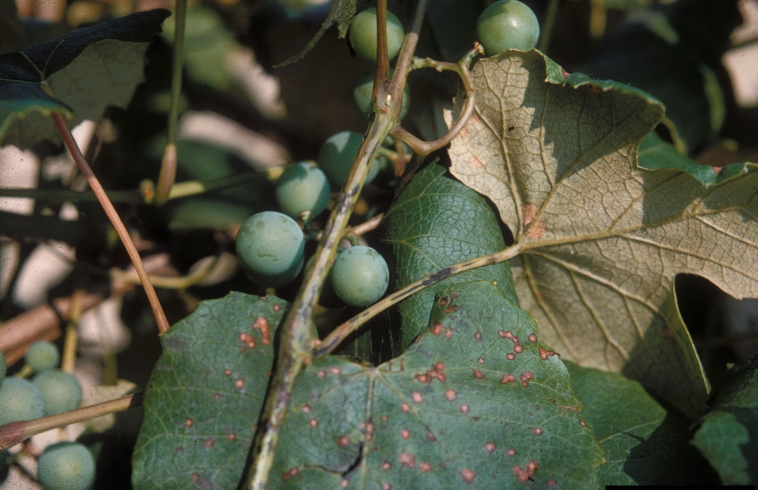

Supplement: Supplementary file 3 [file Data_Sheet_3.ZIP › test_orchard_1/31.png]

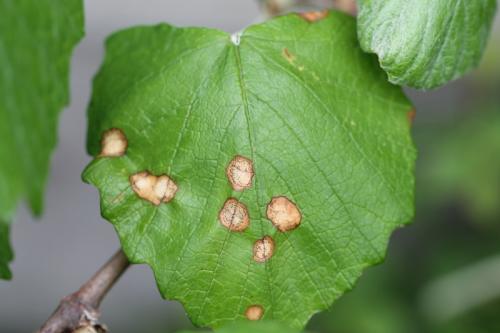

Supplement: Supplementary file 3 [file Data_Sheet_3.ZIP › test_orchard_1/32.jpg]

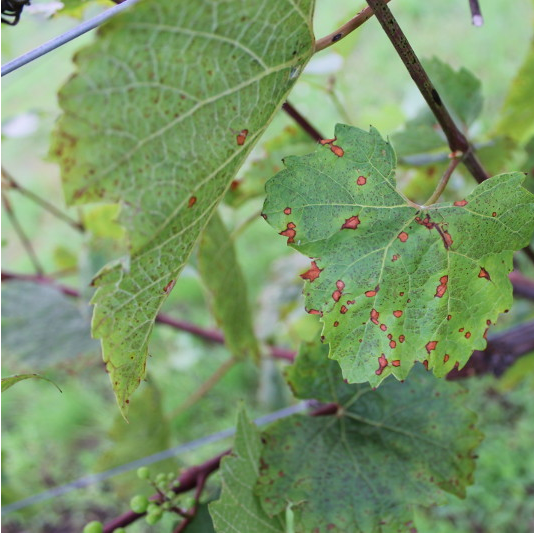

Supplement: Supplementary file 3 [file Data_Sheet_3.ZIP › test_orchard_1/33.png]

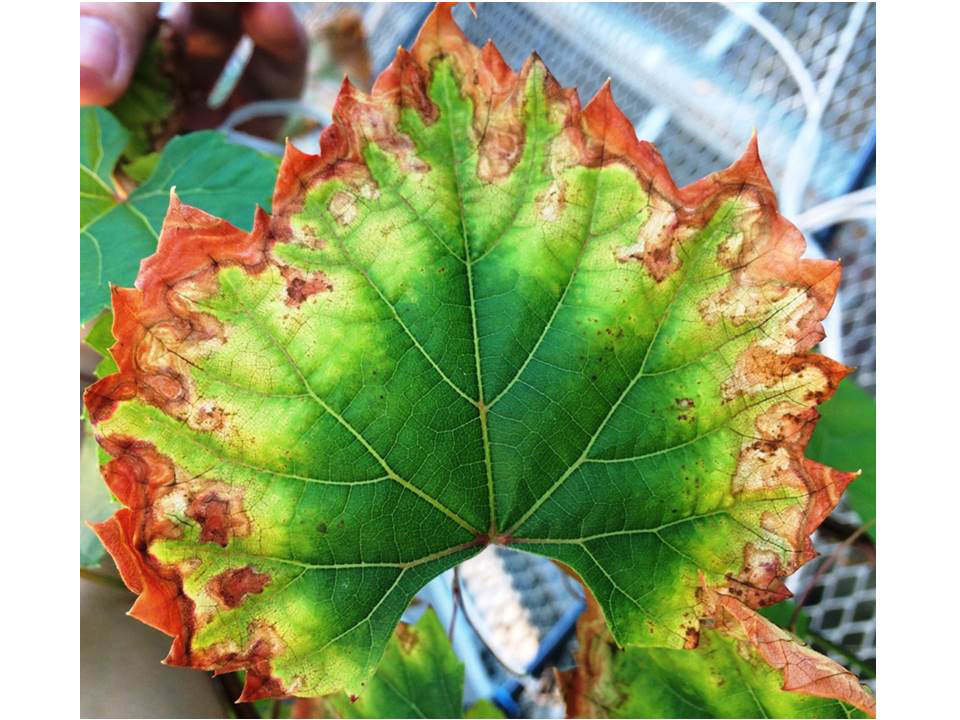

Supplement: Supplementary file 3 [file Data_Sheet_3.ZIP › test_orchard_1/34.jpg]

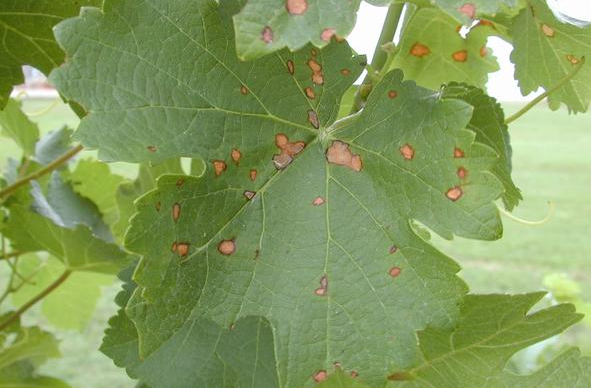

Supplement: Supplementary file 3 [file Data_Sheet_3.ZIP › test_orchard_1/35.png]

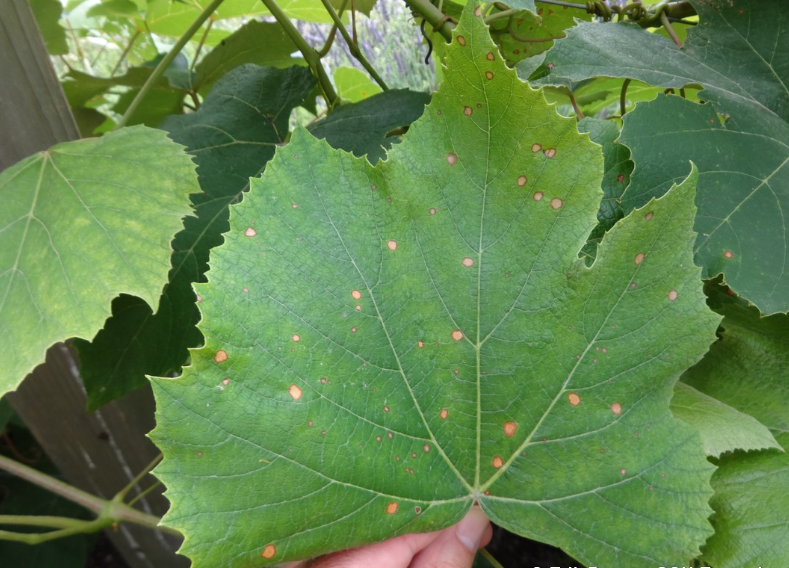

Supplement: Supplementary file 3 [file Data_Sheet_3.ZIP › test_orchard_1/36.png]

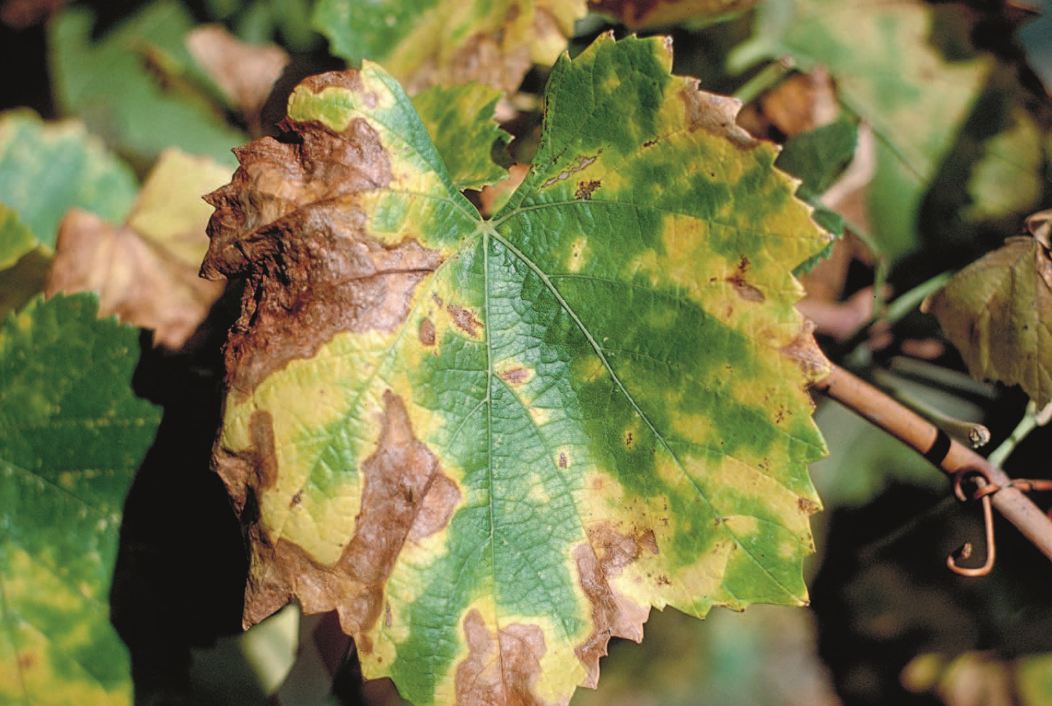

Supplement: Supplementary file 3 [file Data_Sheet_3.ZIP › test_orchard_1/37.jpg]

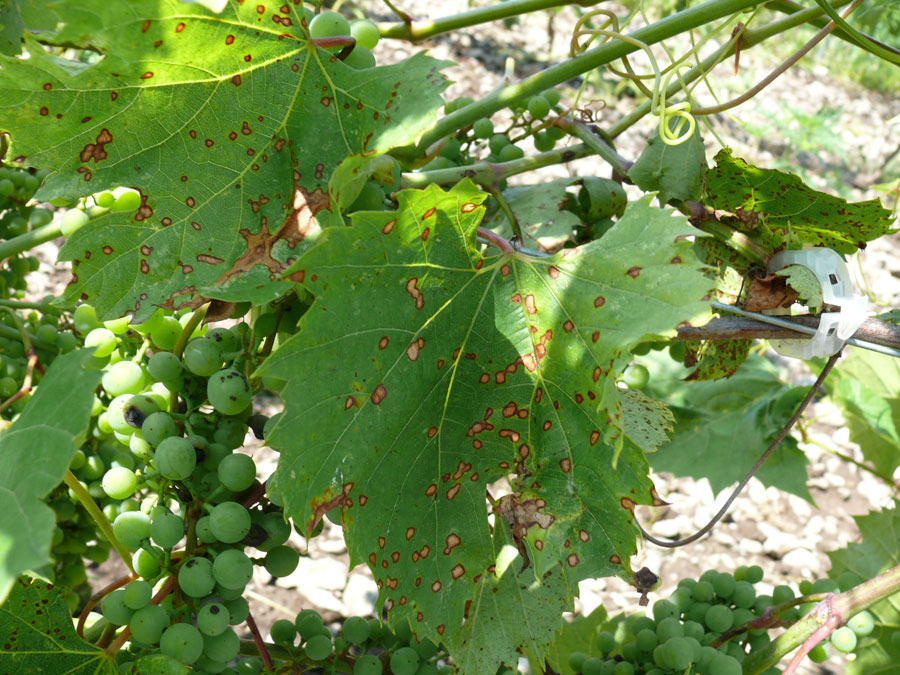

Supplement: Supplementary file 3 [file Data_Sheet_3.ZIP › test_orchard_1/38.jpg]

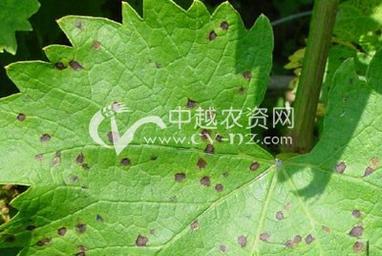

Supplement: Supplementary file 3 [file Data_Sheet_3.ZIP › test_orchard_1/40.jpg]

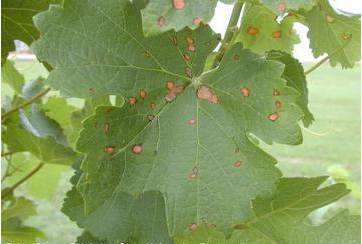

Supplement: Supplementary file 3 [file Data_Sheet_3.ZIP › test_orchard_1/41.png]

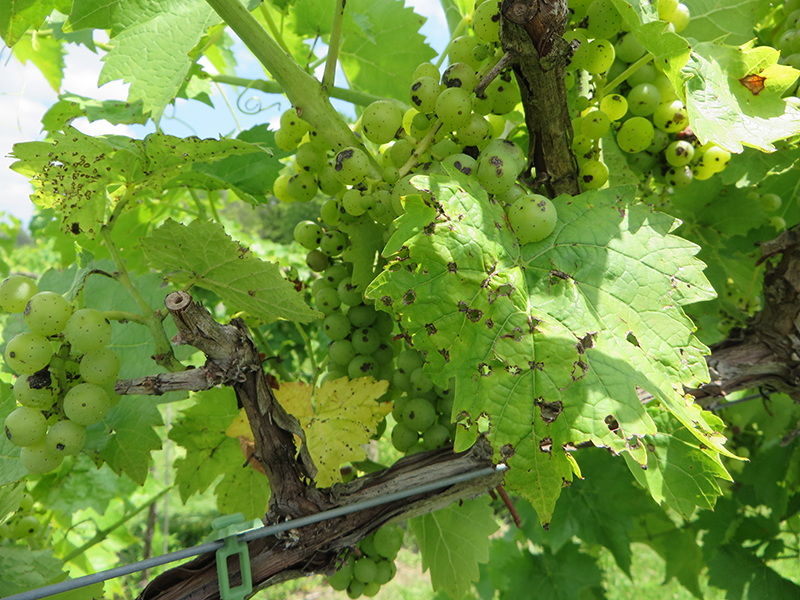

Supplement: Supplementary file 3 [file Data_Sheet_3.ZIP › test_orchard_1/42.jpg]

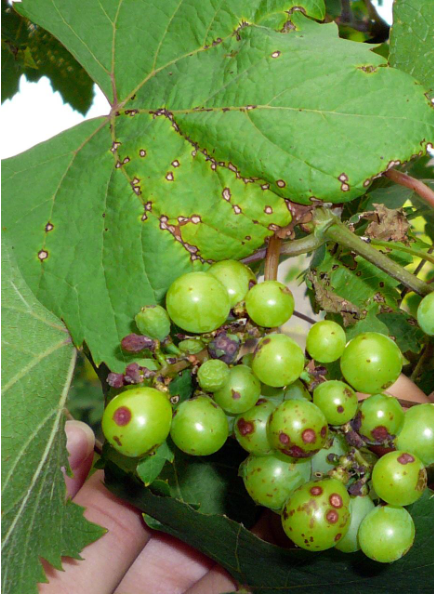

Supplement: Supplementary file 3 [file Data_Sheet_3.ZIP › test_orchard_1/43.png]

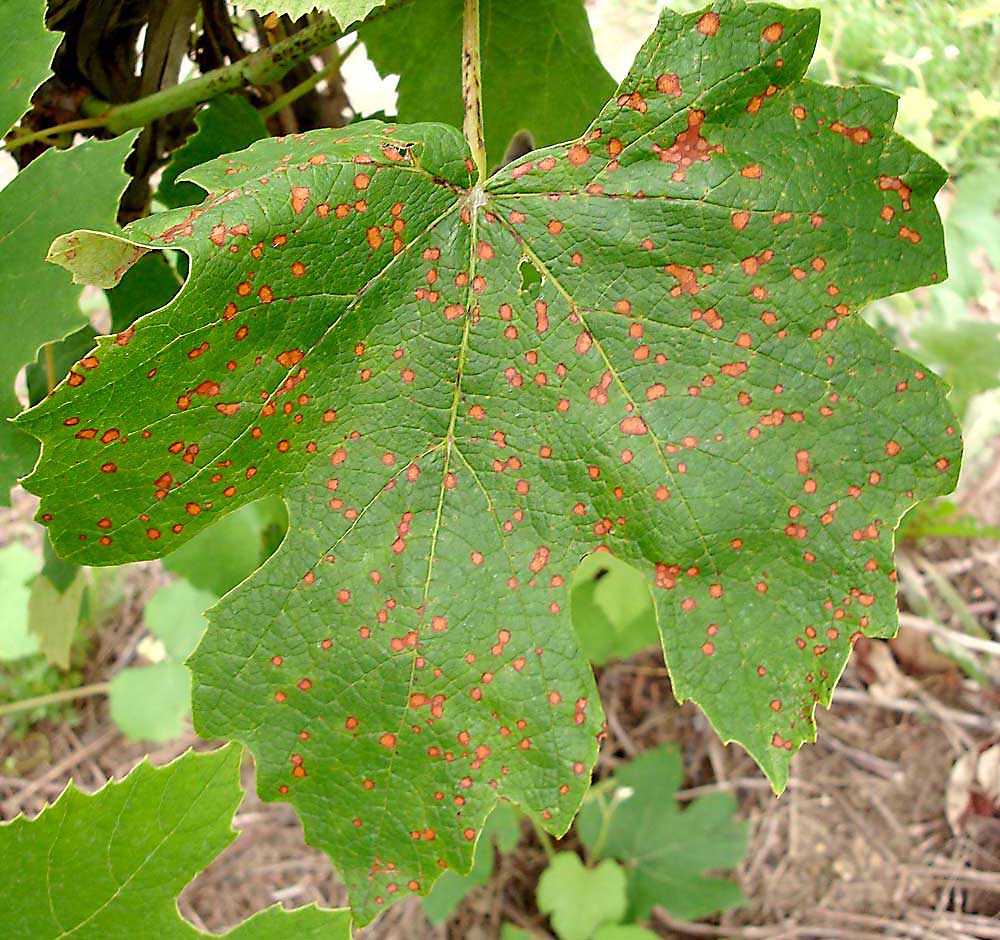

Supplement: Supplementary file 3 [file Data_Sheet_3.ZIP › test_orchard_1/44.jpg]

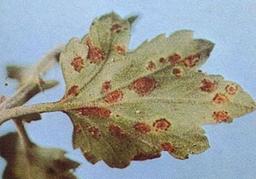

Supplement: Supplementary file 3 [file Data_Sheet_3.ZIP › test_orchard_1/45.jpg]

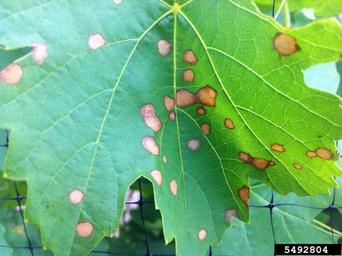

Supplement: Supplementary file 3 [file Data_Sheet_3.ZIP › test_orchard_1/46.jpg]

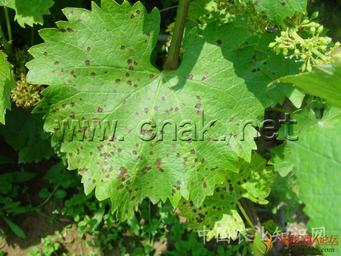

Supplement: Supplementary file 3 [file Data_Sheet_3.ZIP › test_orchard_1/47.jpg]

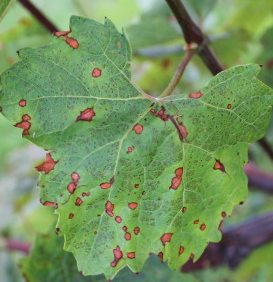

Supplement: Supplementary file 3 [file Data_Sheet_3.ZIP › test_orchard_1/48.png]

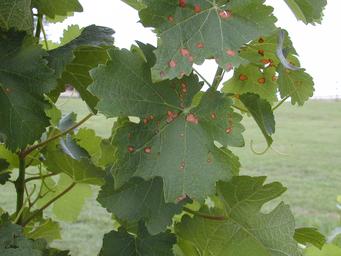

Supplement: Supplementary file 3 [file Data_Sheet_3.ZIP › test_orchard_1/49.jpg]

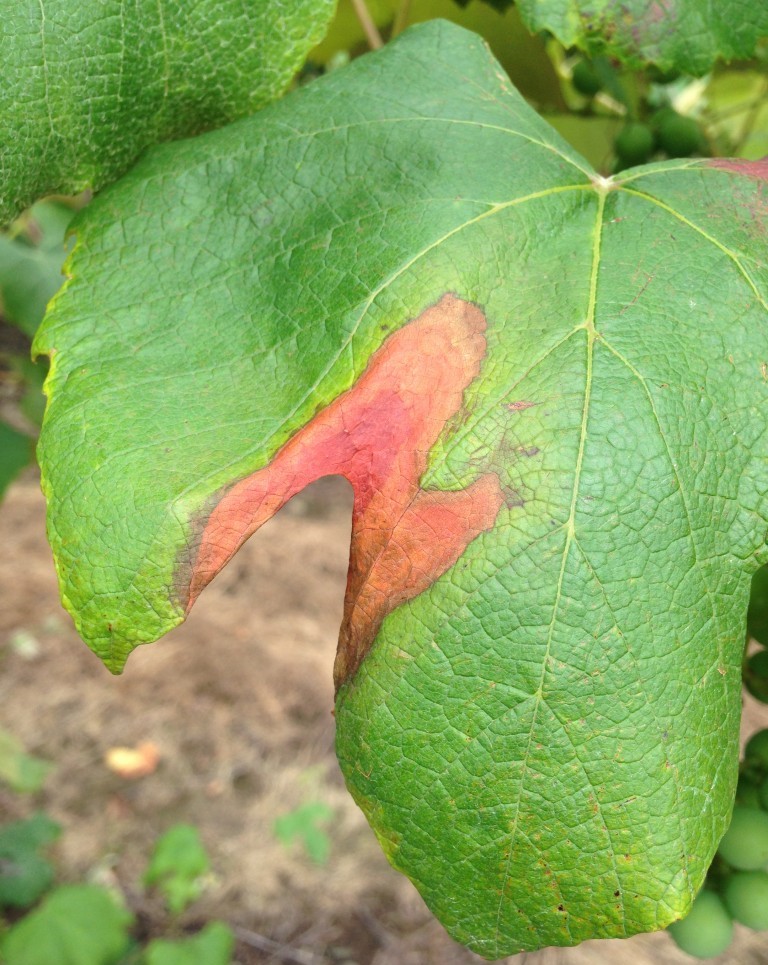

Supplement: Supplementary file 3 [file Data_Sheet_3.ZIP › test_orchard_1/50.jpg]

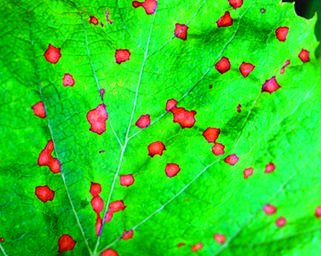

Supplement: Supplementary file 3 [file Data_Sheet_3.ZIP › test_orchard_1/51.jpg]

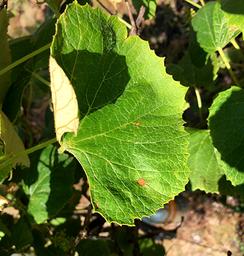

Supplement: Supplementary file 3 [file Data_Sheet_3.ZIP › test_orchard_1/52.jpg]

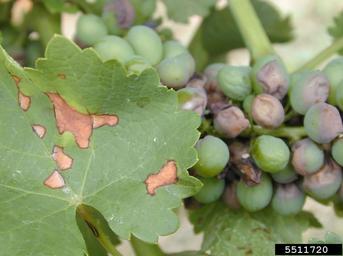

Supplement: Supplementary file 3 [file Data_Sheet_3.ZIP › test_orchard_1/53.jpg]

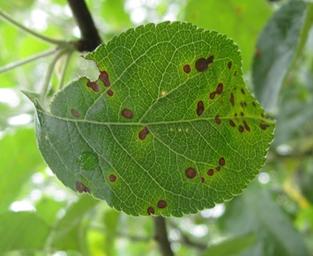

Supplement: Supplementary file 3 [file Data_Sheet_3.ZIP › test_orchard_1/54.jpg]

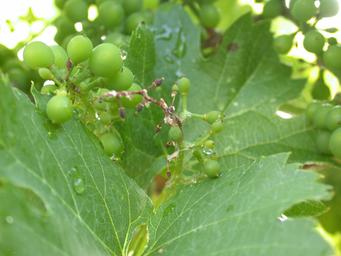

Supplement: Supplementary file 3 [file Data_Sheet_3.ZIP › test_orchard_1/55.jpg]

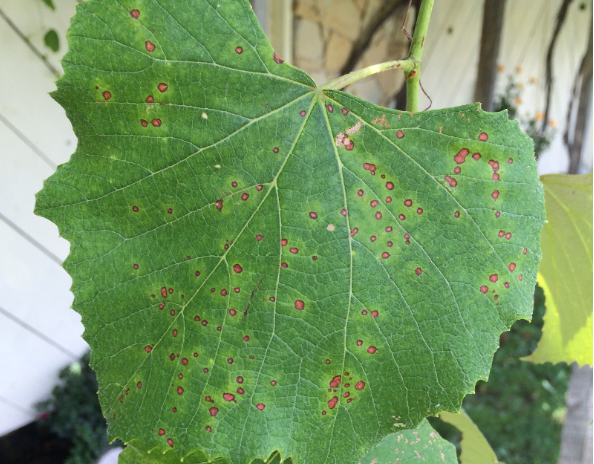

Supplement: Supplementary file 3 [file Data_Sheet_3.ZIP › test_orchard_1/55.png]

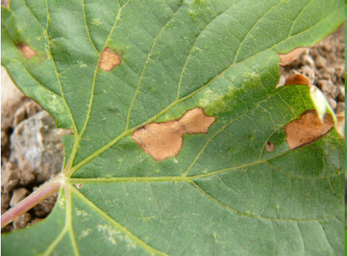

Supplement: Supplementary file 3 [file Data_Sheet_3.ZIP › test_orchard_1/56.png]

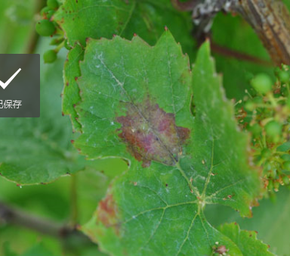

Supplement: Supplementary file 3 [file Data_Sheet_3.ZIP › test_orchard_1/57.png]

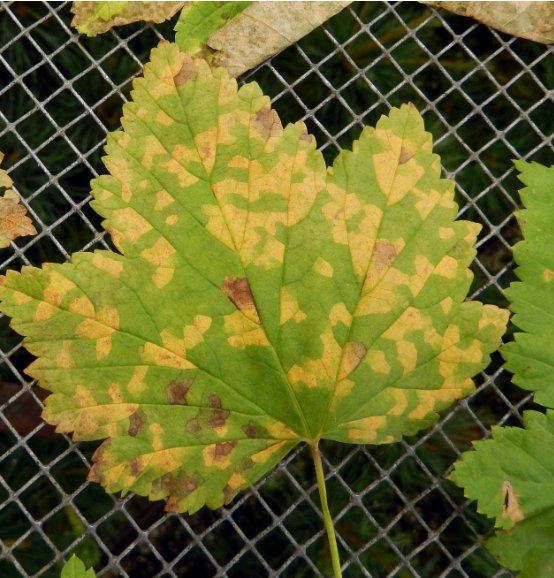

Supplement: Supplementary file 3 [file Data_Sheet_3.ZIP › test_orchard_1/58.png]

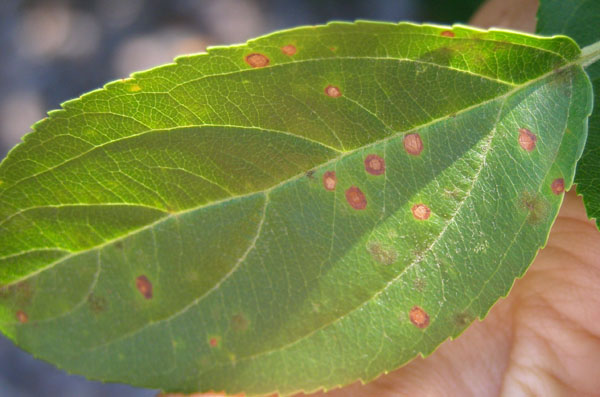

Supplement: Supplementary file 3 [file Data_Sheet_3.ZIP › test_orchard_1/60.jpg]

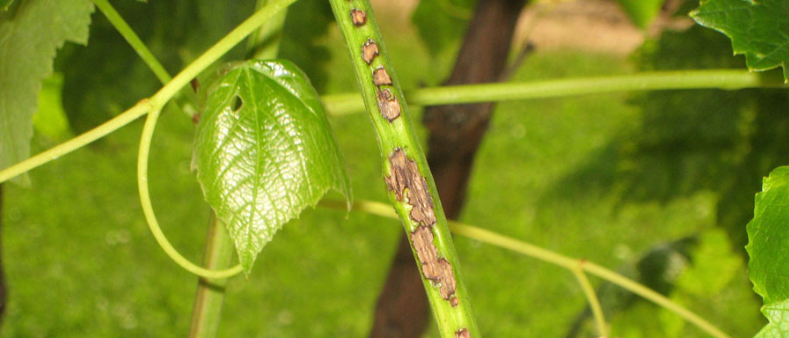

Supplement: Supplementary file 3 [file Data_Sheet_3.ZIP › test_orchard_1/61.png]

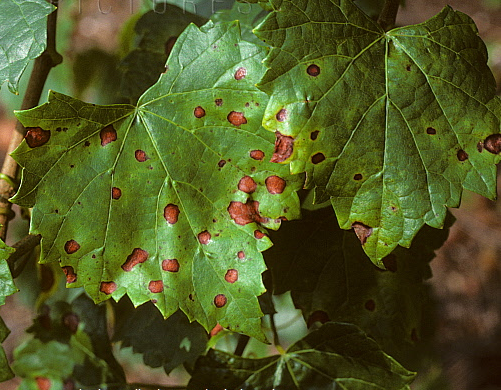

Supplement: Supplementary file 3 [file Data_Sheet_3.ZIP › test_orchard_1/62.png]

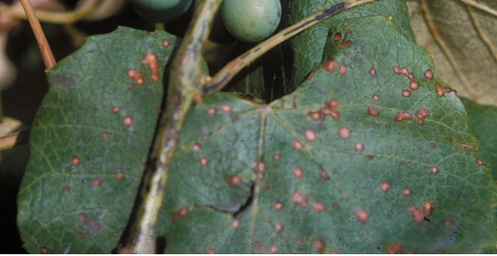

Supplement: Supplementary file 3 [file Data_Sheet_3.ZIP › test_orchard_1/63.png]

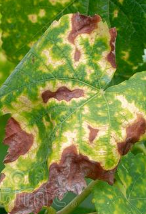

Supplement: Supplementary file 3 [file Data_Sheet_3.ZIP › test_orchard_1/65.png]

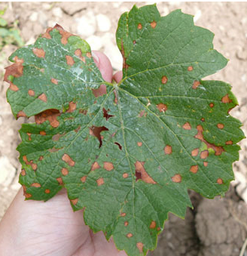

Supplement: Supplementary file 3 [file Data_Sheet_3.ZIP › test_orchard_1/66.png]

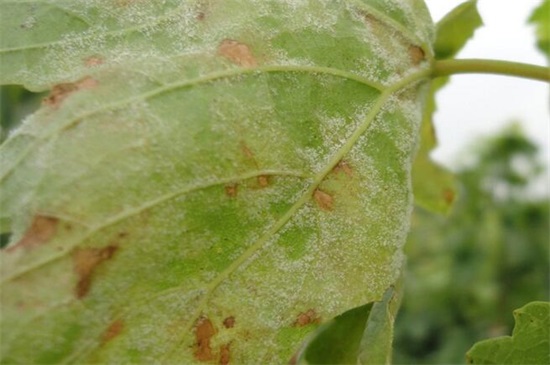

Supplement: Supplementary file 3 [file Data_Sheet_3.ZIP › test_orchard_1/67.jpg]

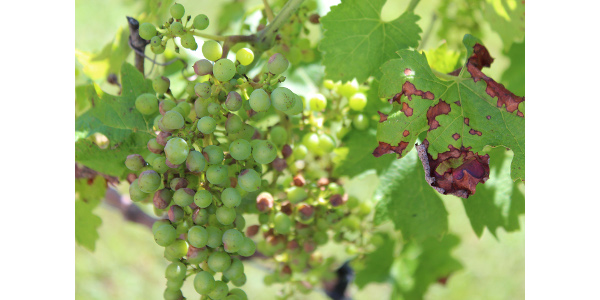

Supplement: Supplementary file 4 [file Data_Sheet_4.ZIP › test_orchard_2/68.jpg]

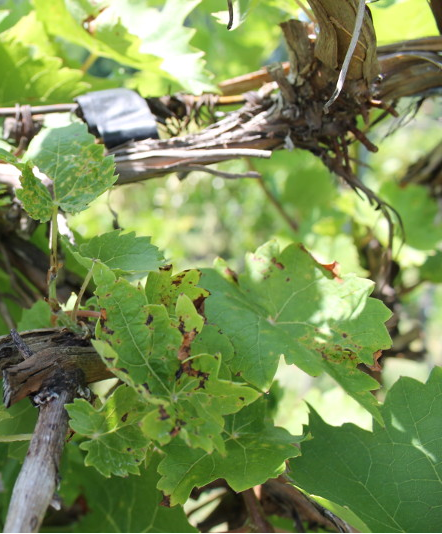

Supplement: Supplementary file 4 [file Data_Sheet_4.ZIP › test_orchard_2/69.png]

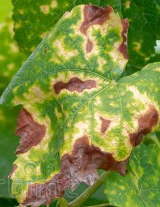

Supplement: Supplementary file 4 [file Data_Sheet_4.ZIP › test_orchard_2/70.png]

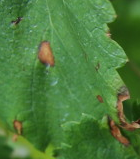

Supplement: Supplementary file 4 [file Data_Sheet_4.ZIP › test_orchard_2/71.png]

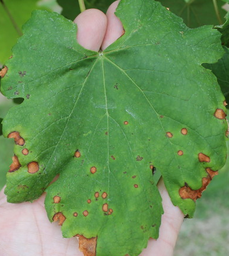

Supplement: Supplementary file 4 [file Data_Sheet_4.ZIP › test_orchard_2/72.png]

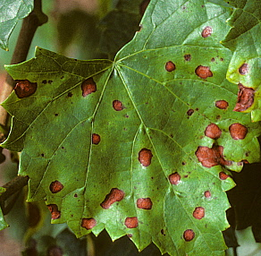

Supplement: Supplementary file 4 [file Data_Sheet_4.ZIP › test_orchard_2/73.png]

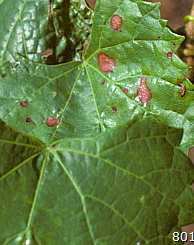

Supplement: Supplementary file 4 [file Data_Sheet_4.ZIP › test_orchard_2/74.png]

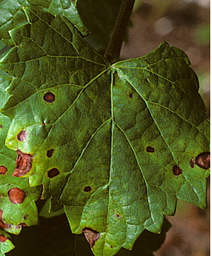

Supplement: Supplementary file 4 [file Data_Sheet_4.ZIP › test_orchard_2/75.png]

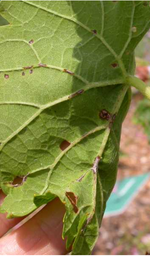

Supplement: Supplementary file 4 [file Data_Sheet_4.ZIP › test_orchard_2/76.png]

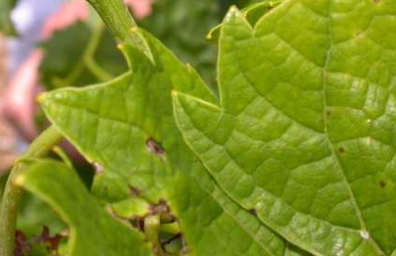

Supplement: Supplementary file 4 [file Data_Sheet_4.ZIP › test_orchard_2/77.png]

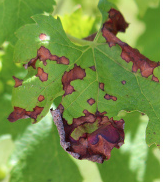

Supplement: Supplementary file 4 [file Data_Sheet_4.ZIP › test_orchard_2/78.png]

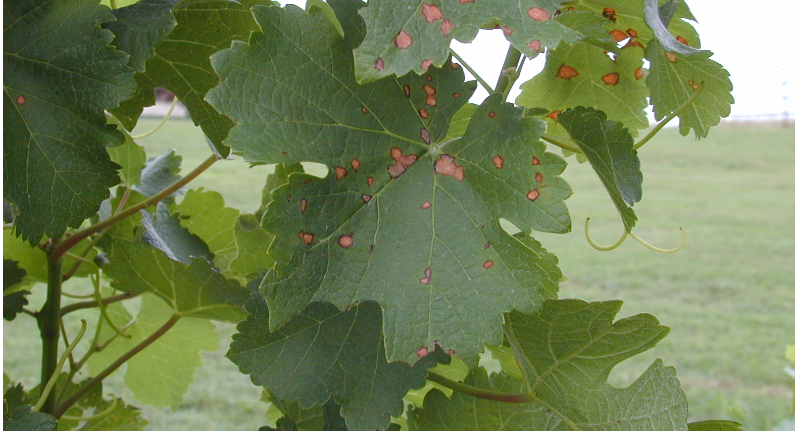

Supplement: Supplementary file 4 [file Data_Sheet_4.ZIP › test_orchard_2/79.png]

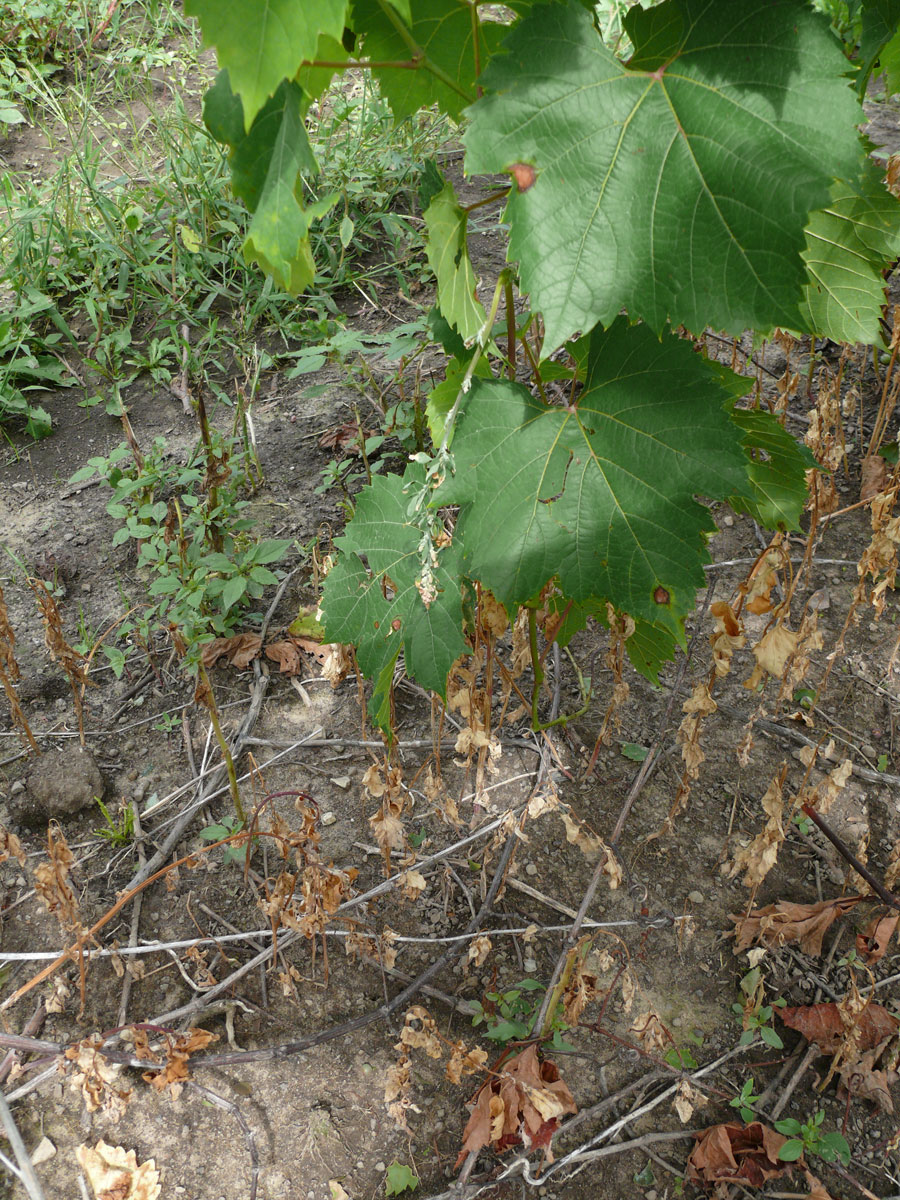

Supplement: Supplementary file 4 [file Data_Sheet_4.ZIP › test_orchard_2/80.jpg]

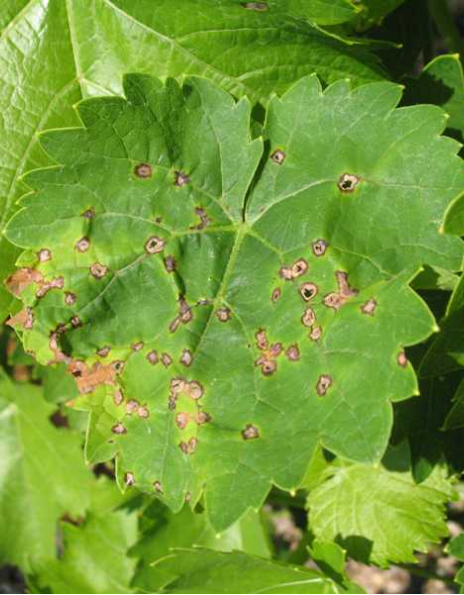

Supplement: Supplementary file 4 [file Data_Sheet_4.ZIP › test_orchard_2/81.png]

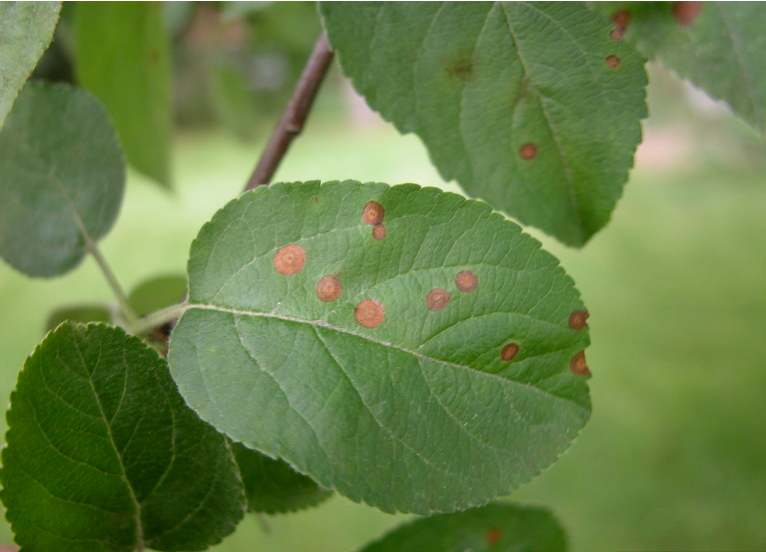

Supplement: Supplementary file 4 [file Data_Sheet_4.ZIP › test_orchard_2/82.png]

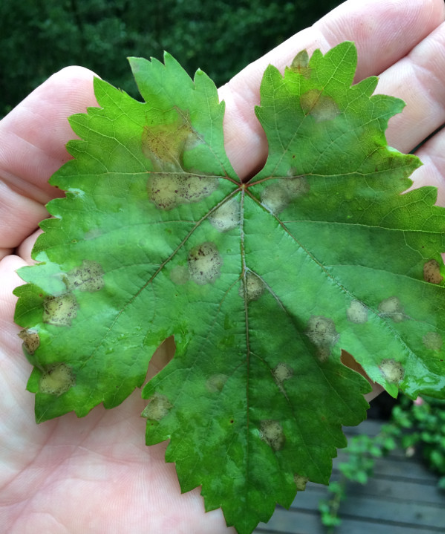

Supplement: Supplementary file 4 [file Data_Sheet_4.ZIP › test_orchard_2/83.png]

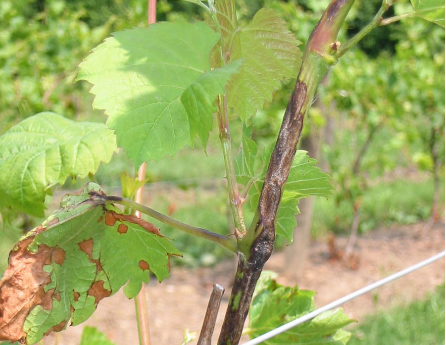

Supplement: Supplementary file 4 [file Data_Sheet_4.ZIP › test_orchard_2/84.png]

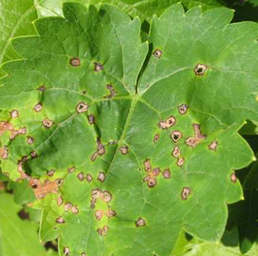

Supplement: Supplementary file 4 [file Data_Sheet_4.ZIP › test_orchard_2/85.png]

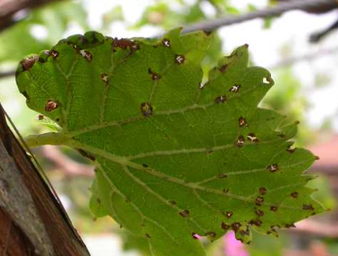

Supplement: Supplementary file 4 [file Data_Sheet_4.ZIP › test_orchard_2/86.png]

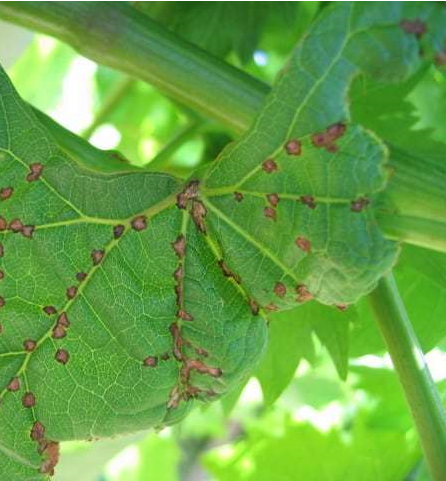

Supplement: Supplementary file 4 [file Data_Sheet_4.ZIP › test_orchard_2/88.png]

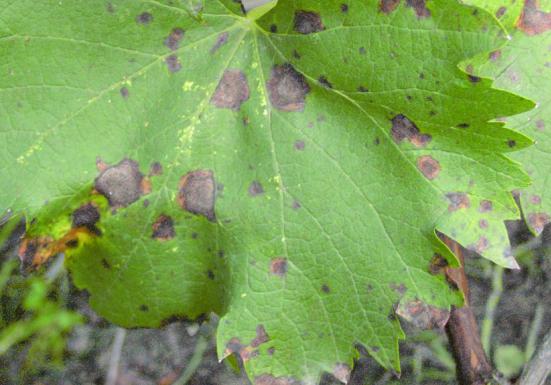

Supplement: Supplementary file 4 [file Data_Sheet_4.ZIP › test_orchard_2/89.jpg]

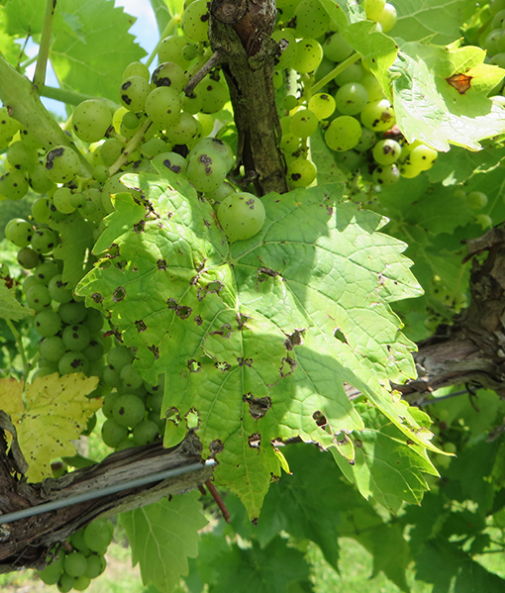

Supplement: Supplementary file 4 [file Data_Sheet_4.ZIP › test_orchard_2/90.png]

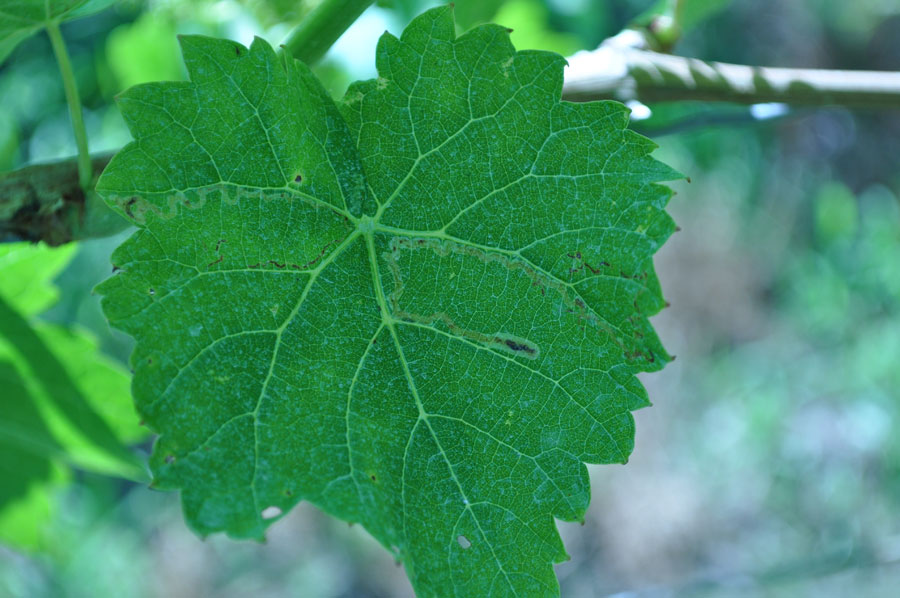

Supplement: Supplementary file 4 [file Data_Sheet_4.ZIP › test_orchard_2/91.jpg]

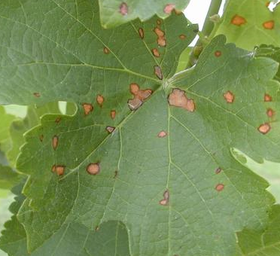

Supplement: Supplementary file 4 [file Data_Sheet_4.ZIP › test_orchard_2/92.png]

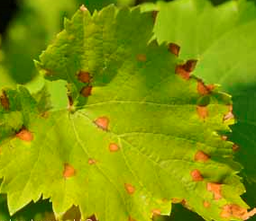

Supplement: Supplementary file 4 [file Data_Sheet_4.ZIP › test_orchard_2/93.png]

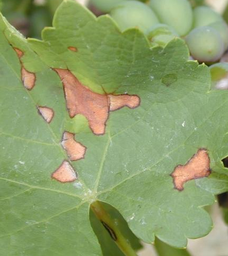

Supplement: Supplementary file 4 [file Data_Sheet_4.ZIP › test_orchard_2/94.png]

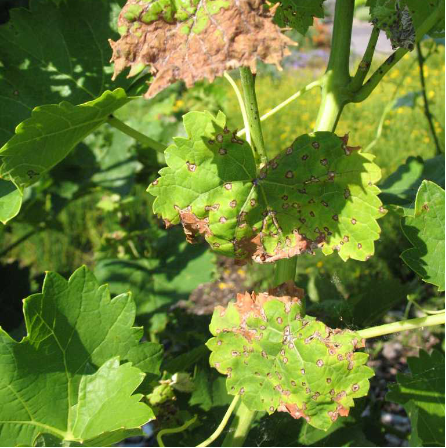

Supplement: Supplementary file 4 [file Data_Sheet_4.ZIP › test_orchard_2/95.png]

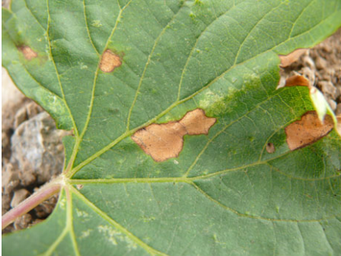

Supplement: Supplementary file 4 [file Data_Sheet_4.ZIP › test_orchard_2/96.png]

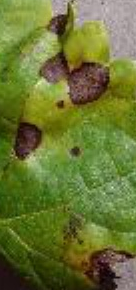

Supplement: Supplementary file 4 [file Data_Sheet_4.ZIP › test_orchard_2/97.png]

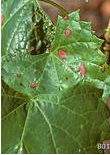

Supplement: Supplementary file 4 [file Data_Sheet_4.ZIP › test_orchard_2/98.png]

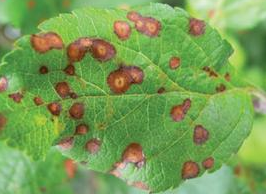

Supplement: Supplementary file 4 [file Data_Sheet_4.ZIP › test_orchard_2/99.png]

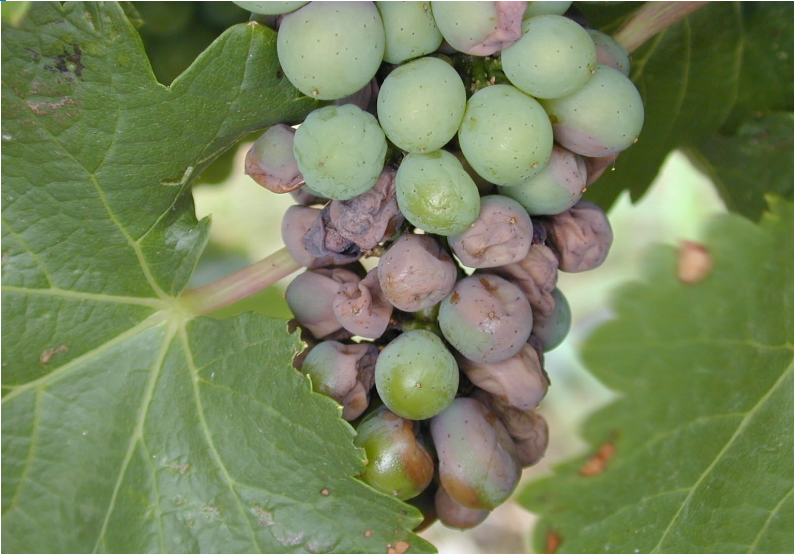

Supplement: Supplementary file 4 [file Data_Sheet_4.ZIP › test_orchard_2/100.png]

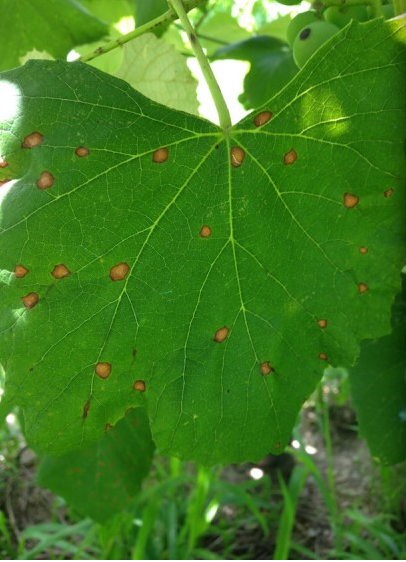

Supplement: Supplementary file 4 [file Data_Sheet_4.ZIP › test_orchard_2/101.png]

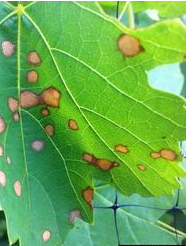

Supplement: Supplementary file 4 [file Data_Sheet_4.ZIP › test_orchard_2/102.png]

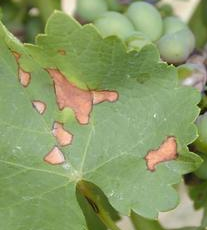

Supplement: Supplementary file 4 [file Data_Sheet_4.ZIP › test_orchard_2/103.png]
