# Supplementary figures and images for: Grape Leaf Black Rot Detection Based on Super-Resolution Image Enhancement and Deep Learning (part 5 of 6)
Source: Front Plant Sci. 2021 Jun 29;12:695749. doi: 10.3389/fpls.2021.695749 (PMC8277438; doi:10.3389/fpls.2021.695749)

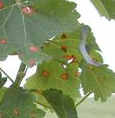

Supplement: Supplementary file 4 [file Data_Sheet_4.ZIP › test_orchard_2/105.png]

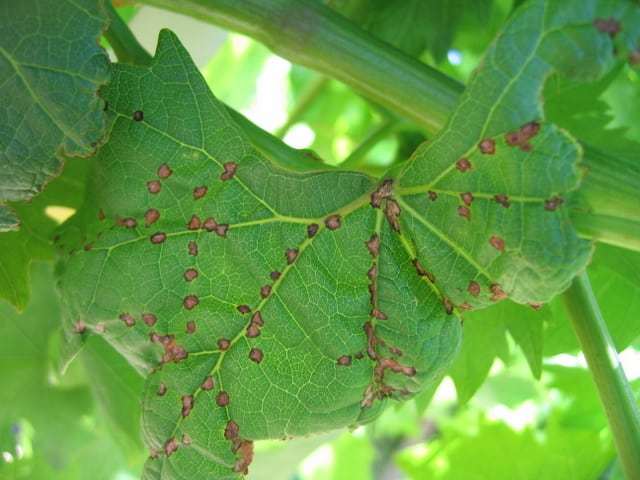

Supplement: Supplementary file 4 [file Data_Sheet_4.ZIP › test_orchard_2/106.jpg]

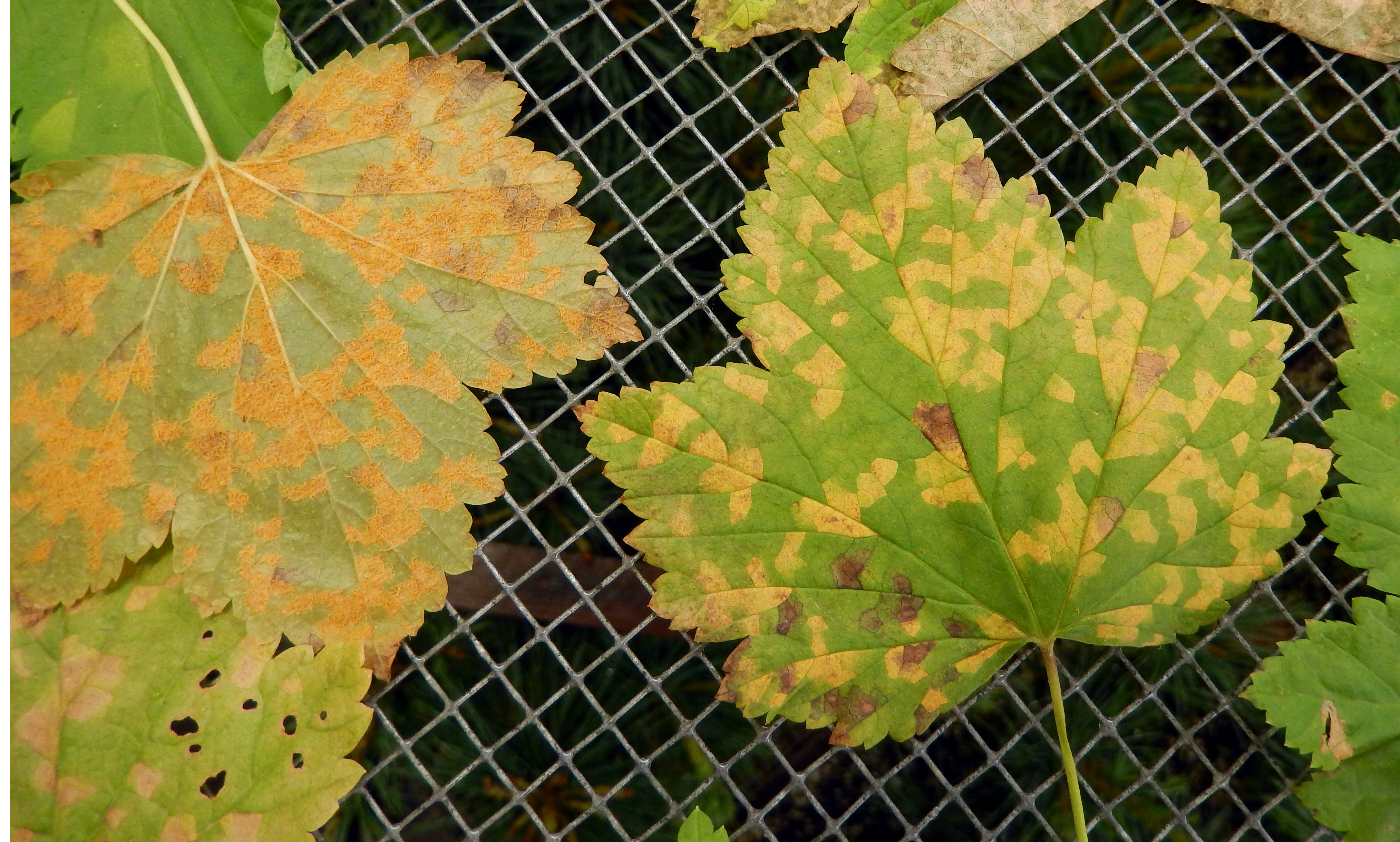

Supplement: Supplementary file 4 [file Data_Sheet_4.ZIP › test_orchard_2/107.jpg]

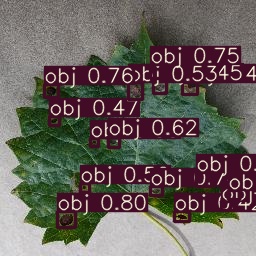

Supplement: Supplementary file 5 [file Data_Sheet_5.ZIP › code/output-bl-spp/0.JPG]

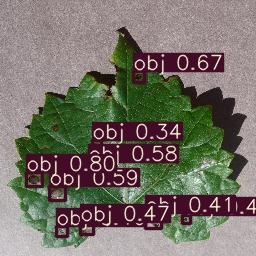

Supplement: Supplementary file 5 [file Data_Sheet_5.ZIP › code/output-bl-spp/1.JPG]

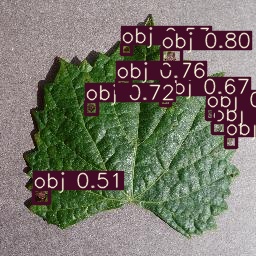

Supplement: Supplementary file 5 [file Data_Sheet_5.ZIP › code/output-bl-spp/10.JPG]

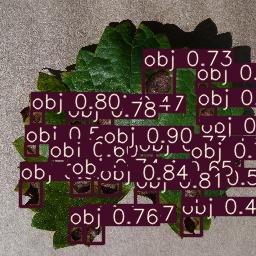

Supplement: Supplementary file 5 [file Data_Sheet_5.ZIP › code/output-bl-spp/100.JPG]

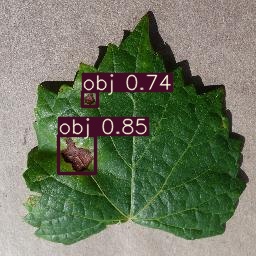

Supplement: Supplementary file 5 [file Data_Sheet_5.ZIP › code/output-bl-spp/101.JPG]

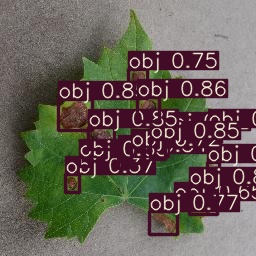

Supplement: Supplementary file 5 [file Data_Sheet_5.ZIP › code/output-bl-spp/102.JPG]

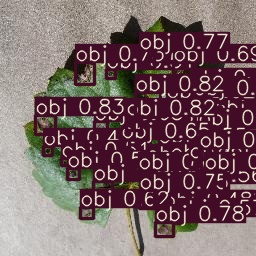

Supplement: Supplementary file 5 [file Data_Sheet_5.ZIP › code/output-bl-spp/103.JPG]

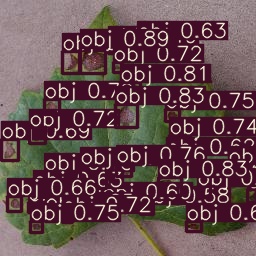

Supplement: Supplementary file 5 [file Data_Sheet_5.ZIP › code/output-bl-spp/104.JPG]

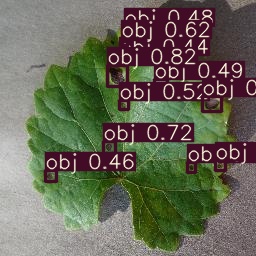

Supplement: Supplementary file 5 [file Data_Sheet_5.ZIP › code/output-bl-spp/105.JPG]

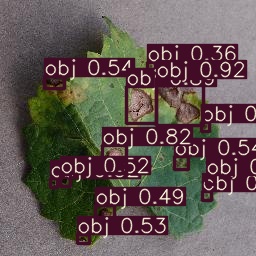

Supplement: Supplementary file 5 [file Data_Sheet_5.ZIP › code/output-bl-spp/106.JPG]

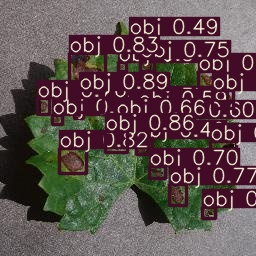

Supplement: Supplementary file 5 [file Data_Sheet_5.ZIP › code/output-bl-spp/107.JPG]

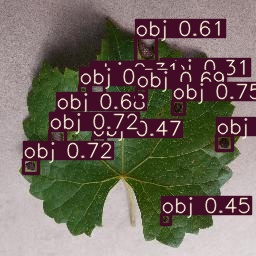

Supplement: Supplementary file 5 [file Data_Sheet_5.ZIP › code/output-bl-spp/11.JPG]

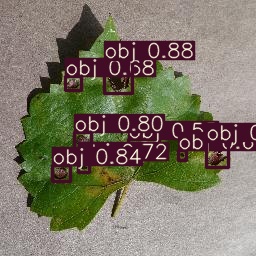

Supplement: Supplementary file 5 [file Data_Sheet_5.ZIP › code/output-bl-spp/12.JPG]

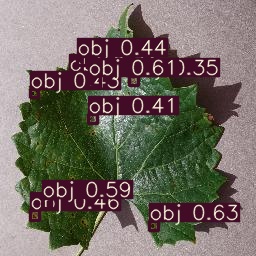

Supplement: Supplementary file 5 [file Data_Sheet_5.ZIP › code/output-bl-spp/13.JPG]

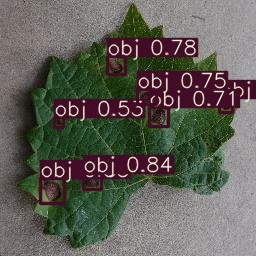

Supplement: Supplementary file 5 [file Data_Sheet_5.ZIP › code/output-bl-spp/14.JPG]

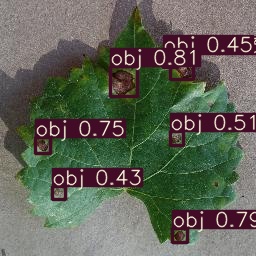

Supplement: Supplementary file 5 [file Data_Sheet_5.ZIP › code/output-bl-spp/15.JPG]

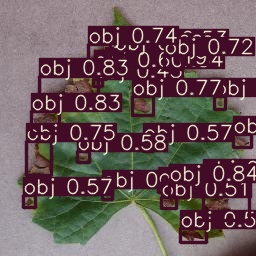

Supplement: Supplementary file 5 [file Data_Sheet_5.ZIP › code/output-bl-spp/16.JPG]

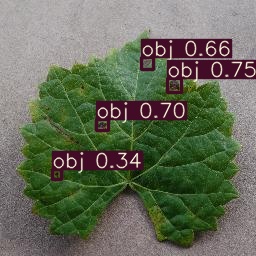

Supplement: Supplementary file 5 [file Data_Sheet_5.ZIP › code/output-bl-spp/17.JPG]

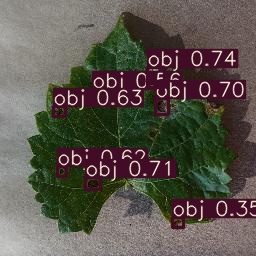

Supplement: Supplementary file 5 [file Data_Sheet_5.ZIP › code/output-bl-spp/18.JPG]

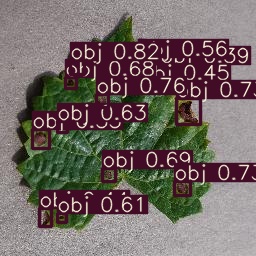

Supplement: Supplementary file 5 [file Data_Sheet_5.ZIP › code/output-bl-spp/19.JPG]

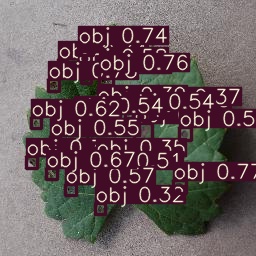

Supplement: Supplementary file 5 [file Data_Sheet_5.ZIP › code/output-bl-spp/2.JPG]

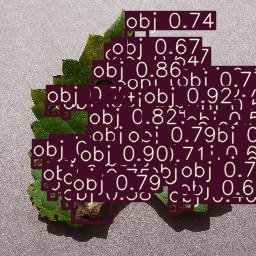

Supplement: Supplementary file 5 [file Data_Sheet_5.ZIP › code/output-bl-spp/20.JPG]

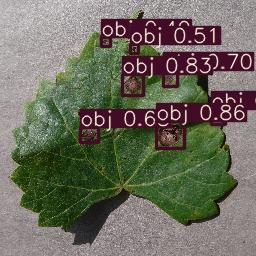

Supplement: Supplementary file 5 [file Data_Sheet_5.ZIP › code/output-bl-spp/21.JPG]

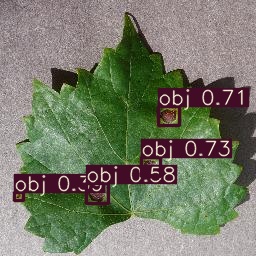

Supplement: Supplementary file 5 [file Data_Sheet_5.ZIP › code/output-bl-spp/22.JPG]

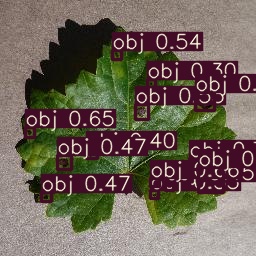

Supplement: Supplementary file 5 [file Data_Sheet_5.ZIP › code/output-bl-spp/23.JPG]

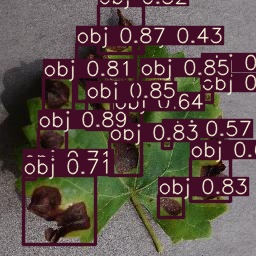

Supplement: Supplementary file 5 [file Data_Sheet_5.ZIP › code/output-bl-spp/24.JPG]

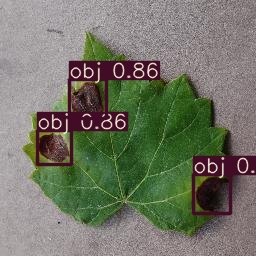

Supplement: Supplementary file 5 [file Data_Sheet_5.ZIP › code/output-bl-spp/25.JPG]

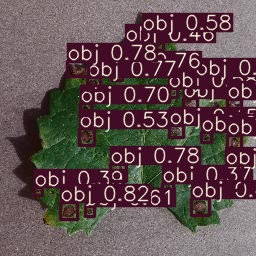

Supplement: Supplementary file 5 [file Data_Sheet_5.ZIP › code/output-bl-spp/26.JPG]

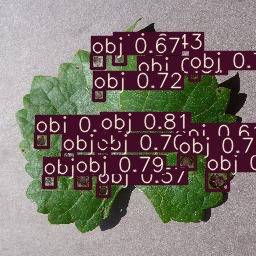

Supplement: Supplementary file 5 [file Data_Sheet_5.ZIP › code/output-bl-spp/27.JPG]

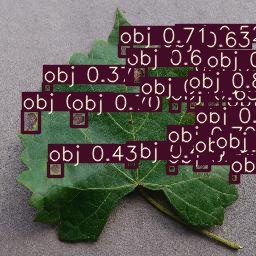

Supplement: Supplementary file 5 [file Data_Sheet_5.ZIP › code/output-bl-spp/28.JPG]

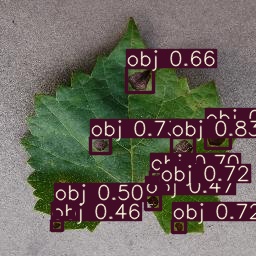

Supplement: Supplementary file 5 [file Data_Sheet_5.ZIP › code/output-bl-spp/29.JPG]

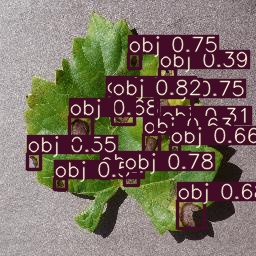

Supplement: Supplementary file 5 [file Data_Sheet_5.ZIP › code/output-bl-spp/3.JPG]

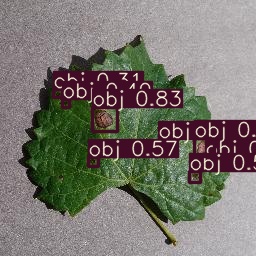

Supplement: Supplementary file 5 [file Data_Sheet_5.ZIP › code/output-bl-spp/30.JPG]

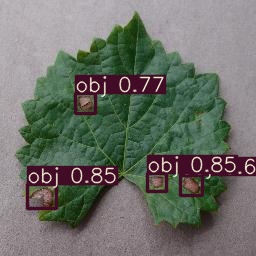

Supplement: Supplementary file 5 [file Data_Sheet_5.ZIP › code/output-bl-spp/31.JPG]

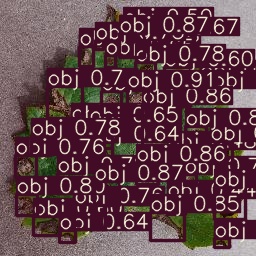

Supplement: Supplementary file 5 [file Data_Sheet_5.ZIP › code/output-bl-spp/32.JPG]

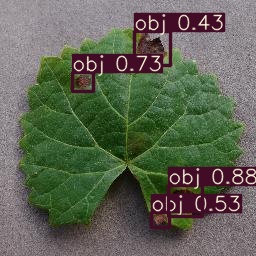

Supplement: Supplementary file 5 [file Data_Sheet_5.ZIP › code/output-bl-spp/33.JPG]

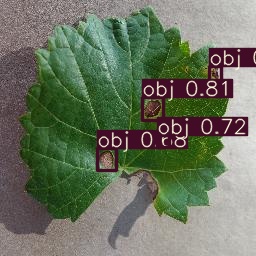

Supplement: Supplementary file 5 [file Data_Sheet_5.ZIP › code/output-bl-spp/34.JPG]

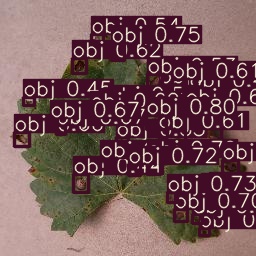

Supplement: Supplementary file 5 [file Data_Sheet_5.ZIP › code/output-bl-spp/35.JPG]

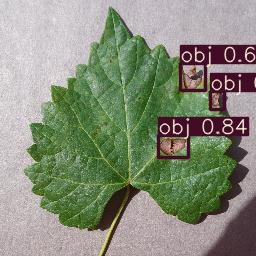

Supplement: Supplementary file 5 [file Data_Sheet_5.ZIP › code/output-bl-spp/36.JPG]

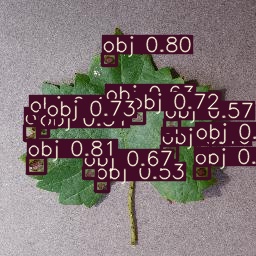

Supplement: Supplementary file 5 [file Data_Sheet_5.ZIP › code/output-bl-spp/37.JPG]

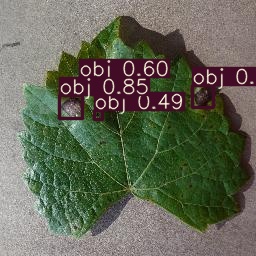

Supplement: Supplementary file 5 [file Data_Sheet_5.ZIP › code/output-bl-spp/38.JPG]

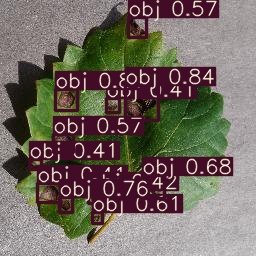

Supplement: Supplementary file 5 [file Data_Sheet_5.ZIP › code/output-bl-spp/39.JPG]

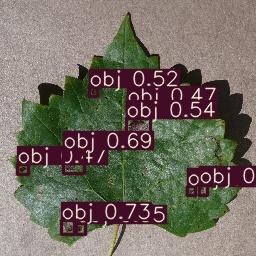

Supplement: Supplementary file 5 [file Data_Sheet_5.ZIP › code/output-bl-spp/4.JPG]

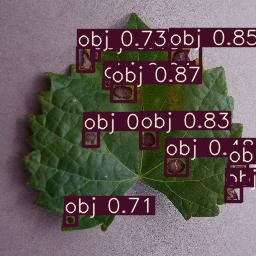

Supplement: Supplementary file 5 [file Data_Sheet_5.ZIP › code/output-bl-spp/40.JPG]

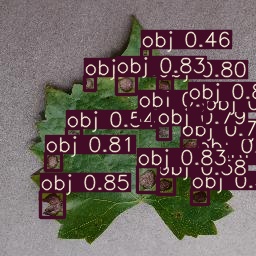

Supplement: Supplementary file 5 [file Data_Sheet_5.ZIP › code/output-bl-spp/41.JPG]

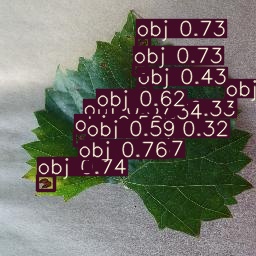

Supplement: Supplementary file 5 [file Data_Sheet_5.ZIP › code/output-bl-spp/42.JPG]

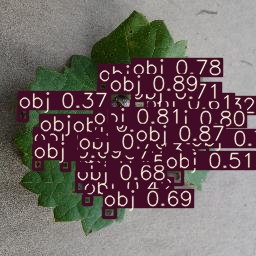

Supplement: Supplementary file 5 [file Data_Sheet_5.ZIP › code/output-bl-spp/43.JPG]

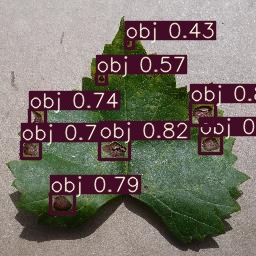

Supplement: Supplementary file 5 [file Data_Sheet_5.ZIP › code/output-bl-spp/44.JPG]

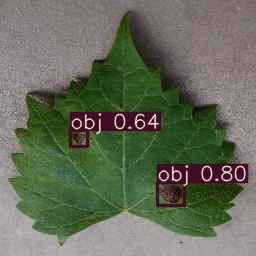

Supplement: Supplementary file 5 [file Data_Sheet_5.ZIP › code/output-bl-spp/45.JPG]

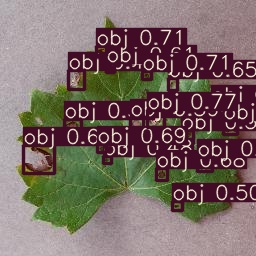

Supplement: Supplementary file 5 [file Data_Sheet_5.ZIP › code/output-bl-spp/46.JPG]

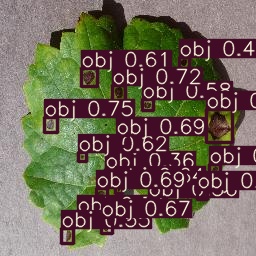

Supplement: Supplementary file 5 [file Data_Sheet_5.ZIP › code/output-bl-spp/47.JPG]

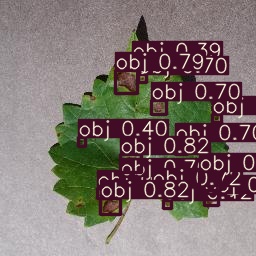

Supplement: Supplementary file 5 [file Data_Sheet_5.ZIP › code/output-bl-spp/48.JPG]

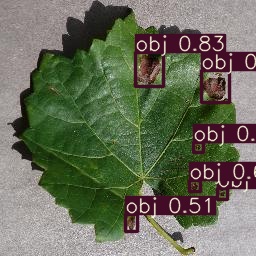

Supplement: Supplementary file 5 [file Data_Sheet_5.ZIP › code/output-bl-spp/49.JPG]

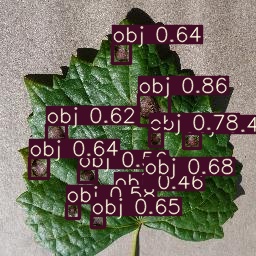

Supplement: Supplementary file 5 [file Data_Sheet_5.ZIP › code/output-bl-spp/5.JPG]

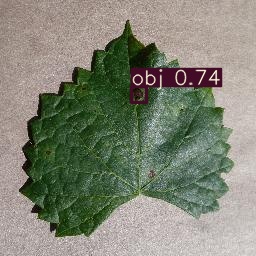

Supplement: Supplementary file 5 [file Data_Sheet_5.ZIP › code/output-bl-spp/50.JPG]

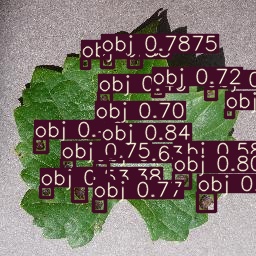

Supplement: Supplementary file 5 [file Data_Sheet_5.ZIP › code/output-bl-spp/51.JPG]

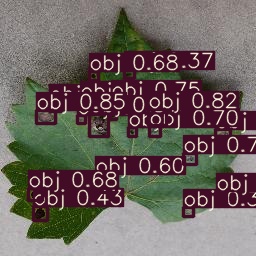

Supplement: Supplementary file 5 [file Data_Sheet_5.ZIP › code/output-bl-spp/52.JPG]

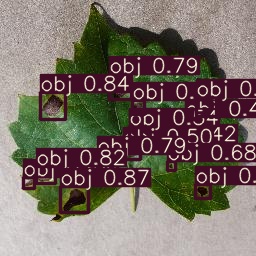

Supplement: Supplementary file 5 [file Data_Sheet_5.ZIP › code/output-bl-spp/53.JPG]

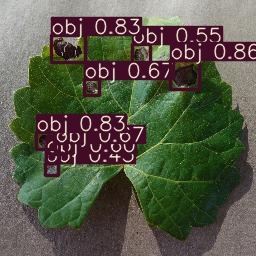

Supplement: Supplementary file 5 [file Data_Sheet_5.ZIP › code/output-bl-spp/54.JPG]

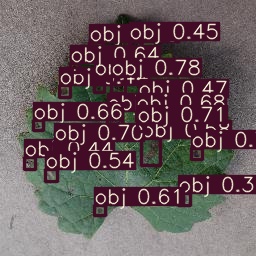

Supplement: Supplementary file 5 [file Data_Sheet_5.ZIP › code/output-bl-spp/55.JPG]

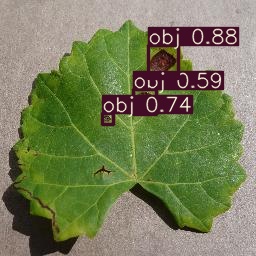

Supplement: Supplementary file 5 [file Data_Sheet_5.ZIP › code/output-bl-spp/56.JPG]

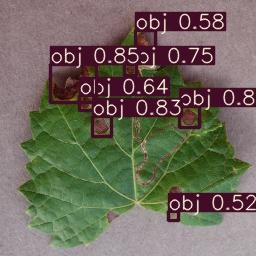

Supplement: Supplementary file 5 [file Data_Sheet_5.ZIP › code/output-bl-spp/57.JPG]

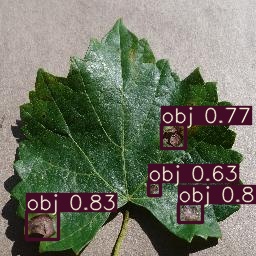

Supplement: Supplementary file 5 [file Data_Sheet_5.ZIP › code/output-bl-spp/58.JPG]

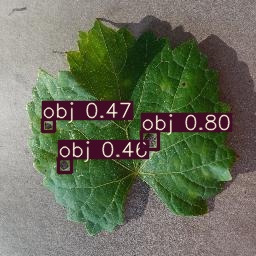

Supplement: Supplementary file 5 [file Data_Sheet_5.ZIP › code/output-bl-spp/59.JPG]

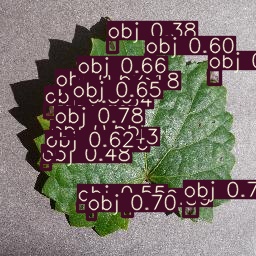

Supplement: Supplementary file 5 [file Data_Sheet_5.ZIP › code/output-bl-spp/6.JPG]

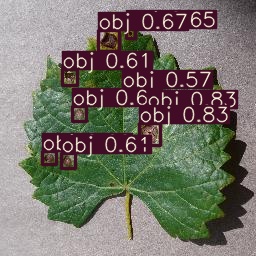

Supplement: Supplementary file 5 [file Data_Sheet_5.ZIP › code/output-bl-spp/60.JPG]

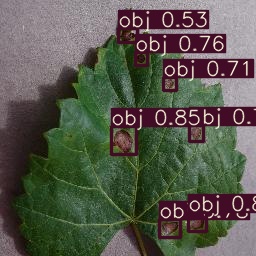

Supplement: Supplementary file 5 [file Data_Sheet_5.ZIP › code/output-bl-spp/61.JPG]

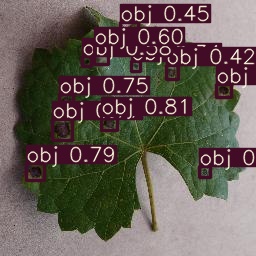

Supplement: Supplementary file 5 [file Data_Sheet_5.ZIP › code/output-bl-spp/62.JPG]

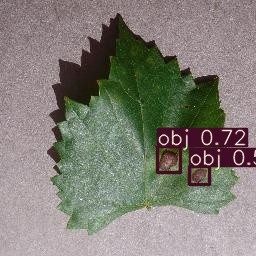

Supplement: Supplementary file 5 [file Data_Sheet_5.ZIP › code/output-bl-spp/63.JPG]

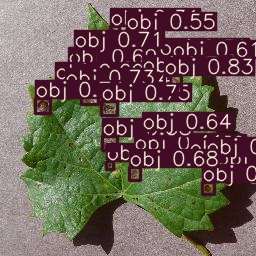

Supplement: Supplementary file 5 [file Data_Sheet_5.ZIP › code/output-bl-spp/64.JPG]

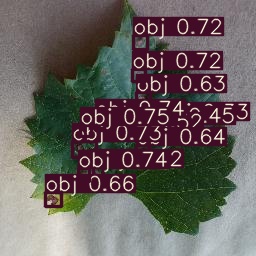

Supplement: Supplementary file 5 [file Data_Sheet_5.ZIP › code/output-bl-spp/65.JPG]

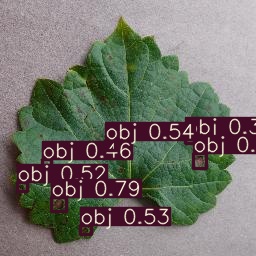

Supplement: Supplementary file 5 [file Data_Sheet_5.ZIP › code/output-bl-spp/66.JPG]

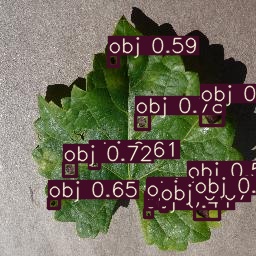

Supplement: Supplementary file 5 [file Data_Sheet_5.ZIP › code/output-bl-spp/67.JPG]

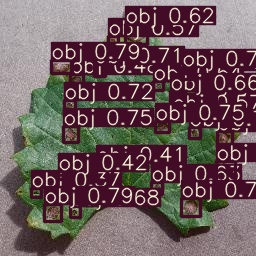

Supplement: Supplementary file 5 [file Data_Sheet_5.ZIP › code/output-bl-spp/68.JPG]

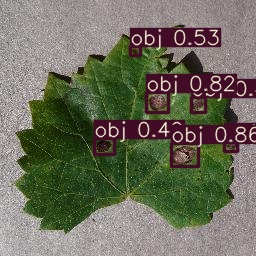

Supplement: Supplementary file 5 [file Data_Sheet_5.ZIP › code/output-bl-spp/69.JPG]

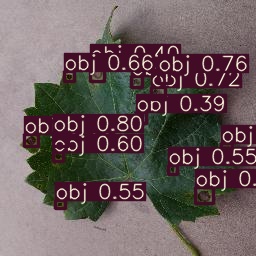

Supplement: Supplementary file 5 [file Data_Sheet_5.ZIP › code/output-bl-spp/7.JPG]

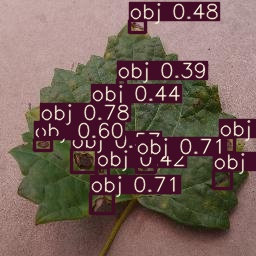

Supplement: Supplementary file 5 [file Data_Sheet_5.ZIP › code/output-bl-spp/70.JPG]

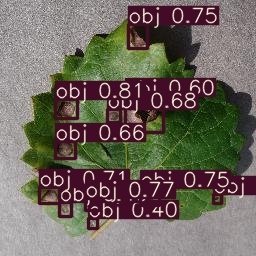

Supplement: Supplementary file 5 [file Data_Sheet_5.ZIP › code/output-bl-spp/71.JPG]

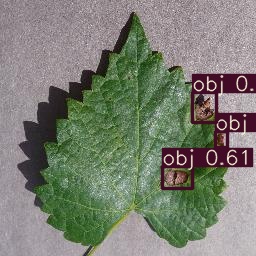

Supplement: Supplementary file 5 [file Data_Sheet_5.ZIP › code/output-bl-spp/72.JPG]

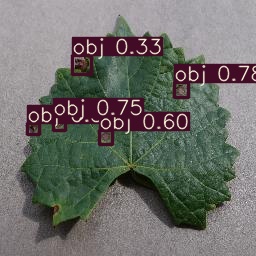

Supplement: Supplementary file 5 [file Data_Sheet_5.ZIP › code/output-bl-spp/73.JPG]

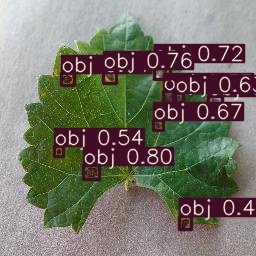

Supplement: Supplementary file 5 [file Data_Sheet_5.ZIP › code/output-bl-spp/74.JPG]

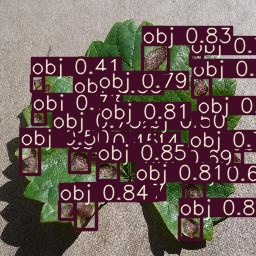

Supplement: Supplementary file 5 [file Data_Sheet_5.ZIP › code/output-bl-spp/75.JPG]

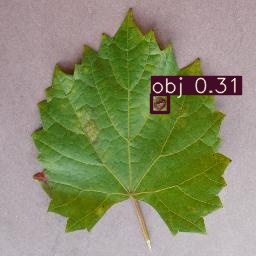

Supplement: Supplementary file 5 [file Data_Sheet_5.ZIP › code/output-bl-spp/76.JPG]

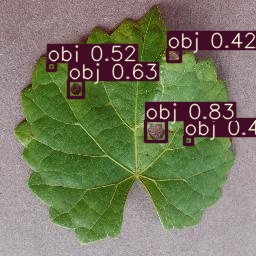

Supplement: Supplementary file 5 [file Data_Sheet_5.ZIP › code/output-bl-spp/77.JPG]

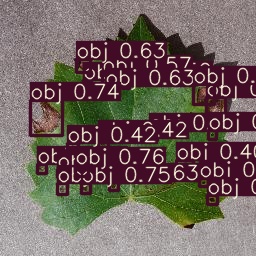

Supplement: Supplementary file 5 [file Data_Sheet_5.ZIP › code/output-bl-spp/78.JPG]

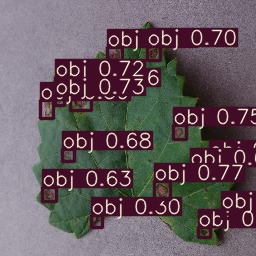

Supplement: Supplementary file 5 [file Data_Sheet_5.ZIP › code/output-bl-spp/79.JPG]

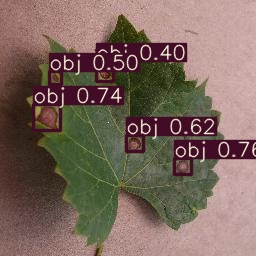

Supplement: Supplementary file 5 [file Data_Sheet_5.ZIP › code/output-bl-spp/8.JPG]

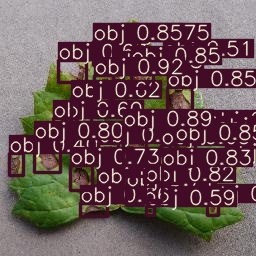

Supplement: Supplementary file 5 [file Data_Sheet_5.ZIP › code/output-bl-spp/80.JPG]

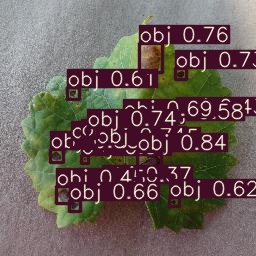

Supplement: Supplementary file 5 [file Data_Sheet_5.ZIP › code/output-bl-spp/81.JPG]

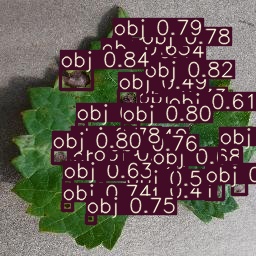

Supplement: Supplementary file 5 [file Data_Sheet_5.ZIP › code/output-bl-spp/82.JPG]

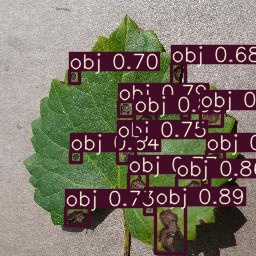

Supplement: Supplementary file 5 [file Data_Sheet_5.ZIP › code/output-bl-spp/83.JPG]

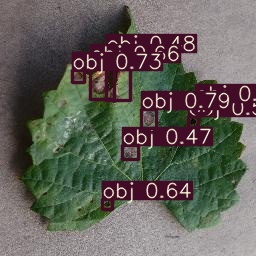

Supplement: Supplementary file 5 [file Data_Sheet_5.ZIP › code/output-bl-spp/84.JPG]

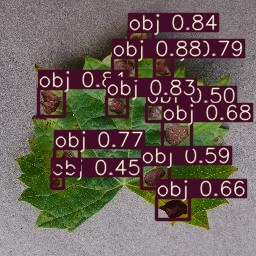

Supplement: Supplementary file 5 [file Data_Sheet_5.ZIP › code/output-bl-spp/85.JPG]

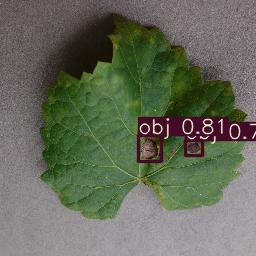

Supplement: Supplementary file 5 [file Data_Sheet_5.ZIP › code/output-bl-spp/86.JPG]

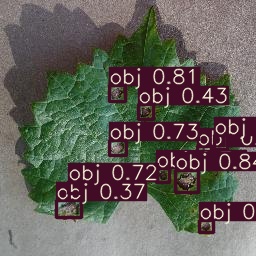

Supplement: Supplementary file 5 [file Data_Sheet_5.ZIP › code/output-bl-spp/87.JPG]

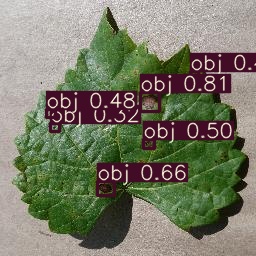

Supplement: Supplementary file 5 [file Data_Sheet_5.ZIP › code/output-bl-spp/88.JPG]

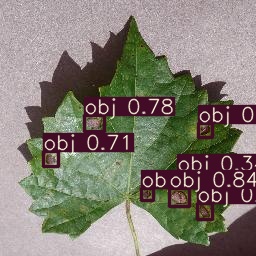

Supplement: Supplementary file 5 [file Data_Sheet_5.ZIP › code/output-bl-spp/89.JPG]
