# Supplementary figures and images for: Grape Leaf Black Rot Detection Based on Super-Resolution Image Enhancement and Deep Learning (part 6 of 6)
Source: Front Plant Sci. 2021 Jun 29;12:695749. doi: 10.3389/fpls.2021.695749 (PMC8277438; doi:10.3389/fpls.2021.695749)

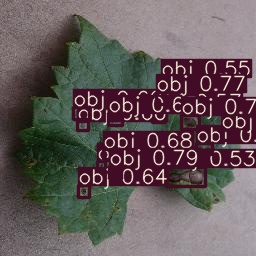

Supplement: Supplementary file 5 [file Data_Sheet_5.ZIP › code/output-bl-spp/9.JPG]

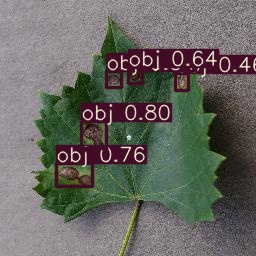

Supplement: Supplementary file 5 [file Data_Sheet_5.ZIP › code/output-bl-spp/90.JPG]

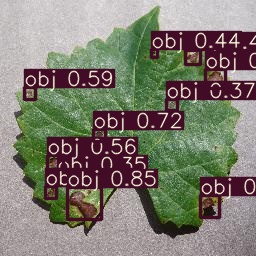

Supplement: Supplementary file 5 [file Data_Sheet_5.ZIP › code/output-bl-spp/91.JPG]

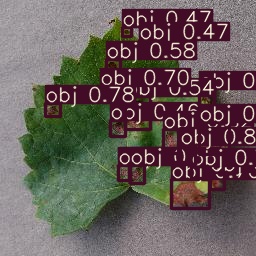

Supplement: Supplementary file 5 [file Data_Sheet_5.ZIP › code/output-bl-spp/92.JPG]

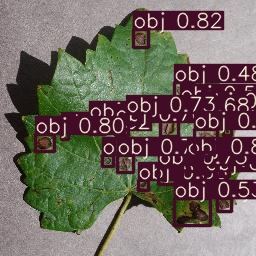

Supplement: Supplementary file 5 [file Data_Sheet_5.ZIP › code/output-bl-spp/93.JPG]

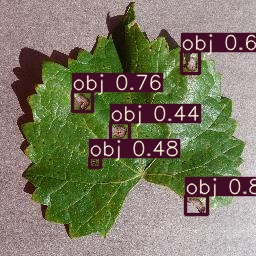

Supplement: Supplementary file 5 [file Data_Sheet_5.ZIP › code/output-bl-spp/94.JPG]

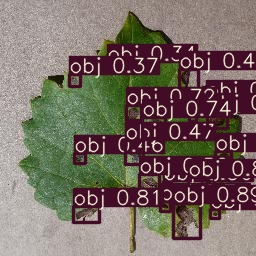

Supplement: Supplementary file 5 [file Data_Sheet_5.ZIP › code/output-bl-spp/95.JPG]

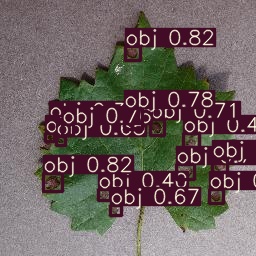

Supplement: Supplementary file 5 [file Data_Sheet_5.ZIP › code/output-bl-spp/96.JPG]

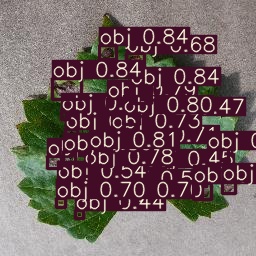

Supplement: Supplementary file 5 [file Data_Sheet_5.ZIP › code/output-bl-spp/97.JPG]

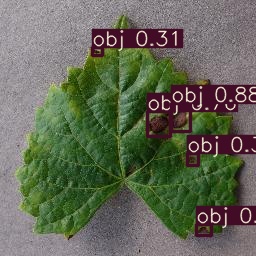

Supplement: Supplementary file 5 [file Data_Sheet_5.ZIP › code/output-bl-spp/98.JPG]

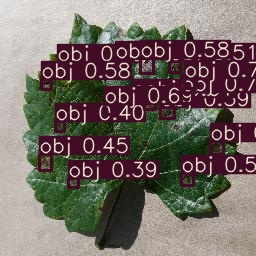

Supplement: Supplementary file 5 [file Data_Sheet_5.ZIP › code/output-bl-spp/99.JPG]

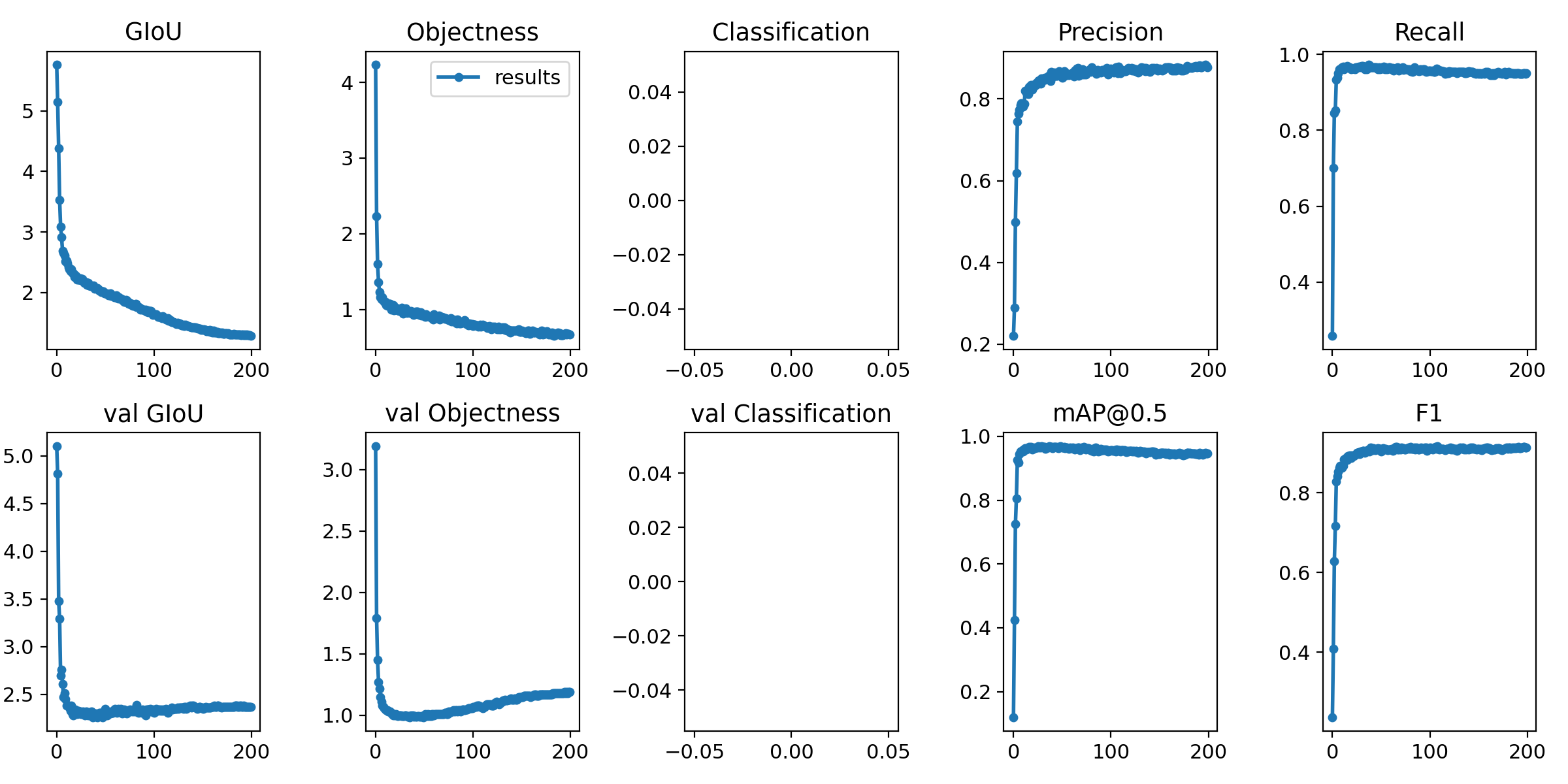

Supplement: Supplementary file 5 [file Data_Sheet_5.ZIP › code/result/200-bl/results.png]

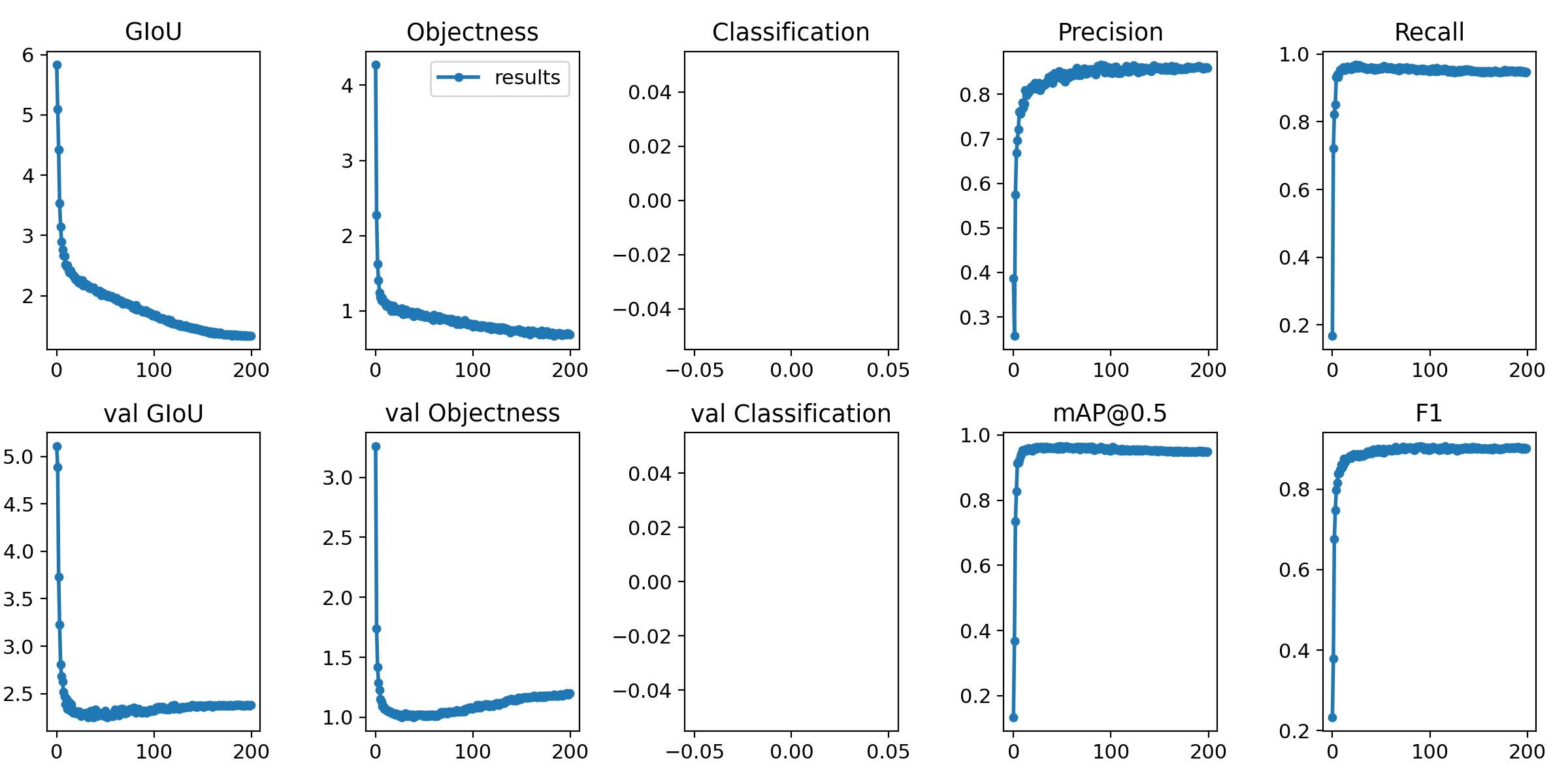

Supplement: Supplementary file 5 [file Data_Sheet_5.ZIP › code/result/200-nn/results.png]

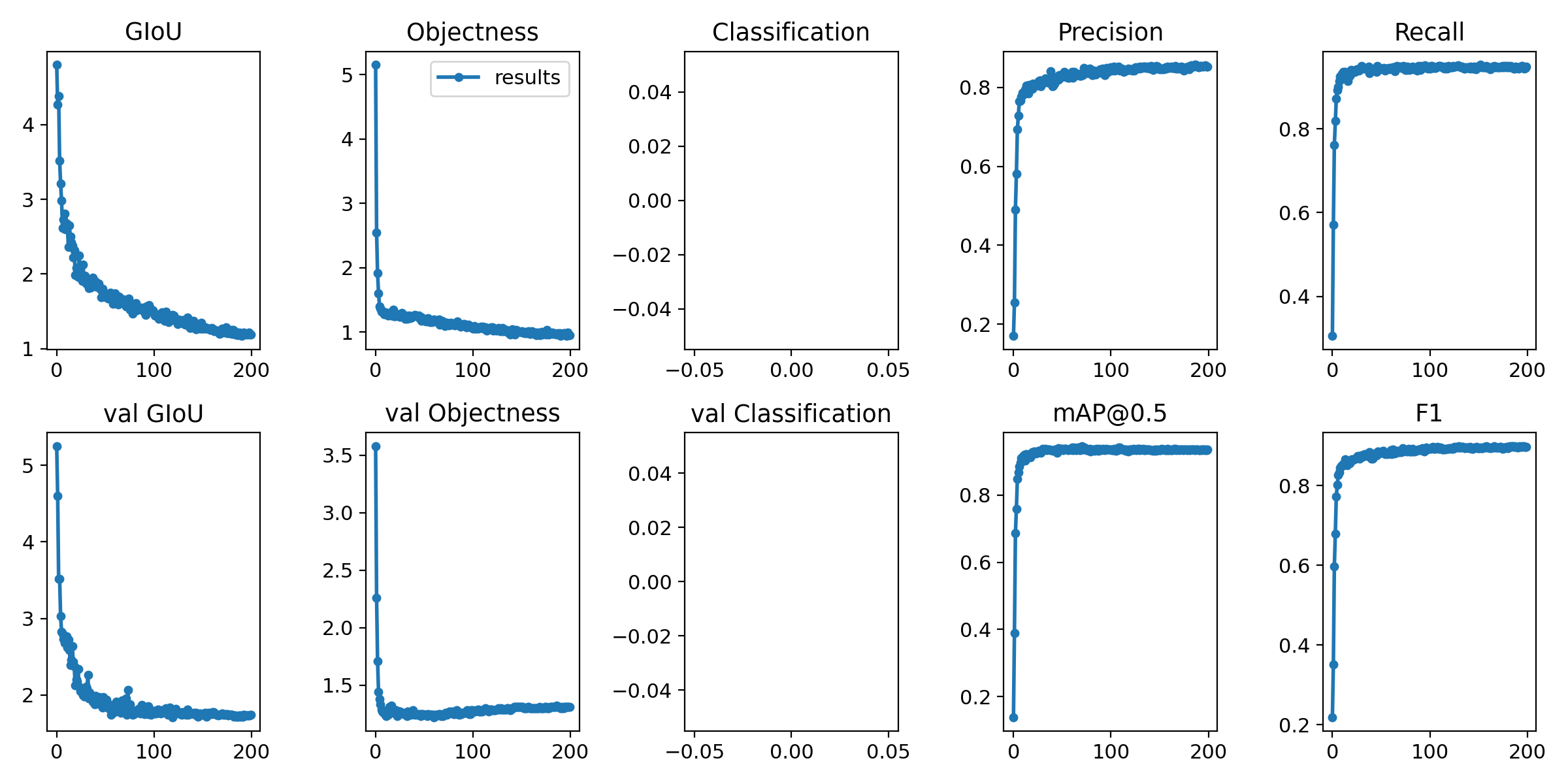

Supplement: Supplementary file 5 [file Data_Sheet_5.ZIP › code/result/200-yuan-spp/results.png]

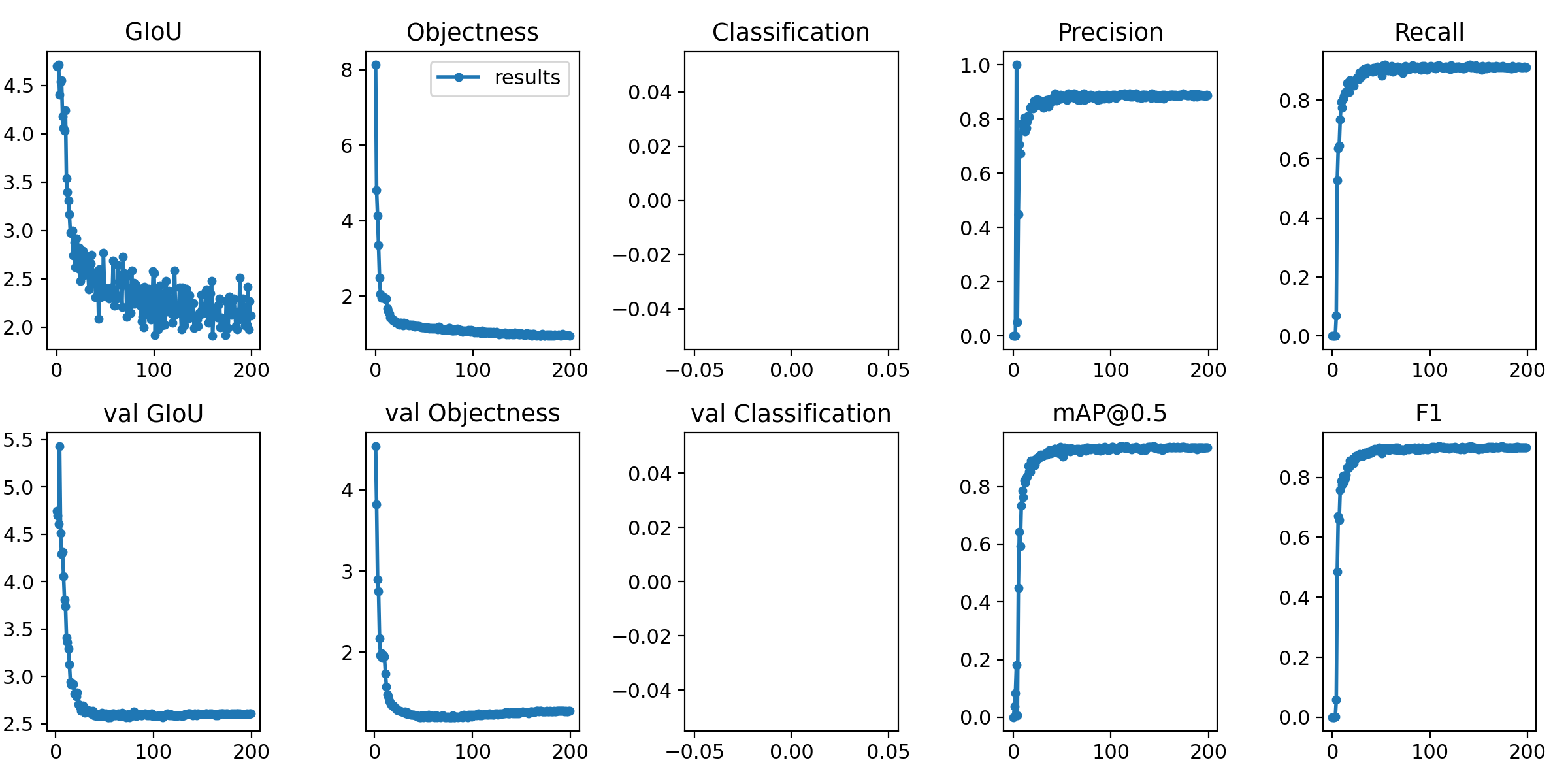

Supplement: Supplementary file 5 [file Data_Sheet_5.ZIP › code/result/200-yuan-v3/results.png]
